# Supplementary material for: Selective tubulin-binding drugs induce pericyte phenotype switching and anti-cancer immunity
Source: EMBO Mol Med. 2025 Mar 26;17(5):1071–100. doi: 10.1038/s44321-025-00222-6 (PMC12081767; doi:10.1038/s44321-025-00222-6)
Supplement: Supplementary file 1 — Appendix [file 44321_2025_222_MOESM1_ESM.pdf]

Table of content of the Appendix

page

- Appendix Table S1……………………………………………………………….2
- Appendix Table S2……………………………………………………………….3
- Appendix Table S3……………………………………………………………….4
- Appendix Figure S1………………………………………………………………5

Appendix Table S1. Patient data for breast cancer biopsies analyzed in Figure 8.

Patient ID	Age	Clinical Response	Histological Grade	Sum Lesion (baseline)	Sum Lesion (pre-surgery)	PAM50	PAM50 Change
**HR+ Breast Cancer Patients**							
**401011**	52.02	PR	G1	25	14	LA	LA - LA
**401012**	62.58	PR	G2	22	10	LA	LB - LA
**401017**	77.77	SD	G2	25	20	LB	LB - LB
**401022**	36.02	SD	G2	57	57	LB	LB - LA
**401028**	72.81	PR	G3	34	18	LB	LB - LB
**401030**	69.64	PR	G2	36	24	LB	LB - LB
**410005**	49.75	SD	G3	26	19	LB	LB – LA-LB
**410007**	52.92	PR	G3	27	11	LB	LB - LB
**TNBC Patients**							
**401016**	52.84	PR	G3	28	17	B	LB - LB
**401021**	52.92	PD	G3	36	30	B	B - B
**401029**	72.51	PR	G3	40	14	LB	LB - N
**401036**	73.88	SD	G3	40	36	H2	H2 - H2
**402005**	47.93	SD	Gx	20	18	B	B - B
**406002**	51.92	PD	G3	46	38	B	B - B
**412006**	58.34	PD	G2	45	55	B	B - B
**401033**	41.65	SD	G2	22	16	B	B - B

Appendix Table S2: Antibodies for Western Blot and FACS

Name	Clone	Color	Dilution	Cat #	Source	Antibody ID
**Western Blot**						
**ACTG2**	polyclonal	-	1:1000	BS1004	Bioworld	AB_1664062
**CNN1**	EP798Y	-	1:1000	46794	Abcam	AB_2291941
**pAKT**	D93	-	1:1000	4060	Cell Signalling Technology	AB_2315049
**pMAPK**	D13.14.4E	-	1:1000	4370	Cell Signalling Technology	AB_2315112
**pMLC**	polyclonal	-	1:1000	ab2480	Abcam	AB_303094
**TUBA1A**	B-5-1-2	-	1:5000	T6074	Sigma	AB_477582
**FACS**						
**B220**	RA3-6B2	BV750	1:100	103261	BioLegend	AB_2734157
**CD3e**	eBio500A2	PerCp-eFlour 710	1:100	46-0033-82	Thermo Fisher Scientific	AB_10597122
**CD4**	RM4-5	APC Fire 750	1:100	100567	BioLegend	AB_2629698
**CD4**	A15.1.17	PE	1:100	553653	BD Biosciences	AB_394973
**CD8**	53-6.7	APC-Cy7	1:500	557654	BD Biosciences	AB_396769
**CD8**	53-6-7	PE	1:100	553033	BD Biosciences	AB_394571
**CD11 b**	M1/70	APC-Cy7	1:1000	101226	BioLegend	AB_830642
**CD25**	PC61	PE-Cy7	1:100	552880	BD Biosciences	AB_394509
**CD44**	IM7	PE-Cy7	1:500	103030	BioLegend	AB_830787
**CD45**	30F-11	PE-CF594	1:2000	562420	BD Biosciences	AB_11154401
**CD45**	30F-11	BV510	1:100	103137	BioLegend	AB_2561392
**CD45.1**	A20	BUV421	1:100	563983	BD Biosciences	AB_2738523
**CD45.2**	104	PE-Cy7	1:150	560696	BD Biosciences	AB_1727494
**CD69**	H1.2F3	PE-CF594	1:2000	562455	BD Biosciences	AB_11154217
**CD103**	2E7	APC	1:150	121414	BioLegend	AB_1227502
**CD206**	C068C2	PE-Cy7	1:150	141720	BioLegend	AB_2562248
**F4/80**	BM8	APC	1:150	123116	BioLegend	AB_893481
**FoxP3**	150D	AF647	1:100	320014	BioLegend	AB_439750
**Gr-1**	RB6-8C5	PE	1:100	108408	BioLegend	AB_313373
**Granzyme B**	GB11	AF647	1:100	560212	BD Biosciences	AB_11154033
**iNOS**	CXNFT	AF-488	1:100	53-5920-82	Thermo Fisher Scientific	AB_2574423
**Ki67**	B56	PE-Cy7	1:100	561283	BD Biosciences	AB_10716060
**MHCclass II (I-A/I-E)**	M5/114.15.2	PerCP-Cy5.5	1:50	107626	BioLegend	AB_2191071
**TCRv2α**	B20.1	APC	1:150	127810	BioLegend	AB_1089250

Appendix Table S3: Antibodies for Immunohistochemistry and Secondary Antibodies

Name	Host Species	Clone	Color	Cat #	Source	Antibody ID
**Immunohistochemistry**						
**ACTG2**	rabbit	polyclonal	-	AP06002PU-N	Origene	AB_1610944
**α-SMA**	mouse	1A4	FITC	F3777	Sigma	AB_476977
**CD31**	mouse	JC/70A	-	ab9498	Abcam	AB_307284
**CD31**	rat	MEC13.3	-	550274	BD Biosciences	AB_393571
**CD31-biotin**	rat	MEC13.3	-	553371	BD Biosciences	AB_394817
**CD144 (CDH5)**	rabbit	BLR091G	-	MA544373	Thermo Fisher Scientific	AB_2926503
**CD144 (CDH5)**	rat	11D4.1	-	555289	BD Biosciences	AB_395707
**CNN1**	rabbit	EP7998Y	-	ab46794	Abcam	AB_2291941
**COLI**	rabbit	polyclonal	-	PAB13488	Abnova	AB_10556808
**ERK, phospho**	rabbit	D13.14.4E	-	4370S	Cell Signaling Technology	AB_2315112
**ICAM**	hamster	3E2B	-	MA5405	Invitrogen	AB_223595
**NG2**	rabbit	polyclonal	-	AB5320	Millipore	AB_91789
**NG2**	rat	1E6.4	-	130-097-455	Miltenyi Biotec	AB_2651235
**pAKT**	rabbit	D93	-	4060	Cell Signalling Technology	AB_2315049
**pMLC (phospho S20)**	rabbit	polyclonal	-	ab2480	Abcam	AB_303094
**pS6R**	rabbit	D57.2.2E	-	4858S	Cell Signaling Technology	AB_916156
**PDGFR**	rabbit	Y92	-	ab32570	Abcam	AB_777135
**Secondary Antibodies**						
**anti-goat IgG AF488**	**donkey**	polyclonal	AF488	ab150129	Abcam	AB_2687506
**anti-hamster IgG AF488**	**goat**	polyclonal	AF488	A21110	Thermo Fisher Scientific	AB_2535759
**anti-hamster IgG Cy3**	**rabbit**	polyclonal	Cy3	307-165-003	Jackson ImmunoResearch	AB_2339586
**anti-mouse IgG Dylight 405**	**goat**	polyclonal	Dylight 405	115-475-003	Jackson ImmunoResearch	AB_2338786
**anti-rabbit IgG DyLight 405**	**goat**	polyclonal	DyLight 405	111-475-003	Jackson ImmunoResearch	AB_2338035
**anti-mouse IgG HRP**	**horse**	polyclonal	HRP	PI-2000	Vector	AB_2336177
**anti-rabbit IgG AF488**	**donkey**	polyclonal	AF488	A21206	Thermo Fisher Scientific	AB_243579
**anti-rabbit IgG AF594**	**donkey**	polyclonal	AF594	A21207	Thermo Fisher Scientific	AB_141637
**anti-rabbit IgG HRP**	**goat**	polyclonal	HRP	PI-1000	Vector	AB_1000
**anti-rat IgG AF488**	**donkey**	polyclonal	AF488	A21208	Thermo Fisher Scientific	AB_2535794
**anti-rat IgG AF594**	**donkey**	polyclonal	AF459	A21209	Thermo Fisher Scientific	AB_2435795
**anti-rat IgG biotin**	**donkey**	polyclonal	biotin	A18749	Thermo Fisher Scientific	AB_2535526
**Anti-rat IgG Dylight 405**	**donkey**	polyclonal	Dylight 405	712-475-153	Jackson ImmunoResearch	AB_2340681
**Streptavidin-AF488**	**-**	-	AF488	016-540-084	Jackson ImmunoResearch	AB_2337249

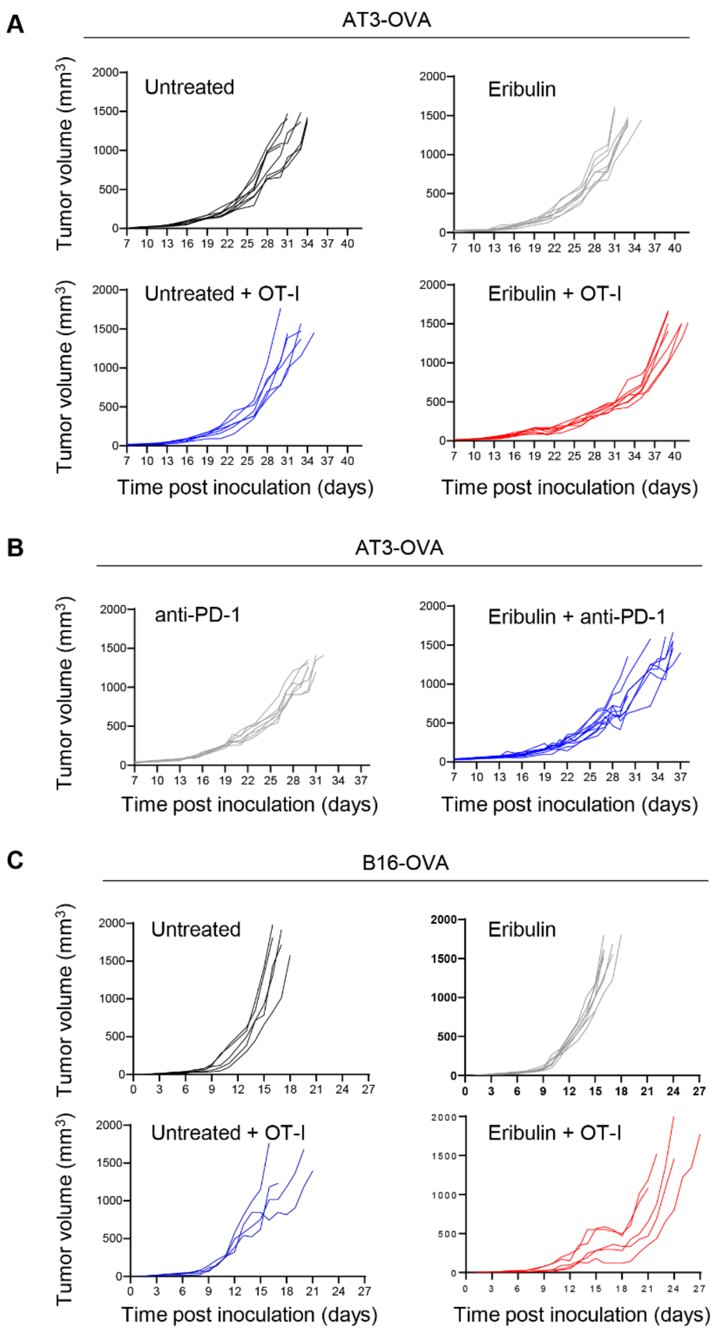

Appendix Figure S1. Eribulin acts synergistically with immunotherapy in OVA-expressing breast cancer and melanoma.

(**A**) Individual growth curves of AT3-OVA breast cancer as shown in Figure 6F for the following experimental groups: untreated, eribulin, untreated + OT-I T cell transfer and eribulin + OT-I. (**B**) Individual growth curves of AT3-OVA breast cancer as shown in Figure 6G for the following experimental groups: anti-PD-1 and eribulin + anti-PD-1. (**C**) Individual growth curves of B16-OVA melanoma as shown in Figure 7C for untreated, eribulin, untreated + OT-I and eribulin + OT-I treatment groups.
