# Supplementary figures and images for: Selective tubulin-binding drugs induce pericyte phenotype switching and anti-cancer immunity
Source: EMBO Mol Med. 2025 Mar 26;17(5):1071–100. doi: 10.1038/s44321-025-00222-6 (PMC12081767; doi:10.1038/s44321-025-00222-6)

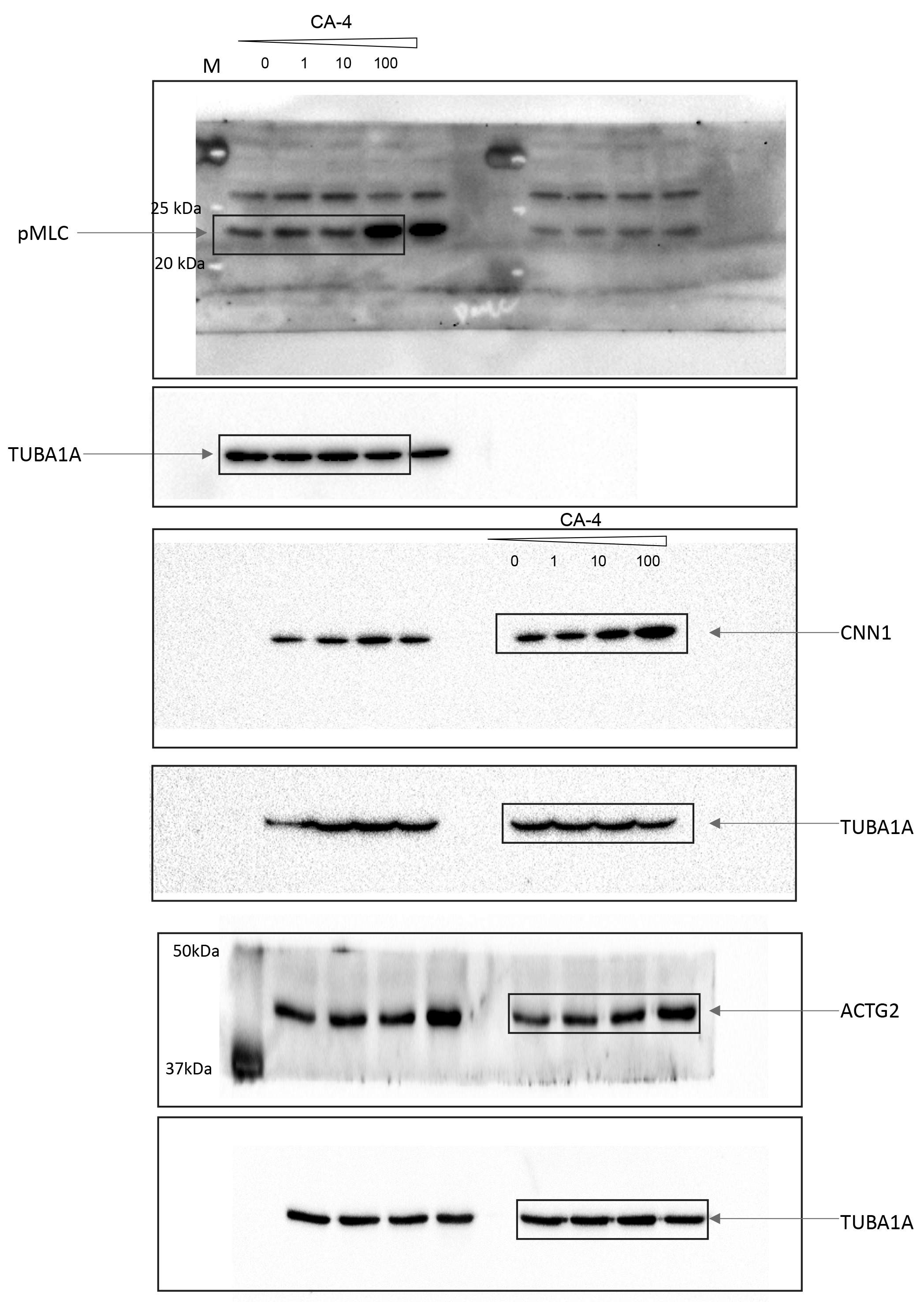

Supplement: Supplementary file 3 — Source data Fig. 1 [file 44321_2025_222_MOESM3_ESM.zip › For EMM submission/Figure 1B/CA-4 WB 1 (as in figure).tif]

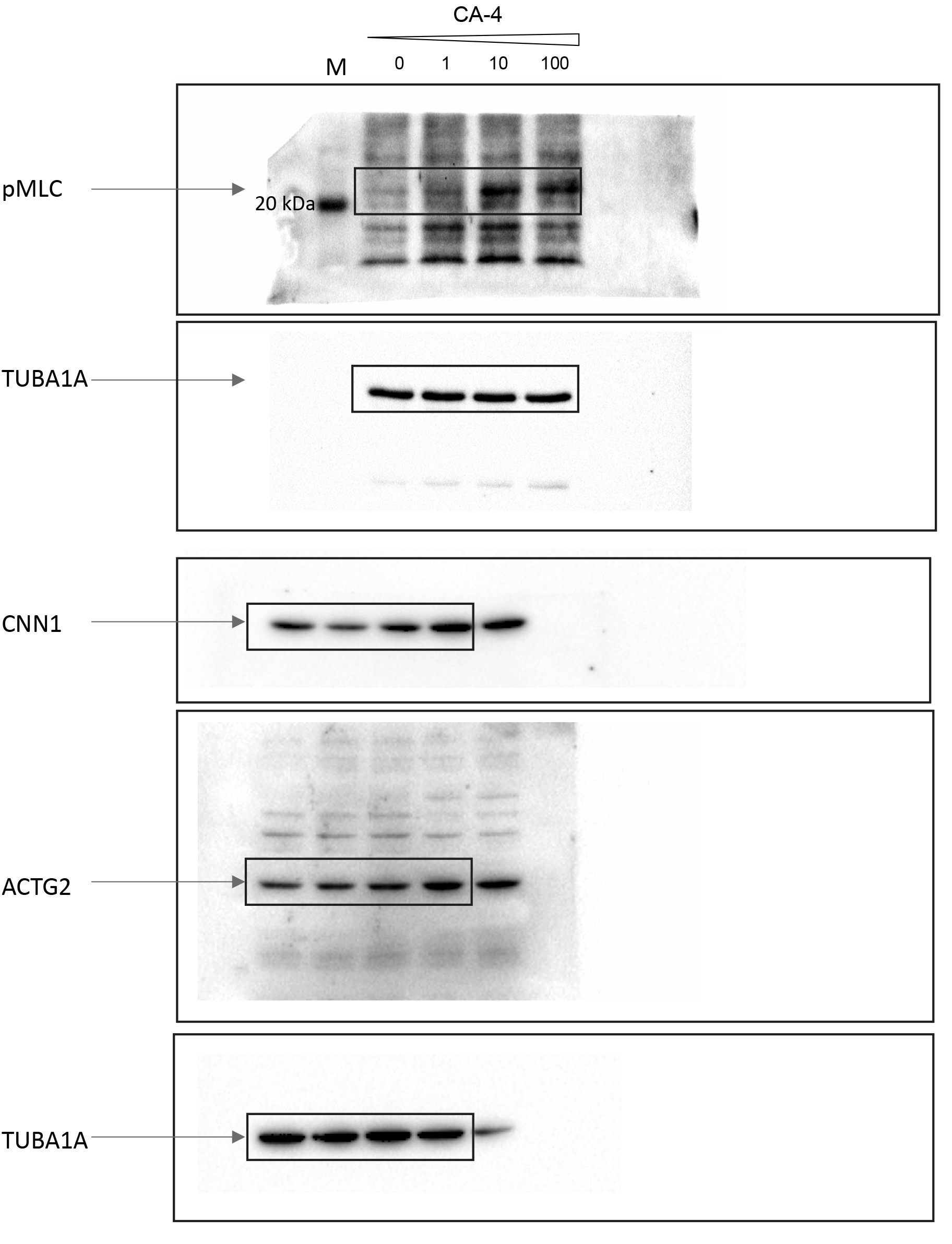

Supplement: Supplementary file 3 — Source data Fig. 1 [file 44321_2025_222_MOESM3_ESM.zip › For EMM submission/Figure 1B/CA-4 WB 2 repeat.tif]

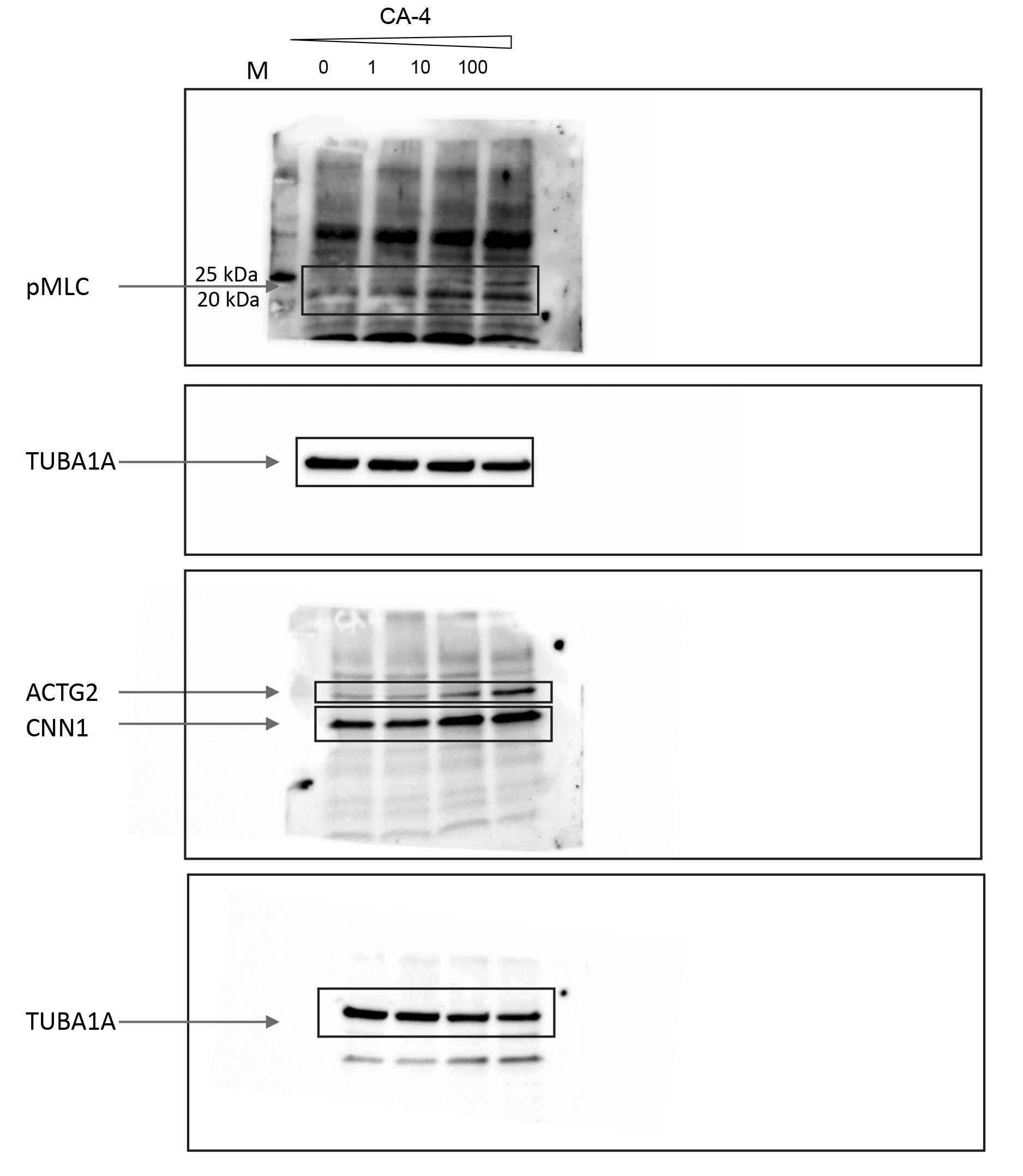

Supplement: Supplementary file 3 — Source data Fig. 1 [file 44321_2025_222_MOESM3_ESM.zip › For EMM submission/Figure 1B/CA-4 WB 3 repeat.tif]

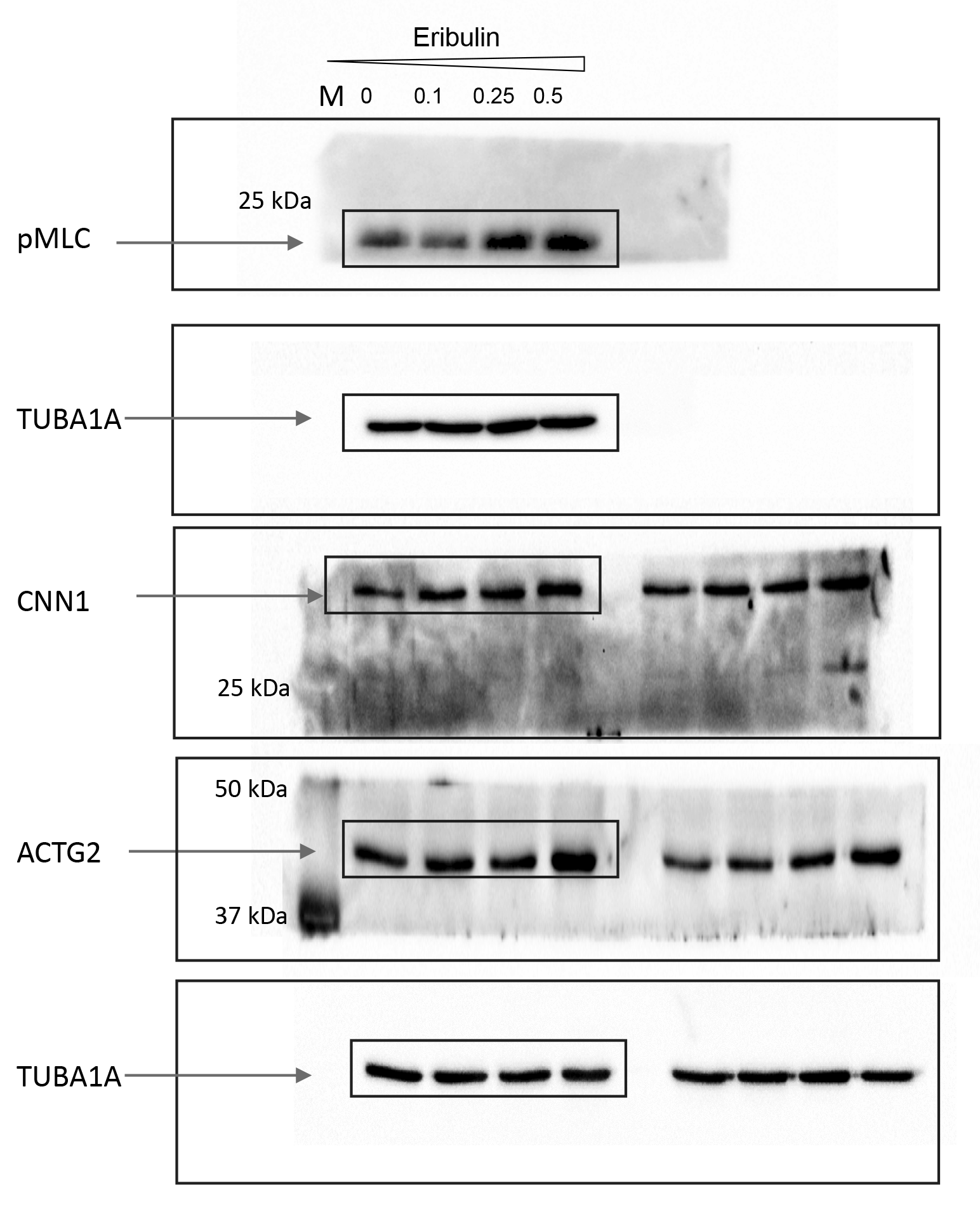

Supplement: Supplementary file 3 — Source data Fig. 1 [file 44321_2025_222_MOESM3_ESM.zip › For EMM submission/Figure 1C/Eribulin WB-1.tif]

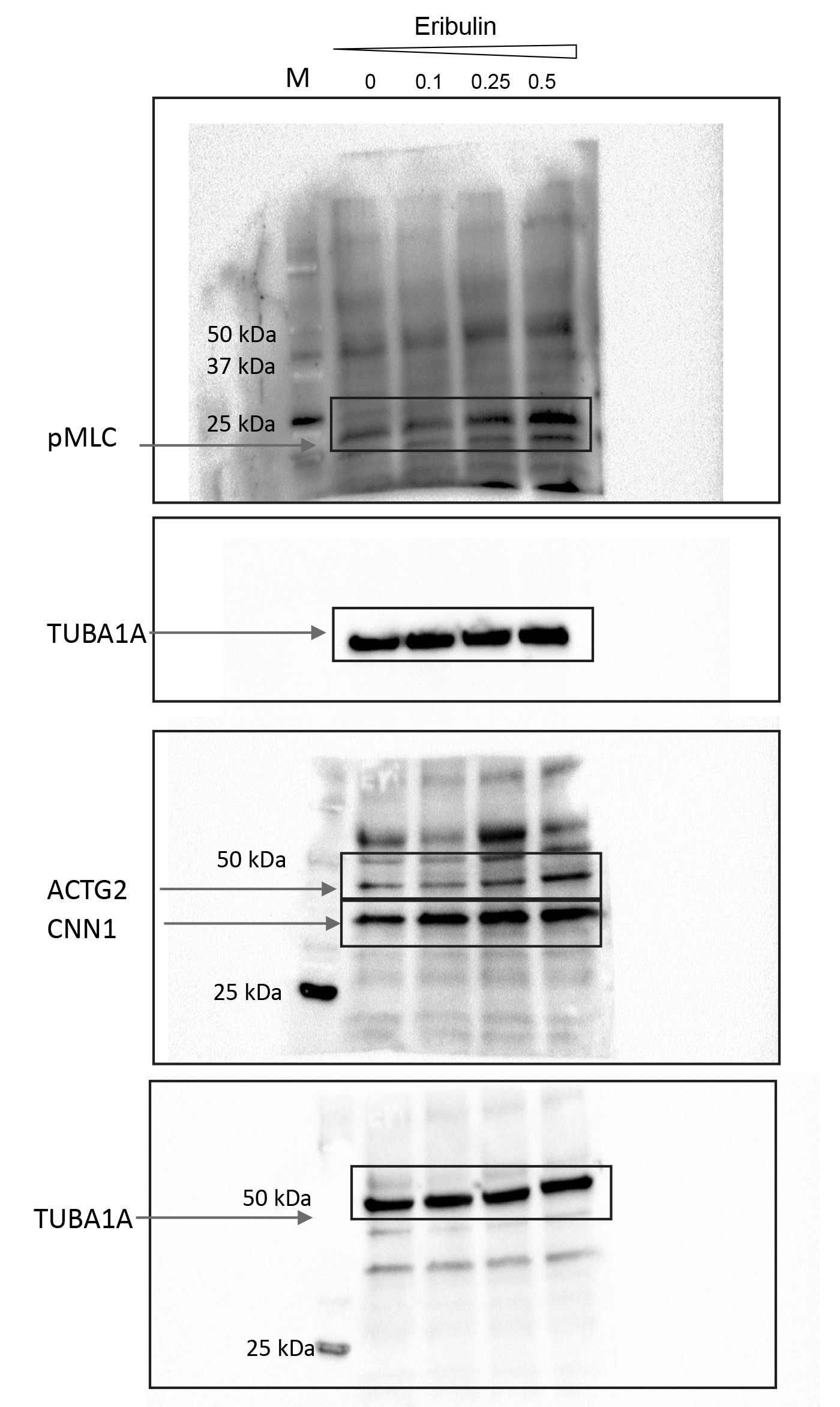

Supplement: Supplementary file 3 — Source data Fig. 1 [file 44321_2025_222_MOESM3_ESM.zip › For EMM submission/Figure 1C/Eribulin WB-2 repeat.tif]

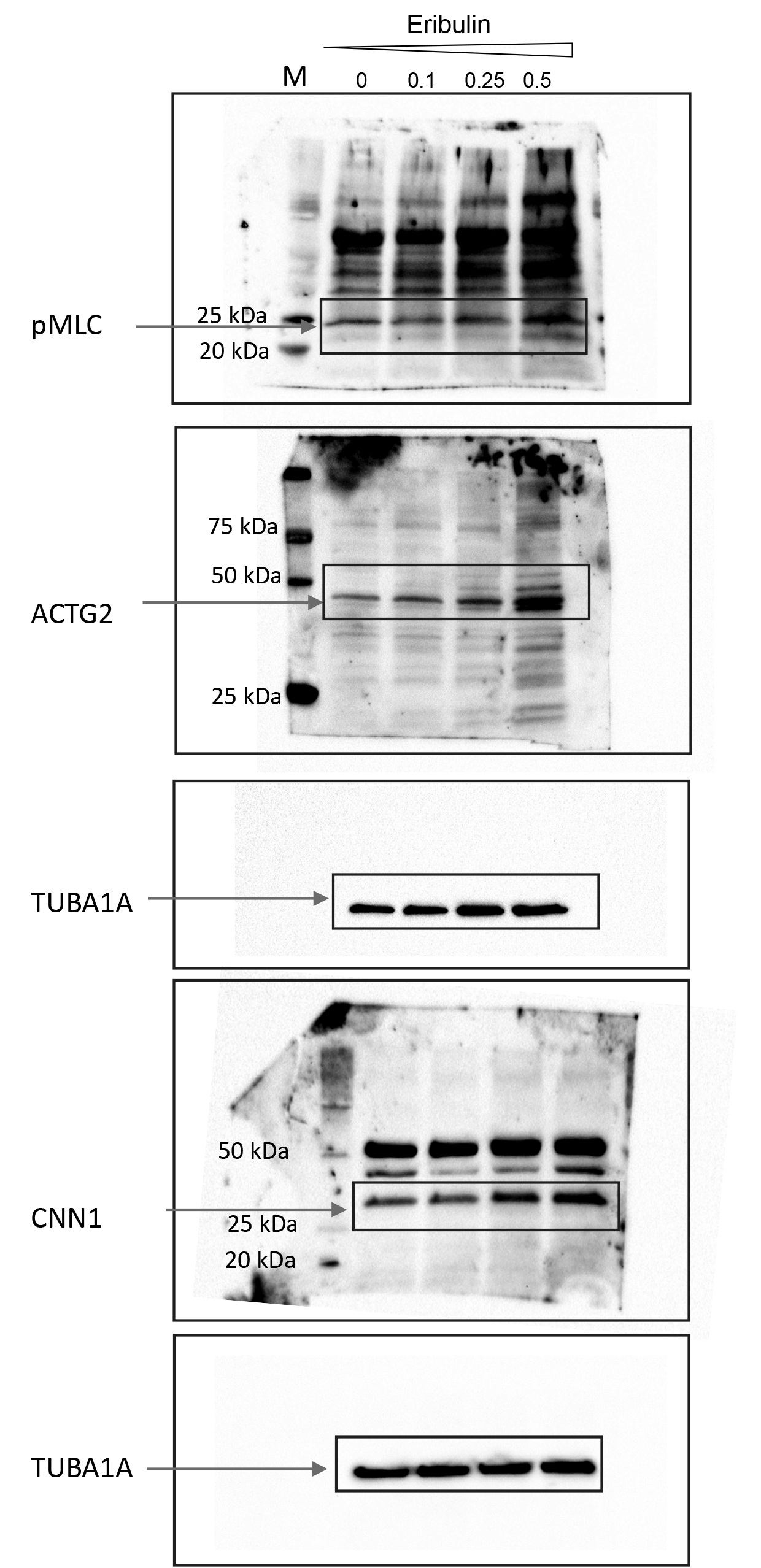

Supplement: Supplementary file 3 — Source data Fig. 1 [file 44321_2025_222_MOESM3_ESM.zip › For EMM submission/Figure 1C/Eribulin WB-3 repeat (as in figure).tif]

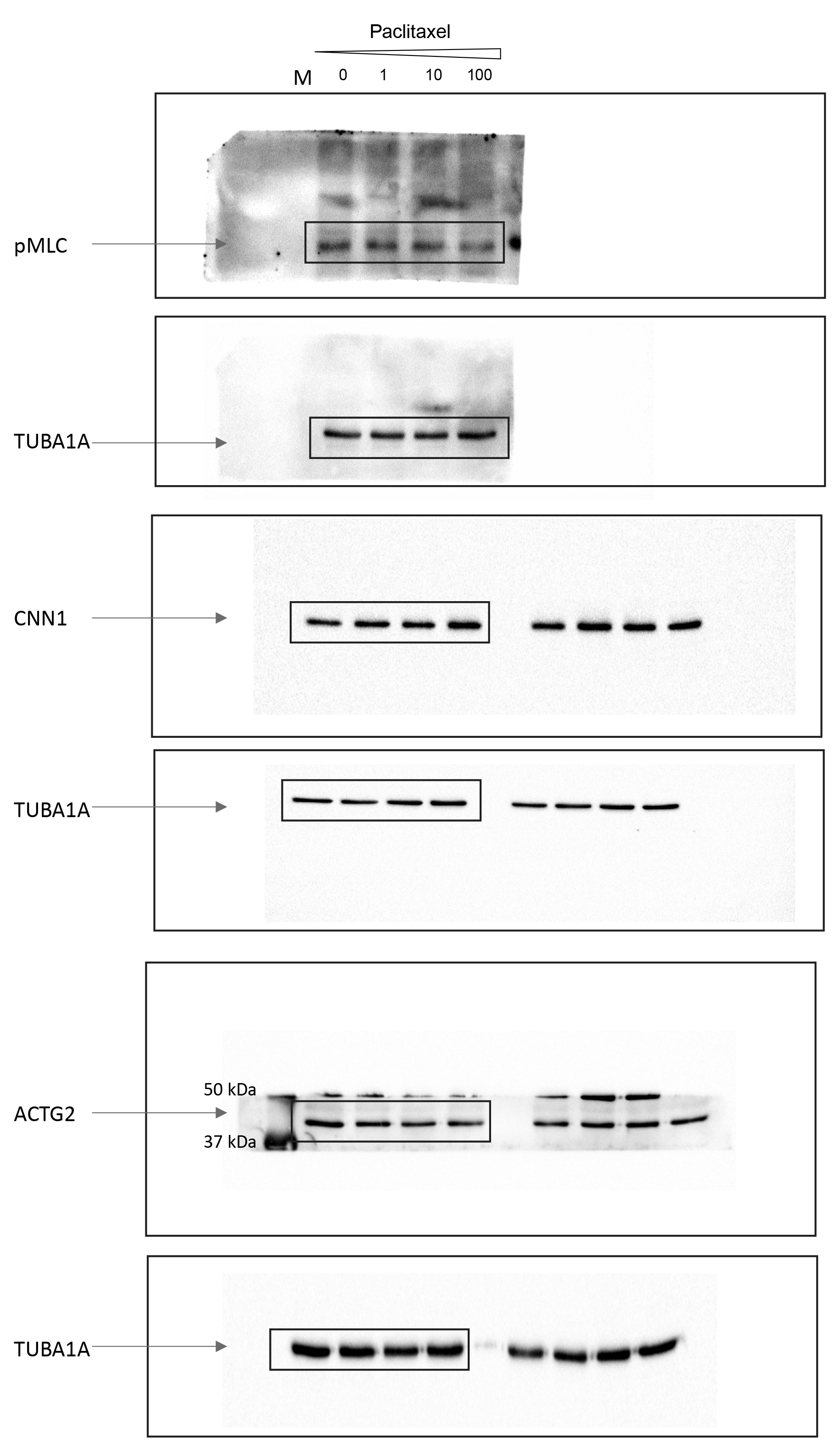

Supplement: Supplementary file 3 — Source data Fig. 1 [file 44321_2025_222_MOESM3_ESM.zip › For EMM submission/Figure 1D/Paclitaxel WB 1 (as in figure).tif]

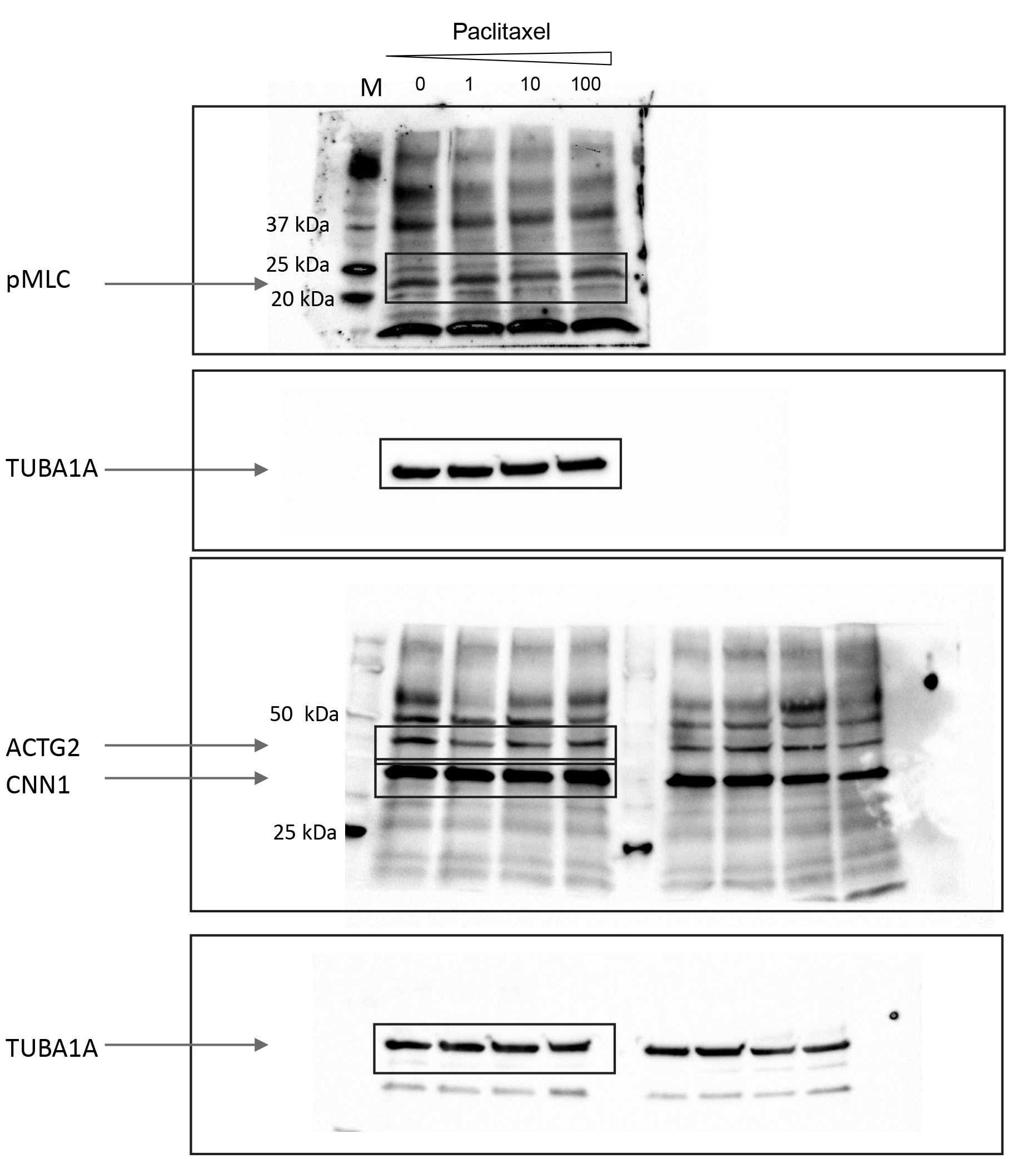

Supplement: Supplementary file 3 — Source data Fig. 1 [file 44321_2025_222_MOESM3_ESM.zip › For EMM submission/Figure 1D/Paclitaxel WB 2 repeat.tif]

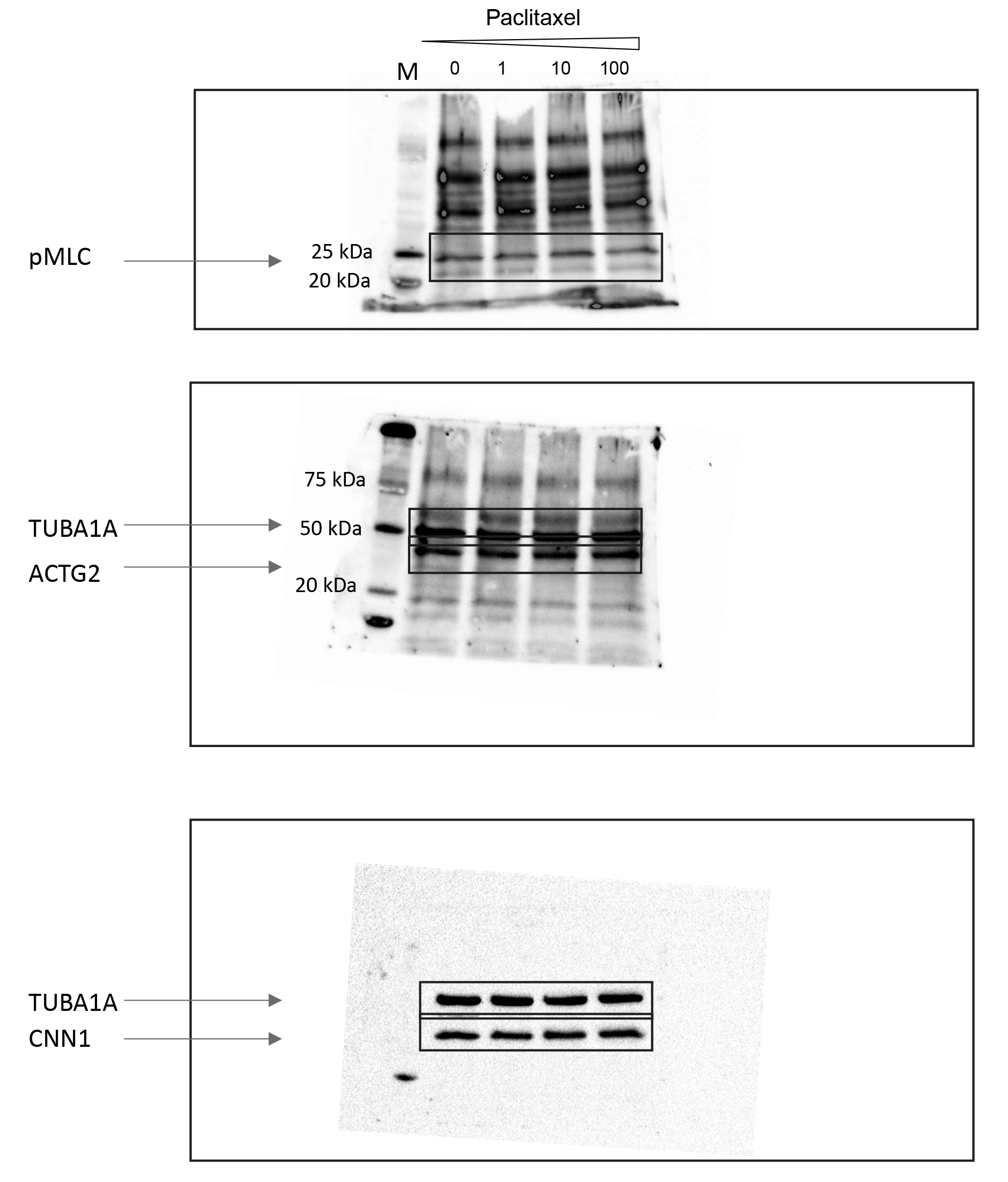

Supplement: Supplementary file 3 — Source data Fig. 1 [file 44321_2025_222_MOESM3_ESM.zip › For EMM submission/Figure 1D/Paclitaxel WB 3 repeat.tif]

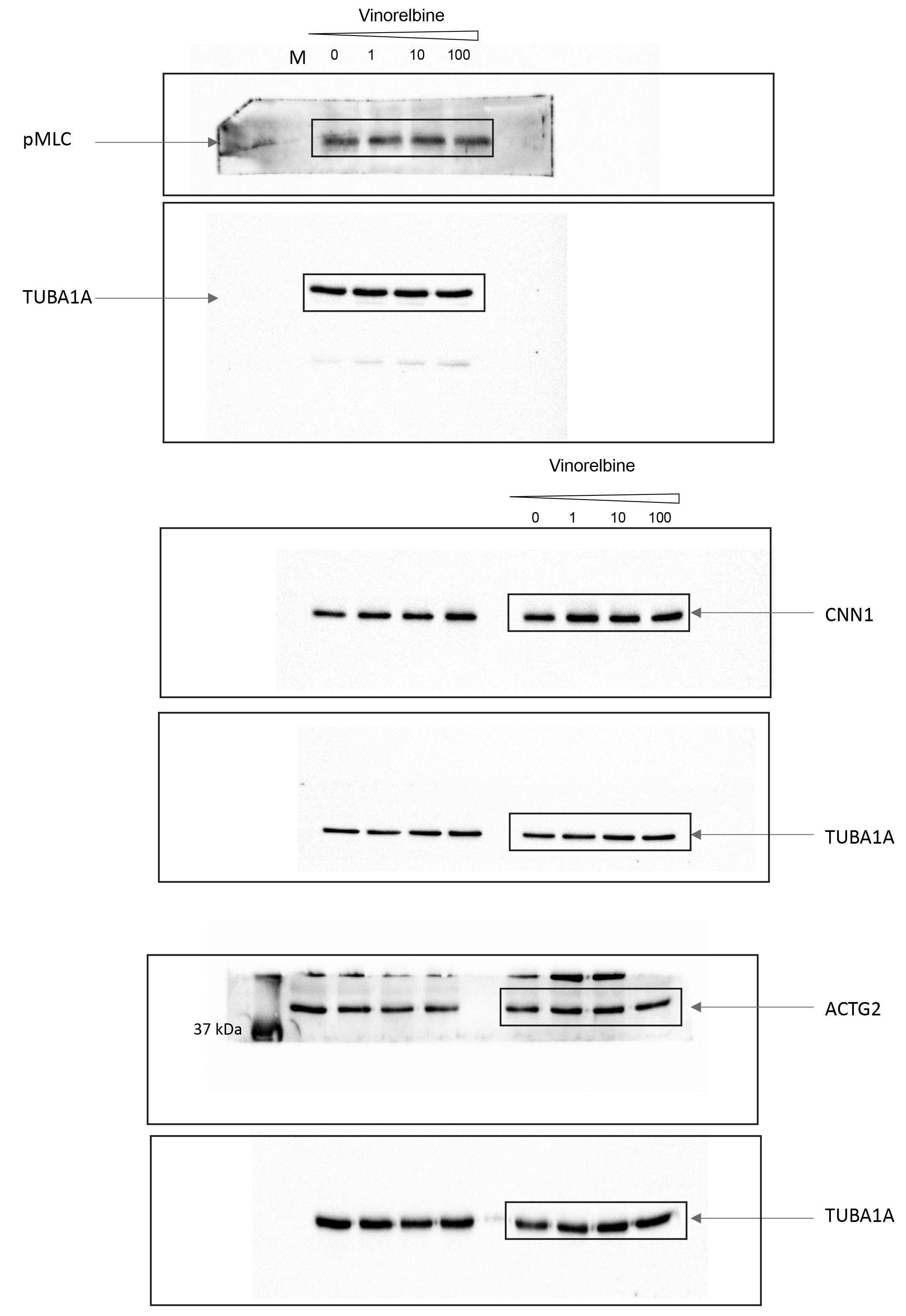

Supplement: Supplementary file 3 — Source data Fig. 1 [file 44321_2025_222_MOESM3_ESM.zip › For EMM submission/Figure 1E/Vinorelbine WB 1 (as in figure).tif]

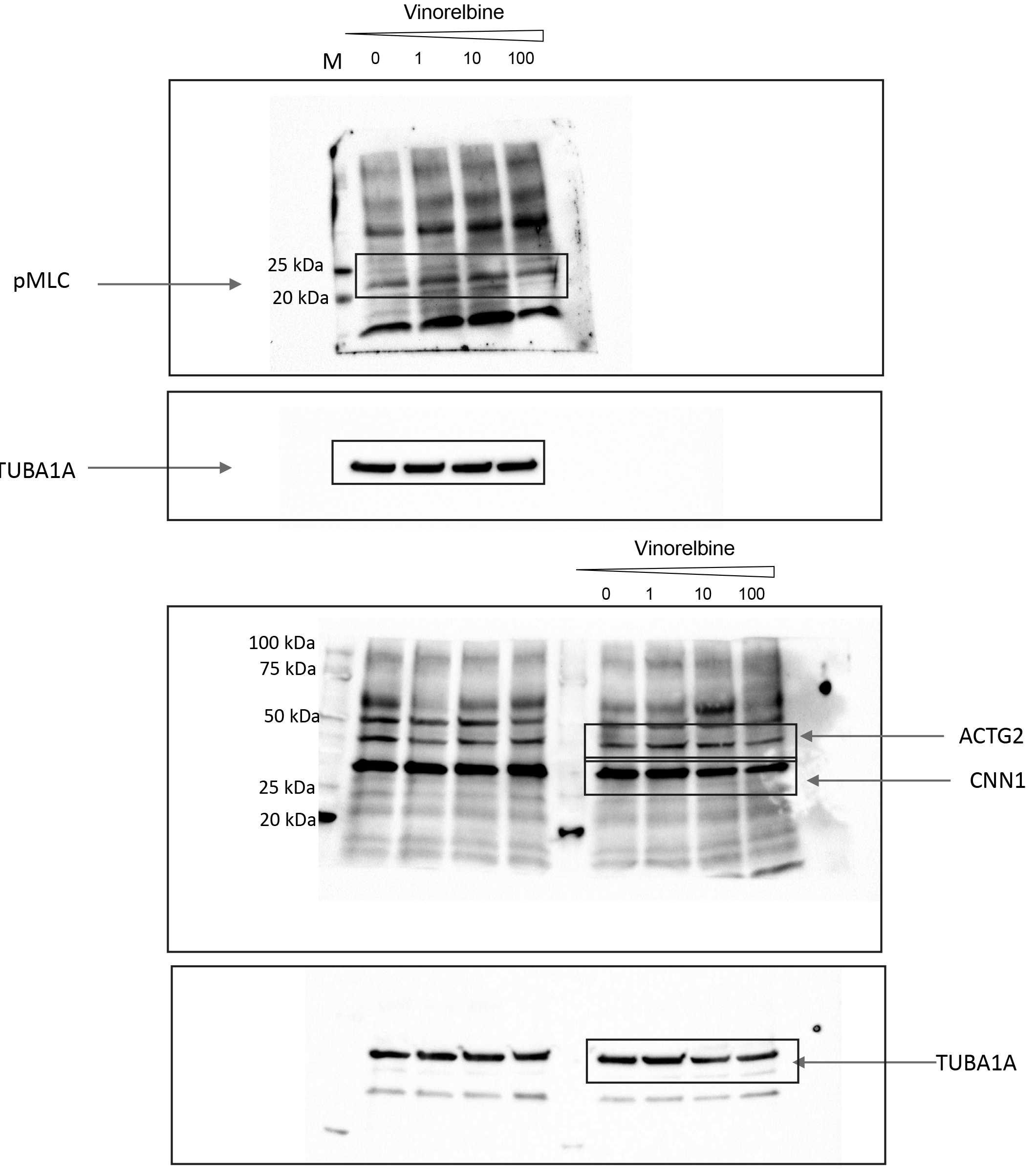

Supplement: Supplementary file 3 — Source data Fig. 1 [file 44321_2025_222_MOESM3_ESM.zip › For EMM submission/Figure 1E/Vinorelbine WB 2 repeat.tif]

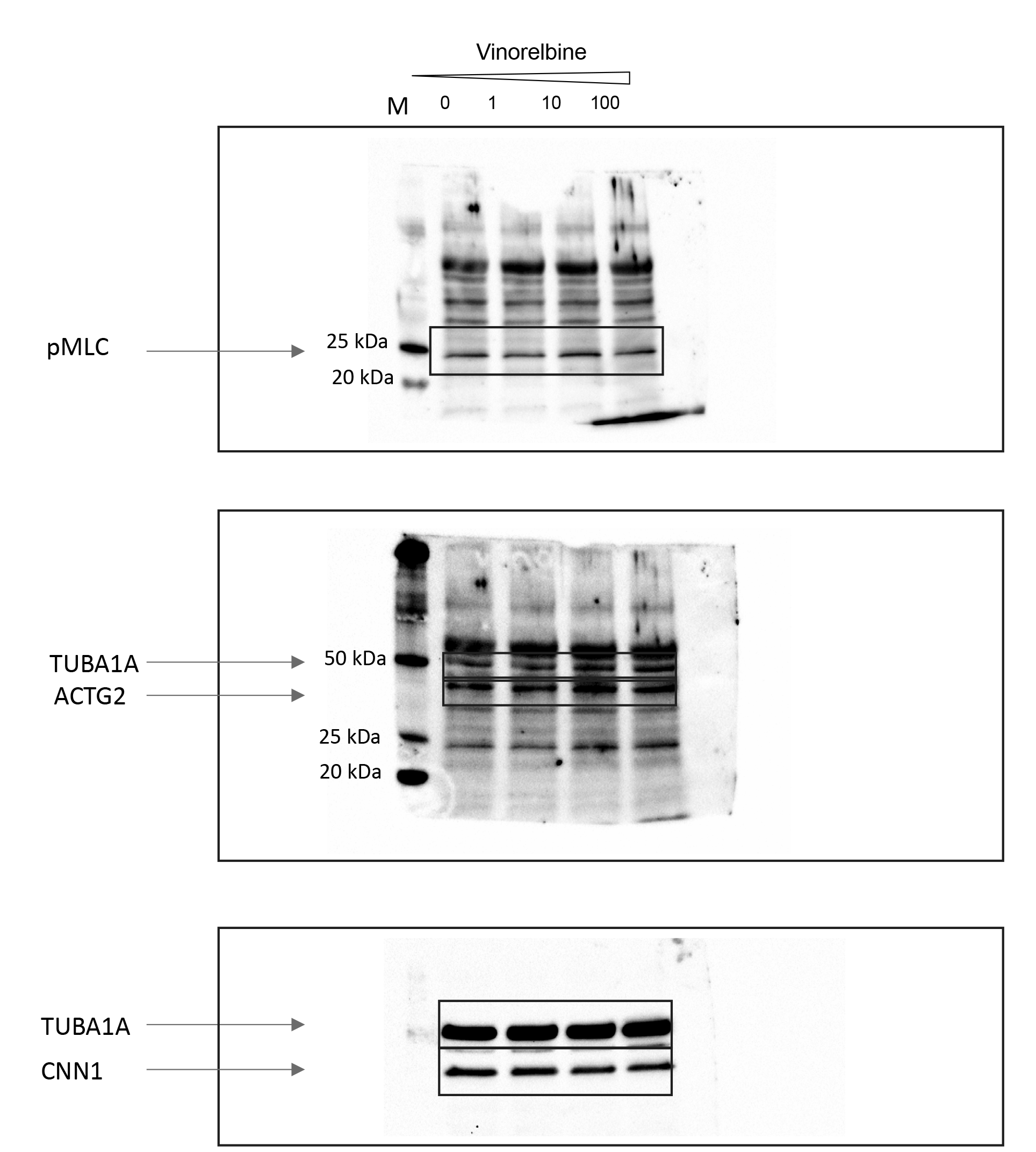

Supplement: Supplementary file 3 — Source data Fig. 1 [file 44321_2025_222_MOESM3_ESM.zip › For EMM submission/Figure 1E/Vinorelbine WB 3 repeat.tif]

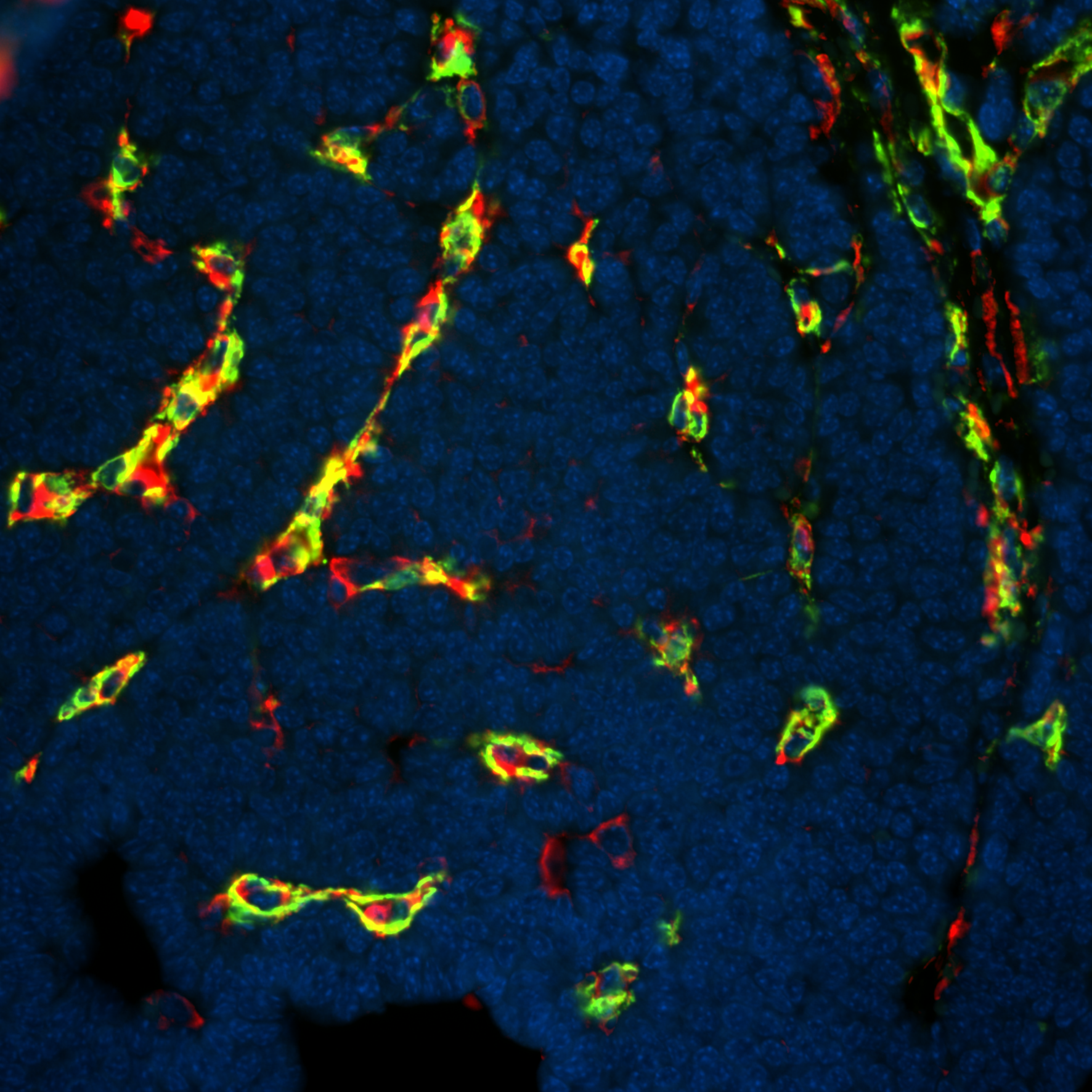

Supplement: Supplementary file 4 — Source data Fig. 2 [file 44321_2025_222_MOESM4_ESM.zip › For EMM submission/Figure 2B/RT5 CA4.tif]

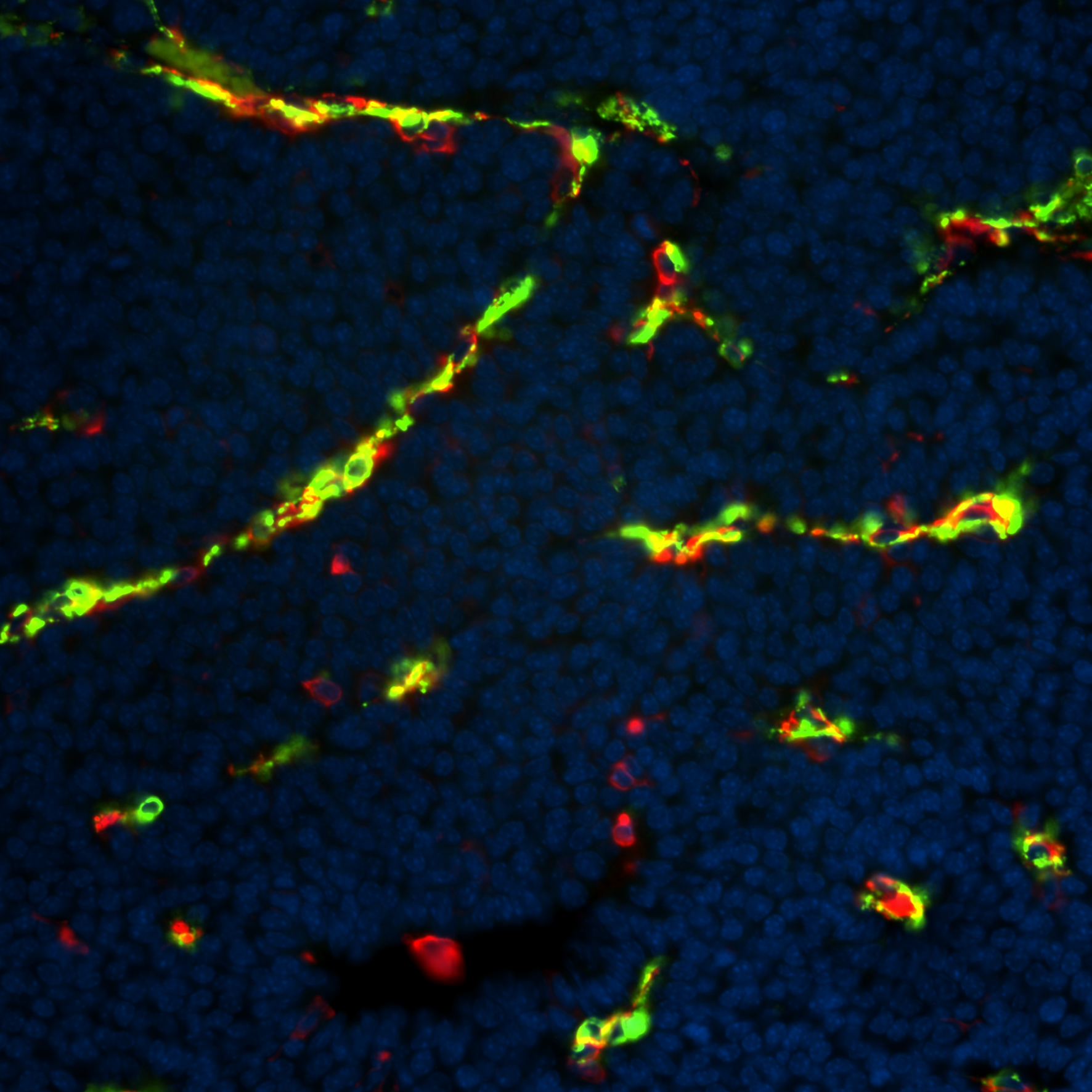

Supplement: Supplementary file 4 — Source data Fig. 2 [file 44321_2025_222_MOESM4_ESM.zip › For EMM submission/Figure 2B/RT5 eribulin.tif]

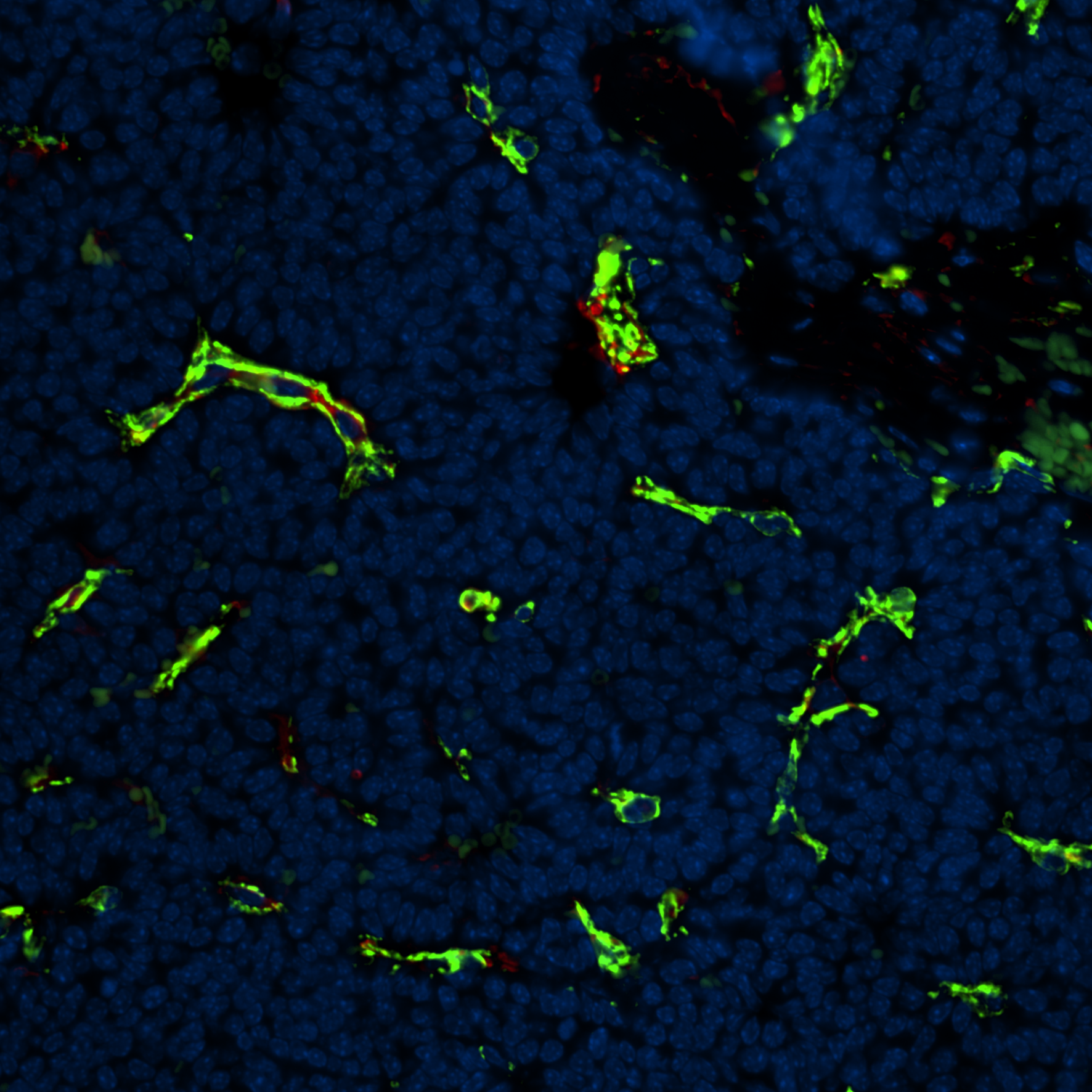

Supplement: Supplementary file 4 — Source data Fig. 2 [file 44321_2025_222_MOESM4_ESM.zip › For EMM submission/Figure 2B/RT5 paclitaxel.tif]

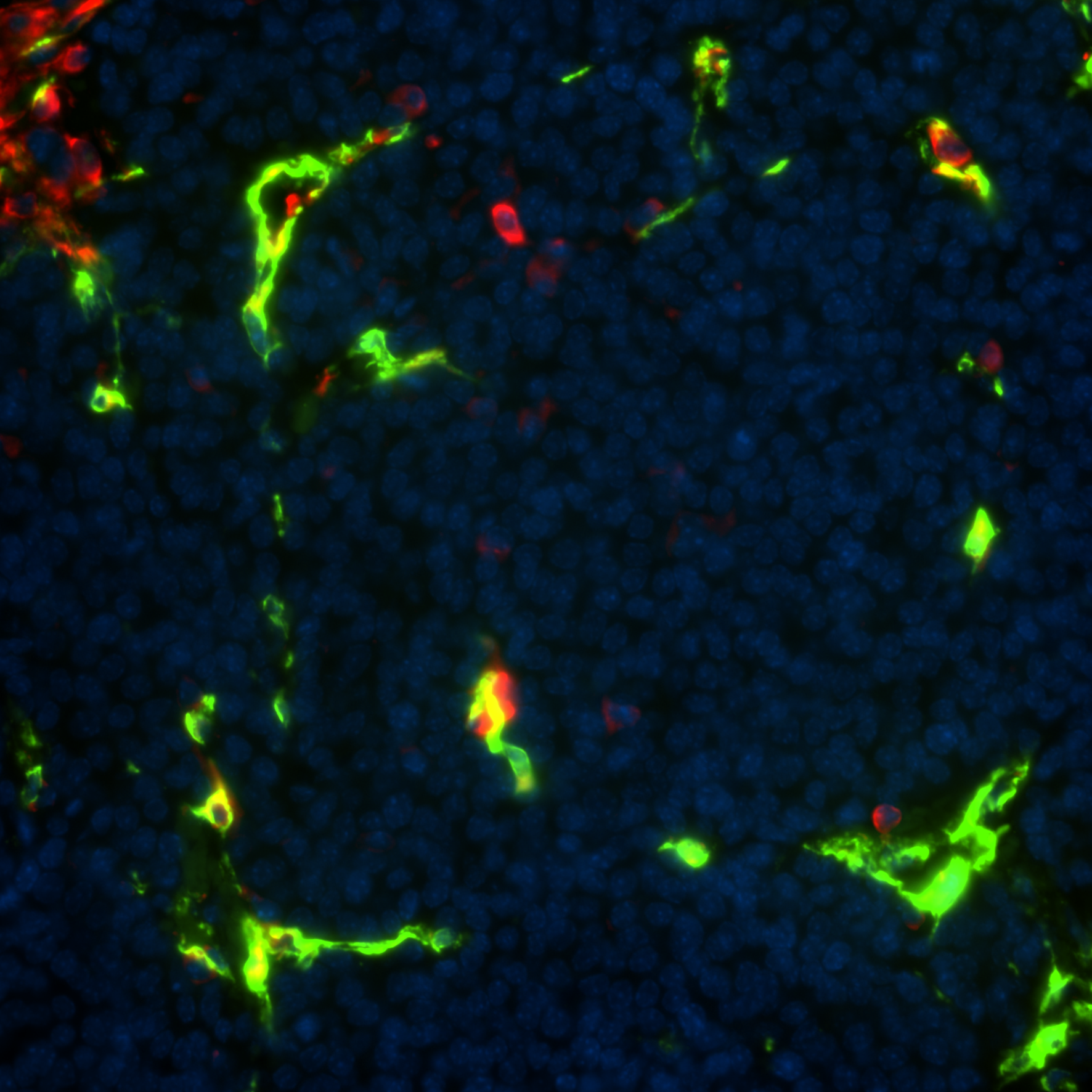

Supplement: Supplementary file 4 — Source data Fig. 2 [file 44321_2025_222_MOESM4_ESM.zip › For EMM submission/Figure 2B/RT5 untreated.tif]

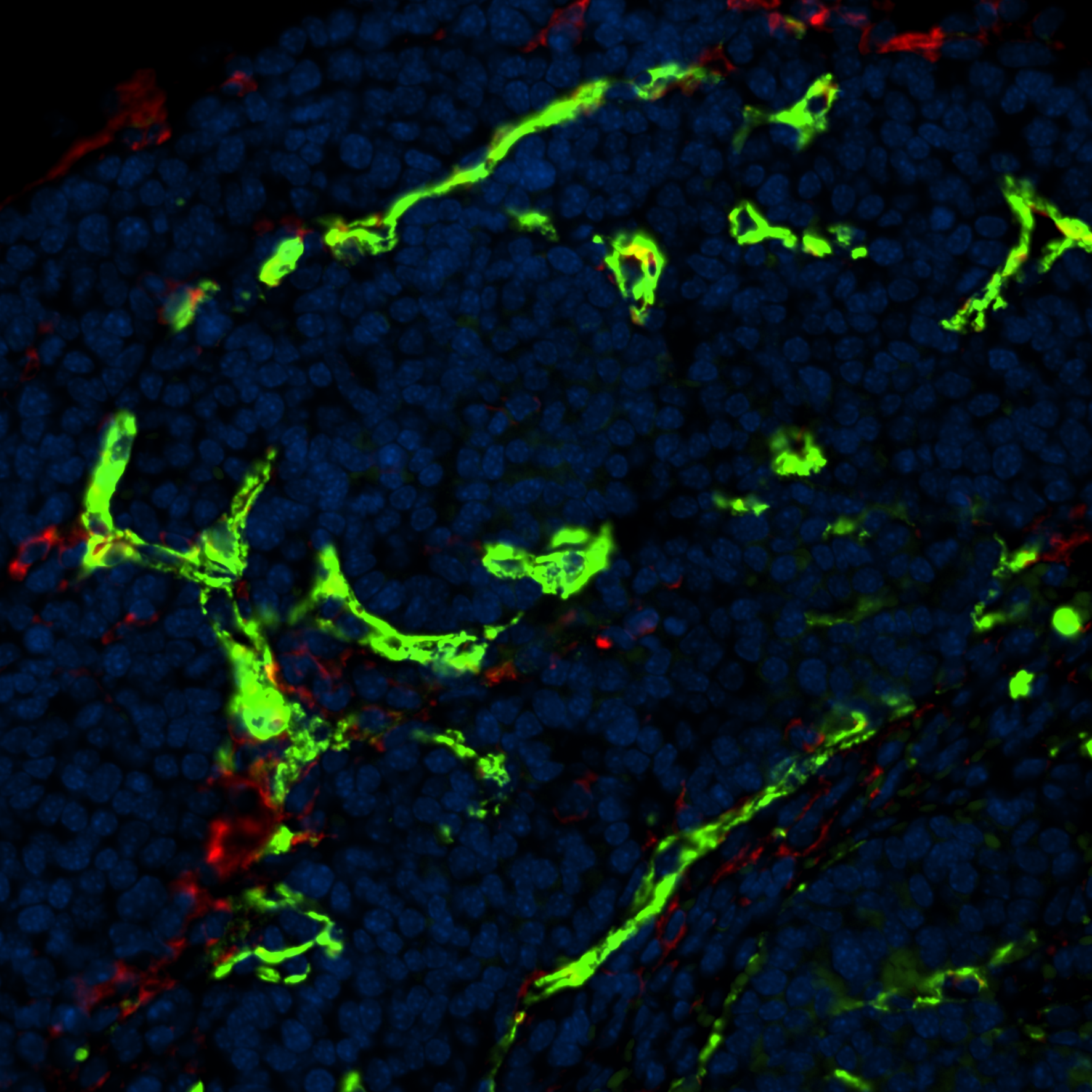

Supplement: Supplementary file 4 — Source data Fig. 2 [file 44321_2025_222_MOESM4_ESM.zip › For EMM submission/Figure 2B/RT5 vinorelbine.tif]

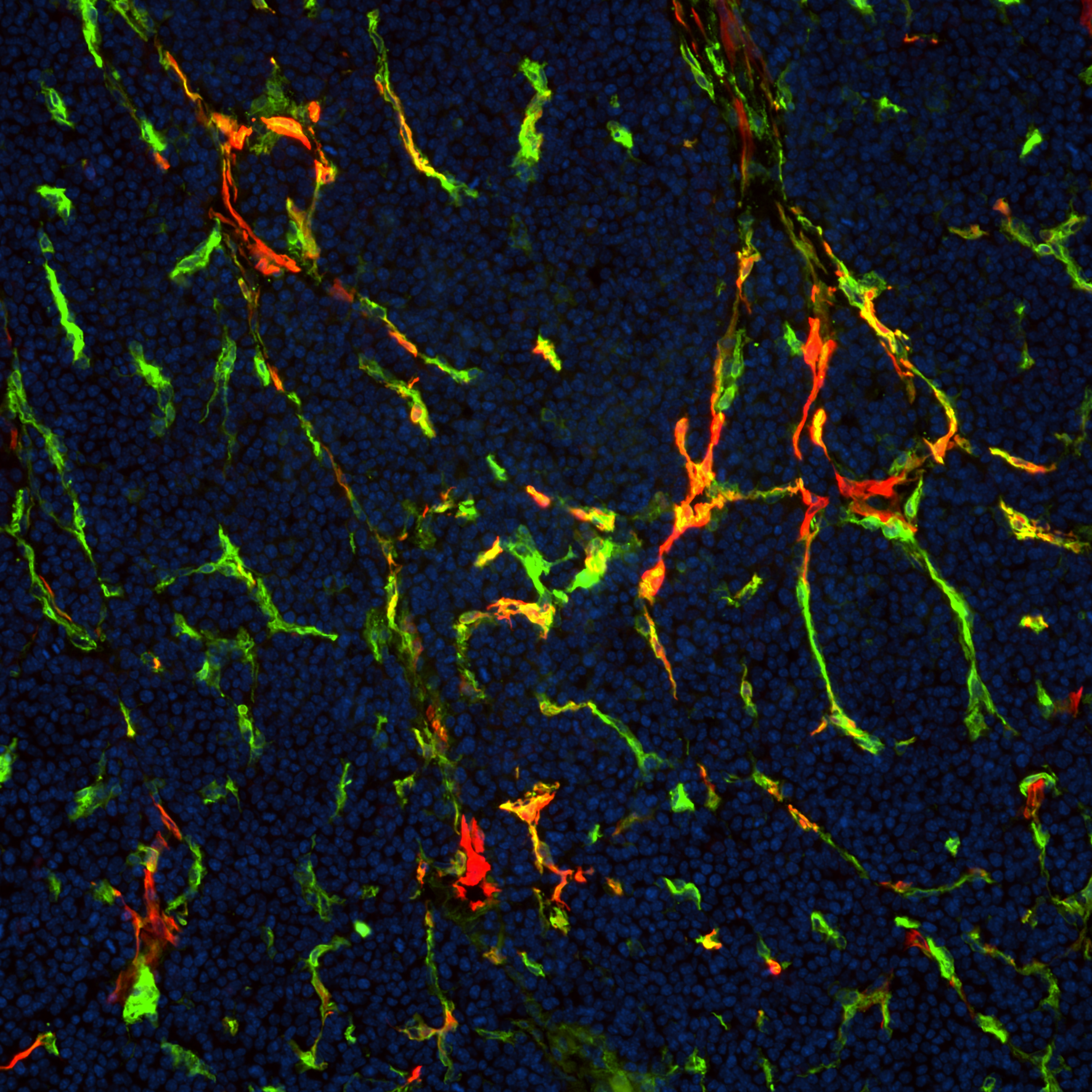

Supplement: Supplementary file 4 — Source data Fig. 2 [file 44321_2025_222_MOESM4_ESM.zip › For EMM submission/Figure 2C/RT5 CA4.tif]

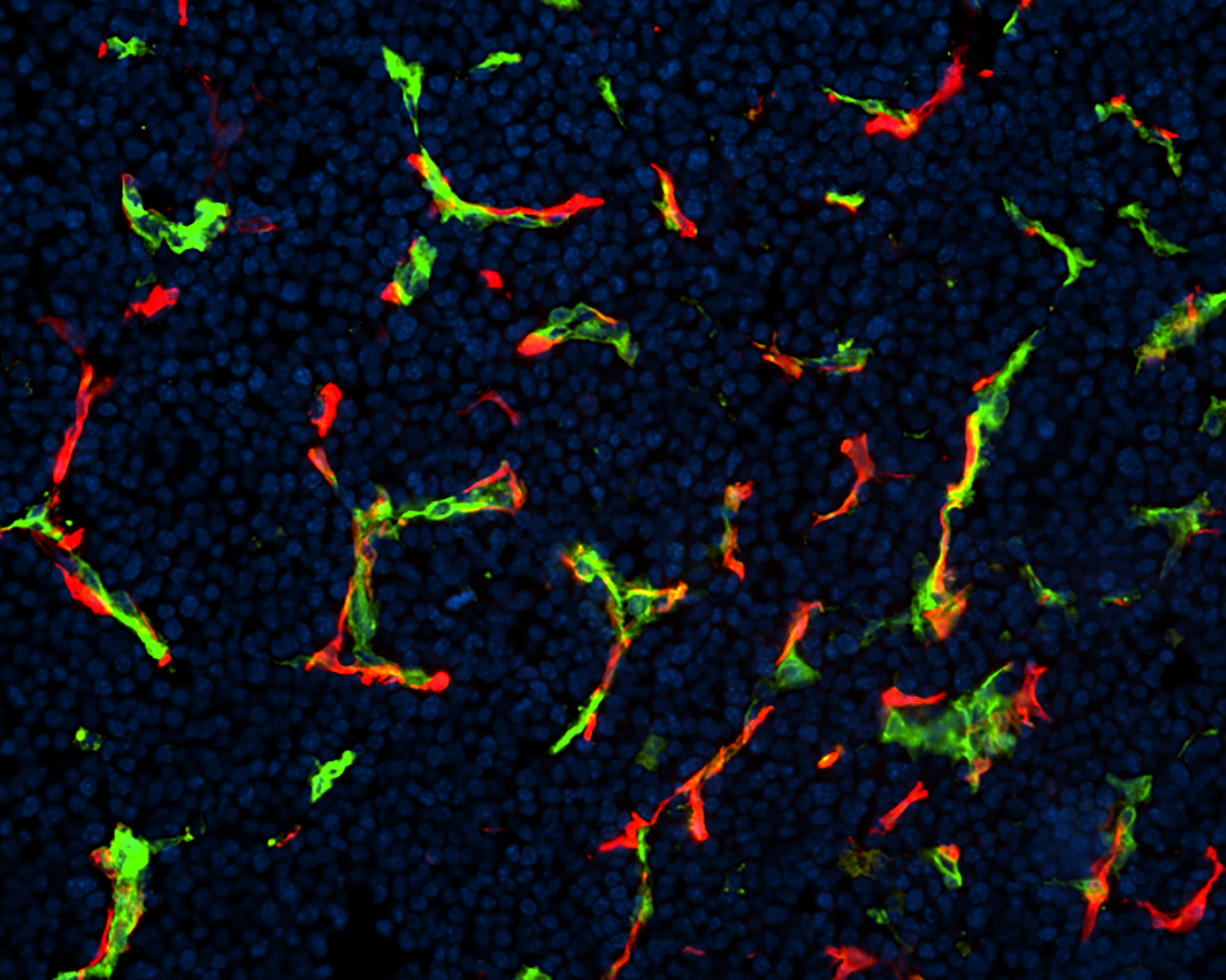

Supplement: Supplementary file 4 — Source data Fig. 2 [file 44321_2025_222_MOESM4_ESM.zip › For EMM submission/Figure 2C/RT5 Eribulin.tif]

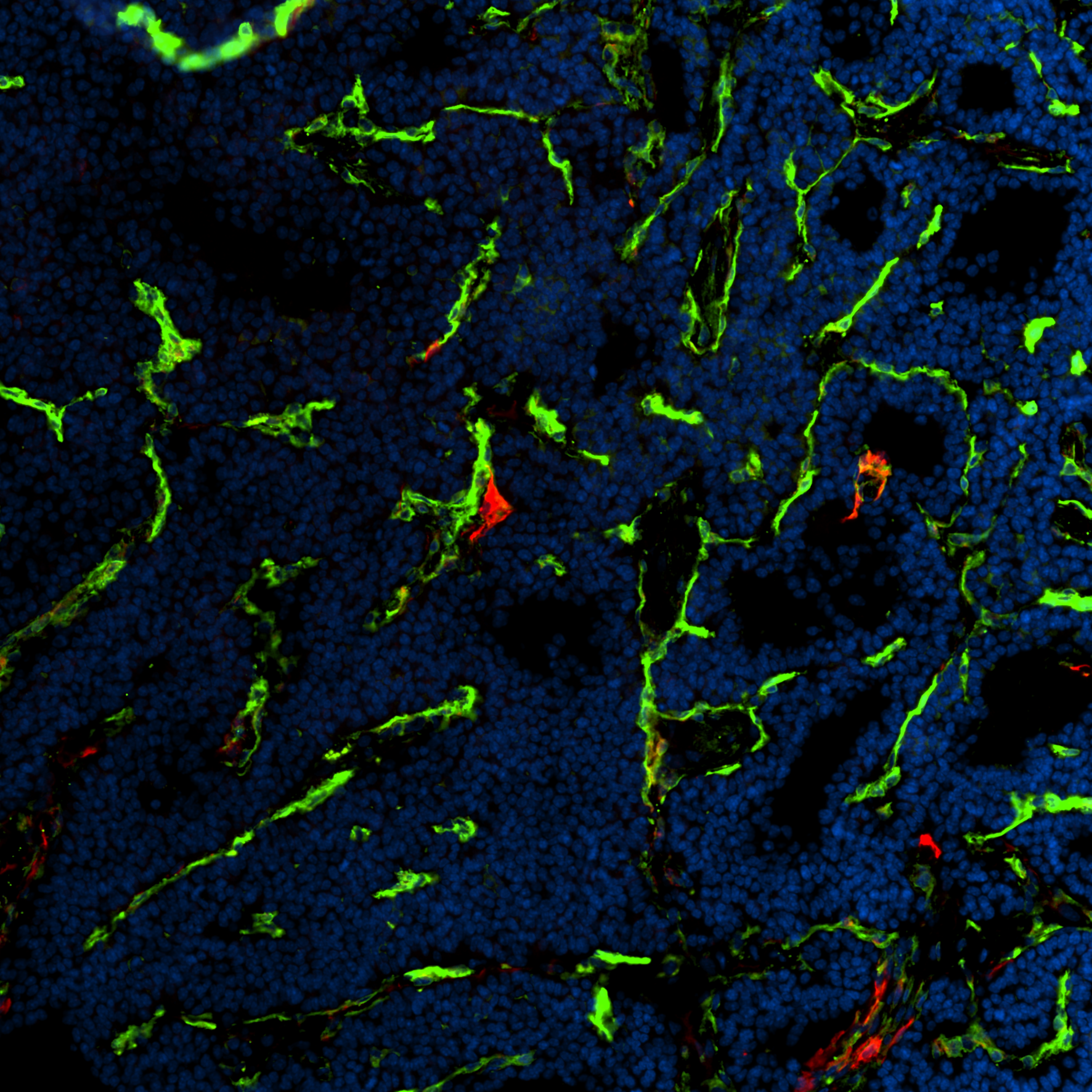

Supplement: Supplementary file 4 — Source data Fig. 2 [file 44321_2025_222_MOESM4_ESM.zip › For EMM submission/Figure 2C/RT5 Paclitaxel.tif]

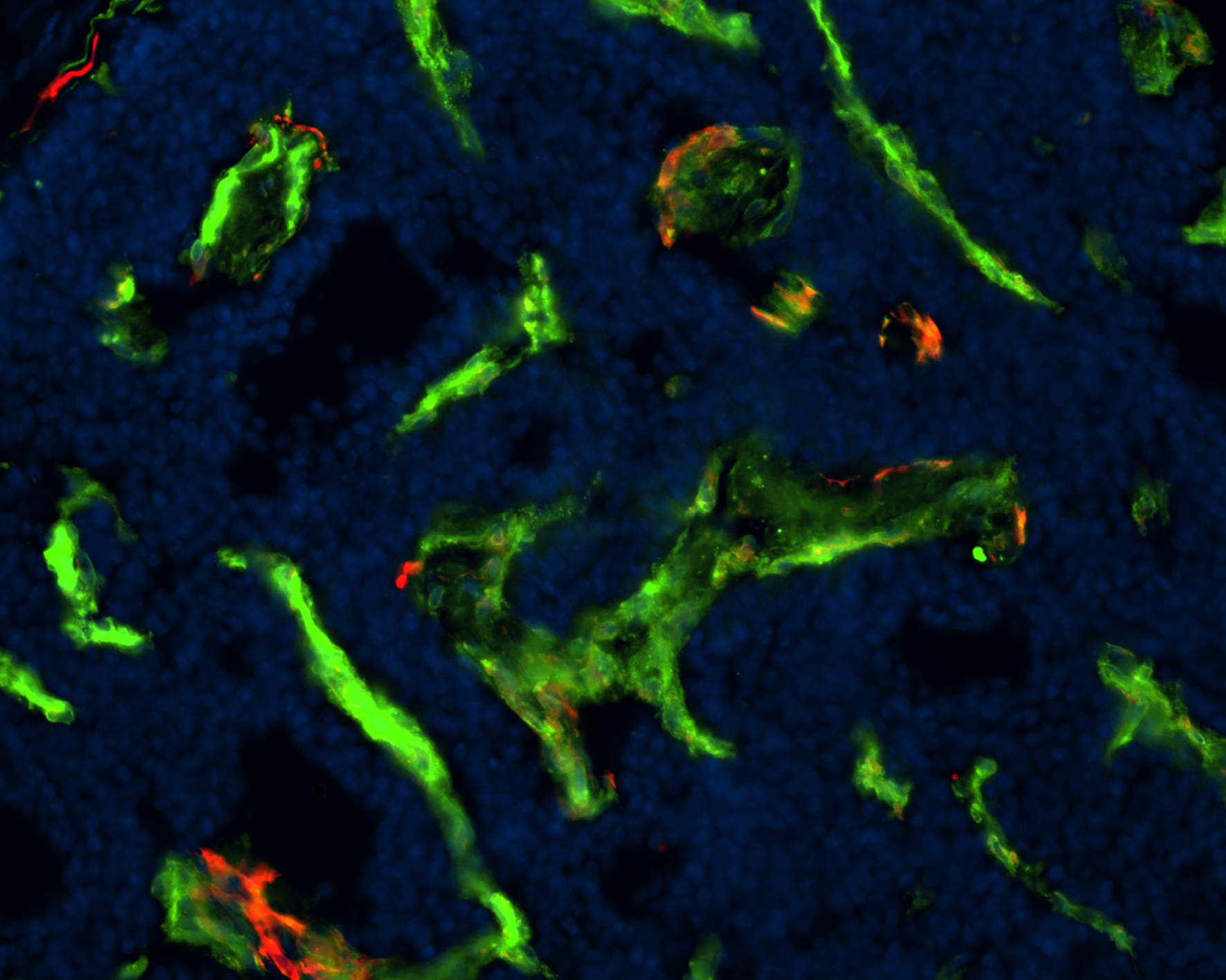

Supplement: Supplementary file 4 — Source data Fig. 2 [file 44321_2025_222_MOESM4_ESM.zip › For EMM submission/Figure 2C/RT5 untreated.tif]

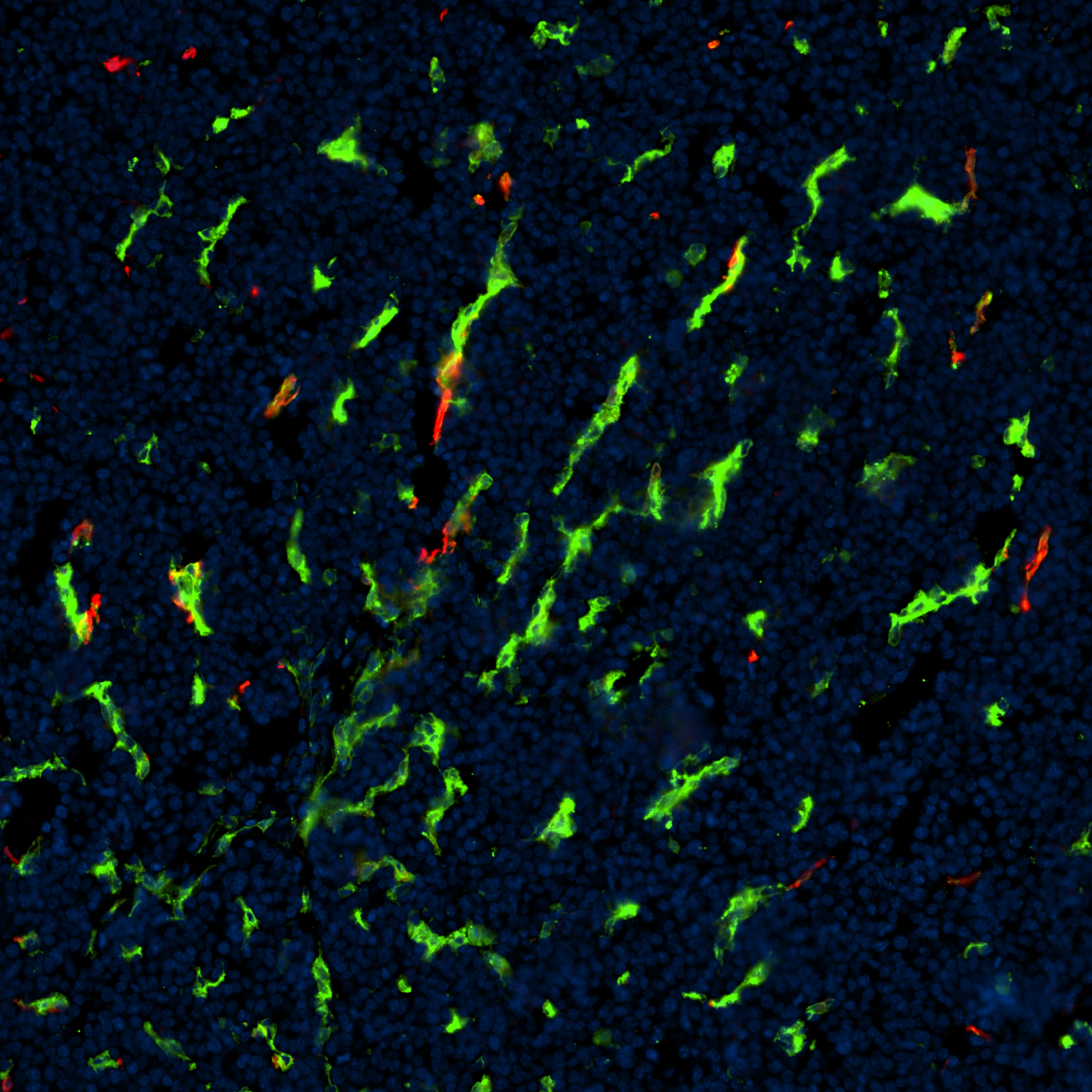

Supplement: Supplementary file 4 — Source data Fig. 2 [file 44321_2025_222_MOESM4_ESM.zip › For EMM submission/Figure 2C/RT5 Vinorelbine.tif]

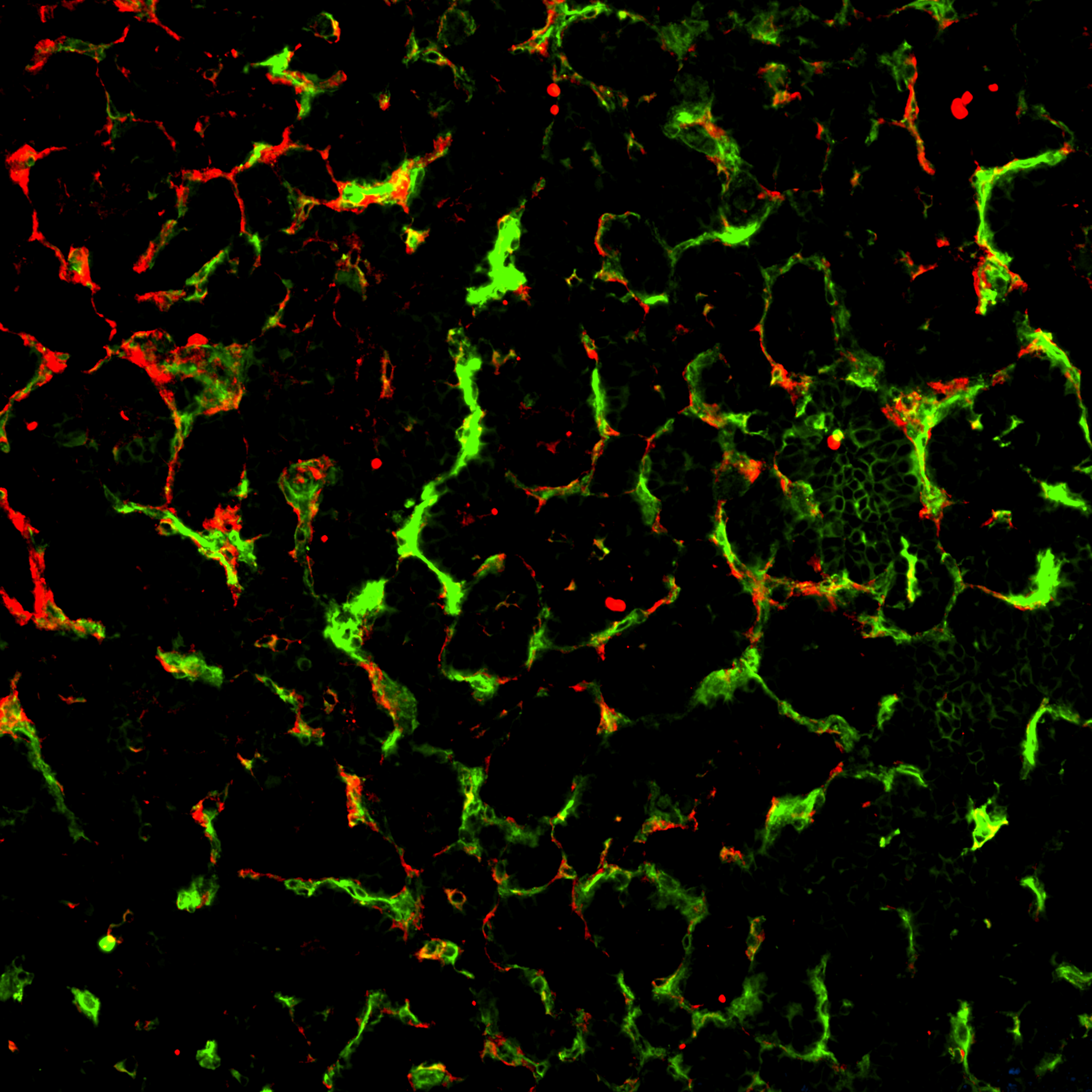

Supplement: Supplementary file 4 — Source data Fig. 2 [file 44321_2025_222_MOESM4_ESM.zip › For EMM submission/Figure 2D/RT5 CA4.tif]

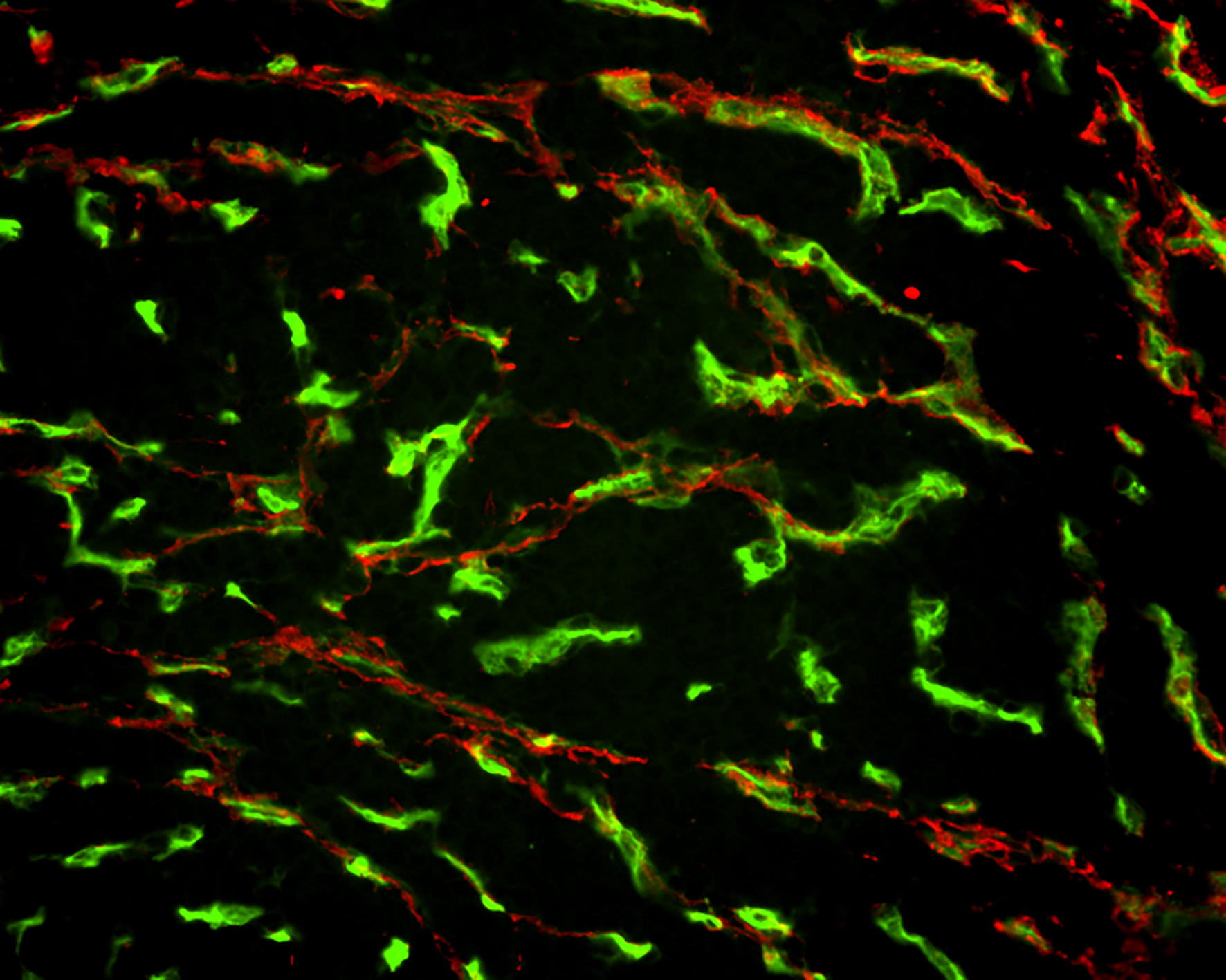

Supplement: Supplementary file 4 — Source data Fig. 2 [file 44321_2025_222_MOESM4_ESM.zip › For EMM submission/Figure 2D/RT5 Eribulin.tif]

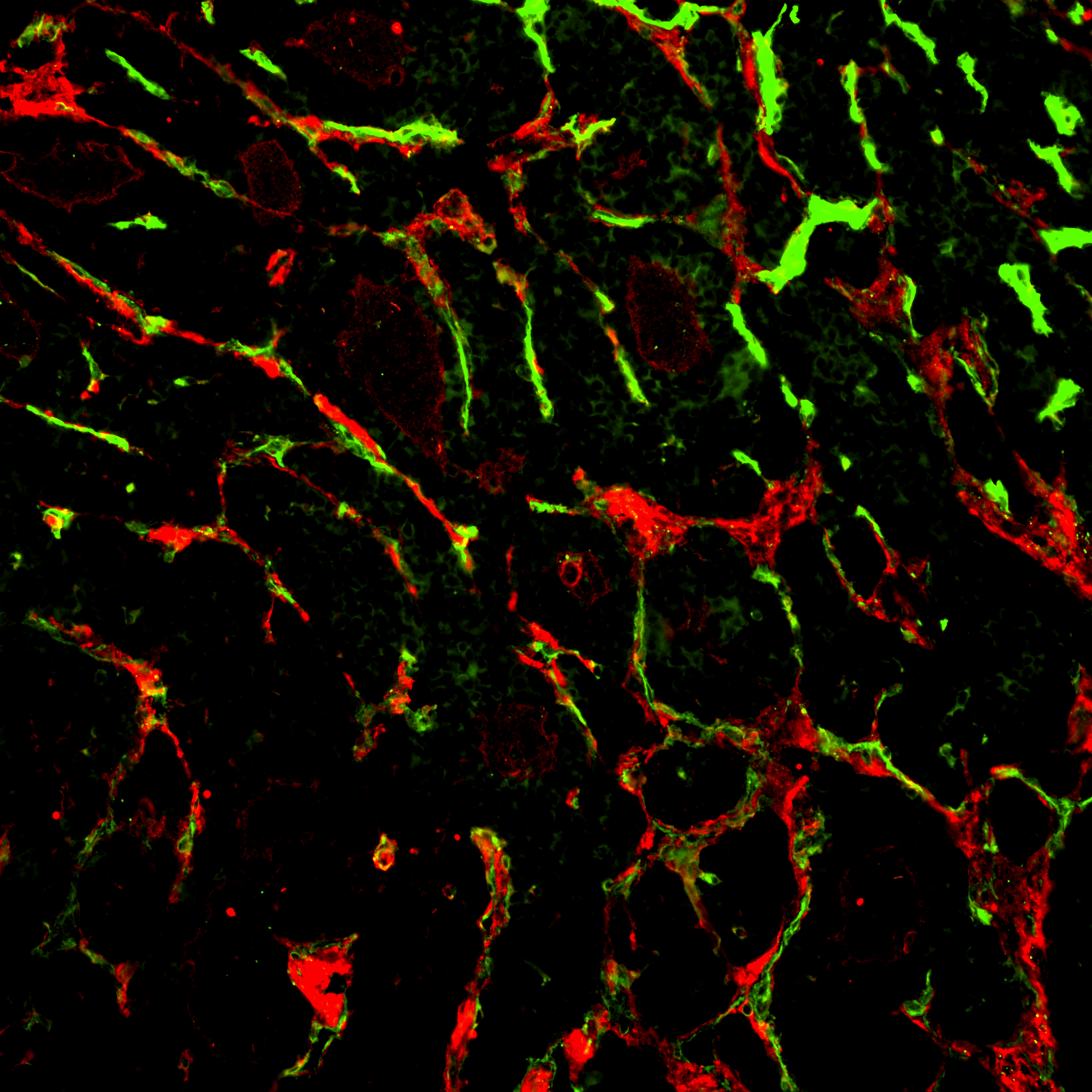

Supplement: Supplementary file 4 — Source data Fig. 2 [file 44321_2025_222_MOESM4_ESM.zip › For EMM submission/Figure 2D/RT5 Paclitaxel.tif]

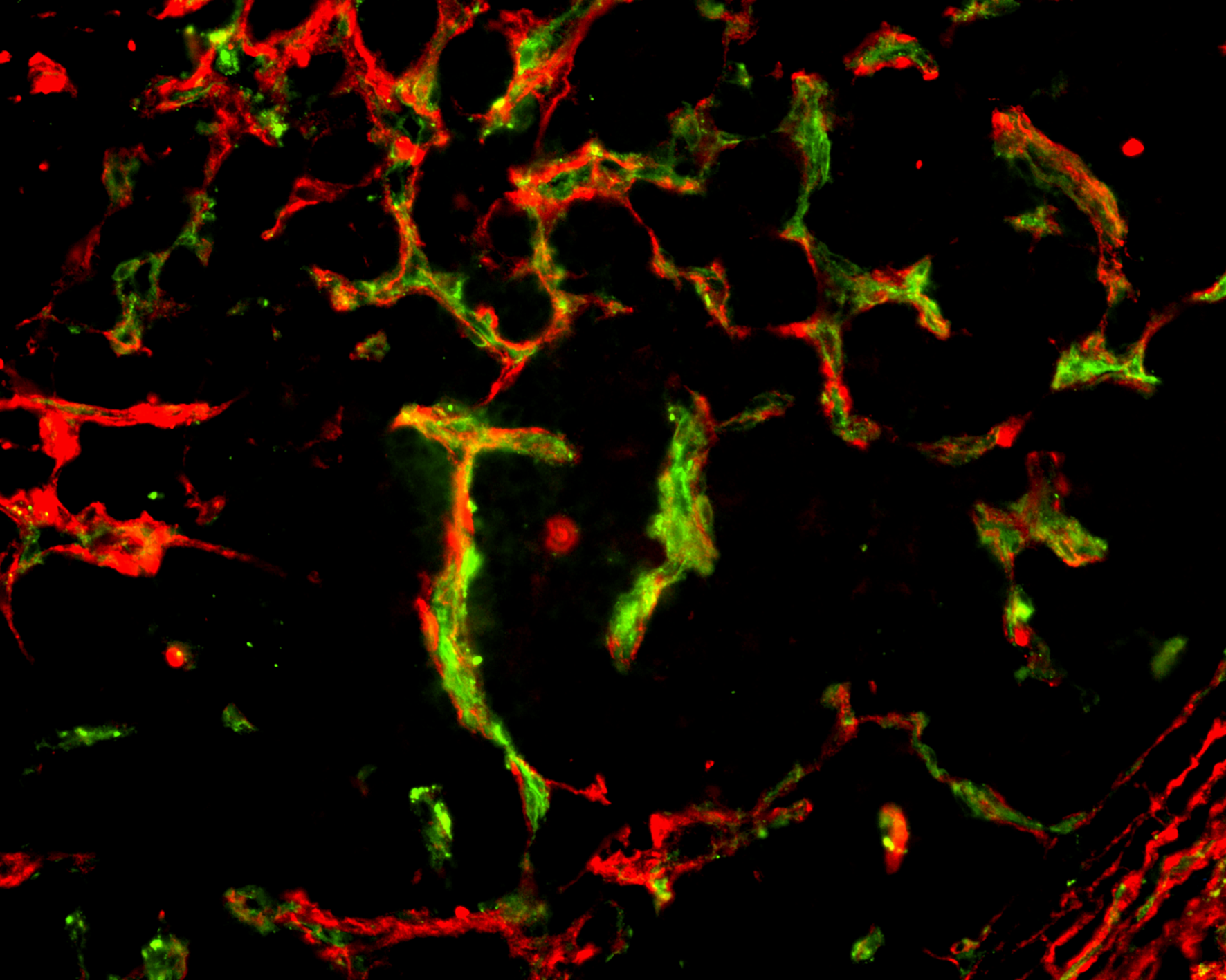

Supplement: Supplementary file 4 — Source data Fig. 2 [file 44321_2025_222_MOESM4_ESM.zip › For EMM submission/Figure 2D/RT5 untreated.tif]

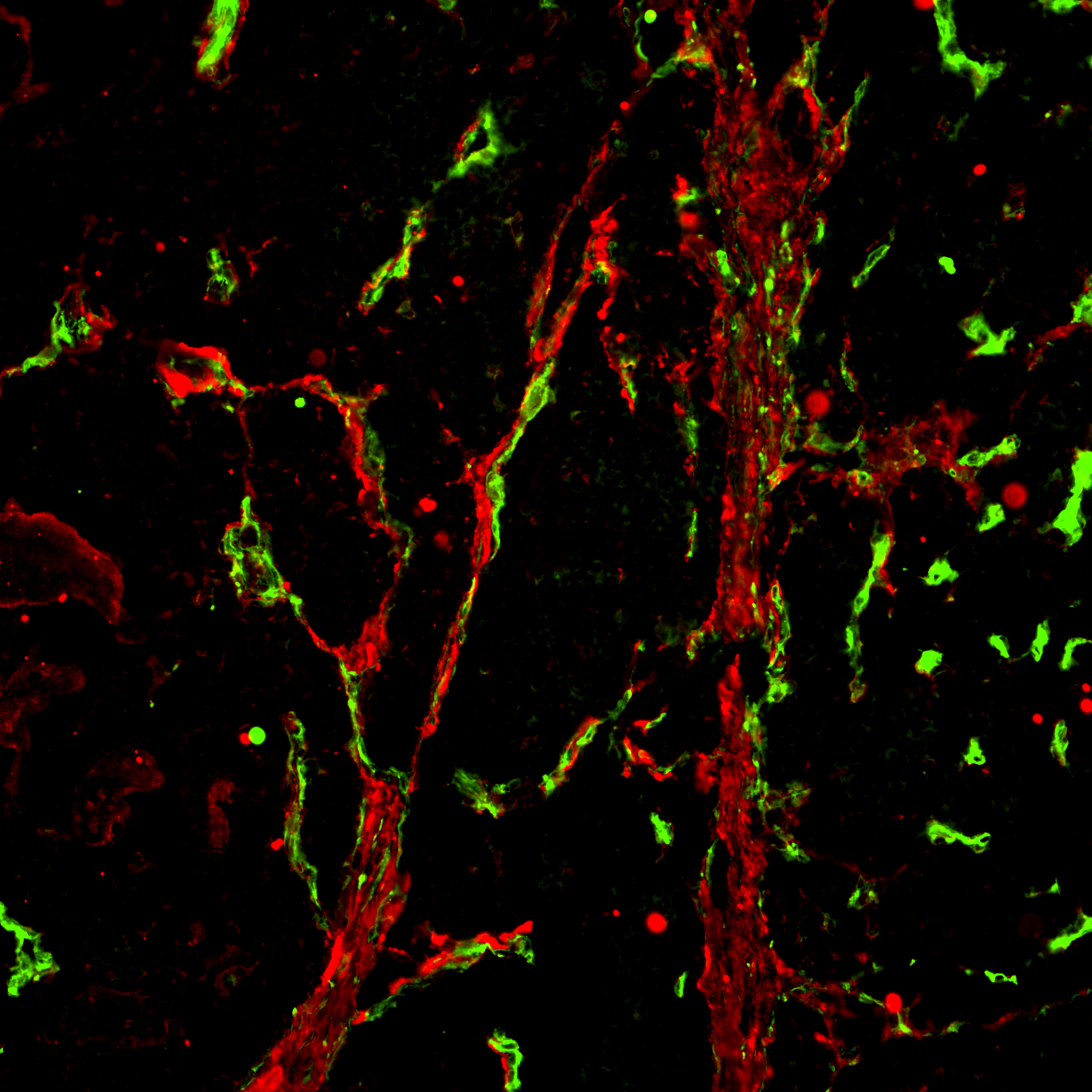

Supplement: Supplementary file 4 — Source data Fig. 2 [file 44321_2025_222_MOESM4_ESM.zip › For EMM submission/Figure 2D/RT5 Vinorelbine.tif]

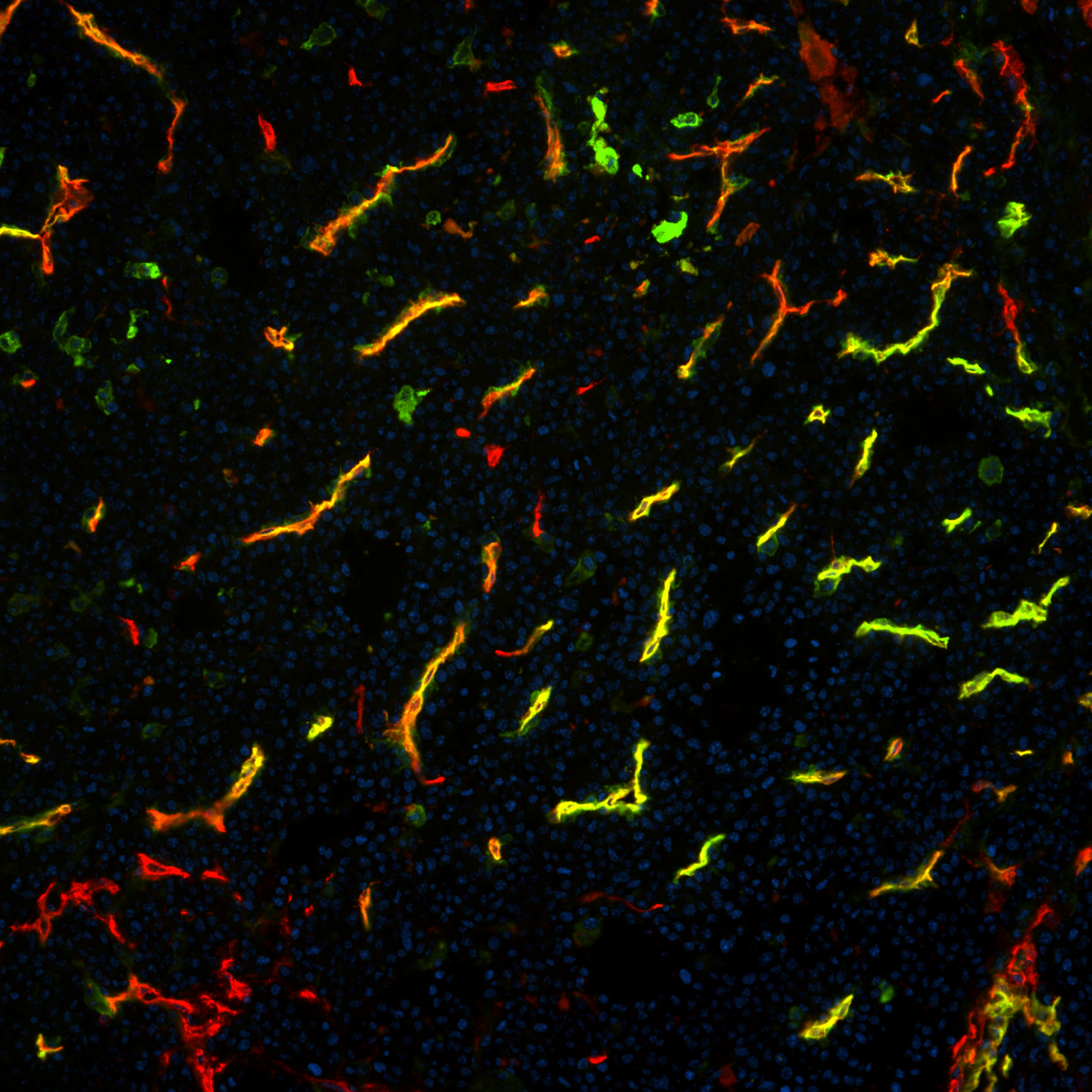

Supplement: Supplementary file 4 — Source data Fig. 2 [file 44321_2025_222_MOESM4_ESM.zip › For EMM submission/Figure 2E/RT5 CA4.tif]

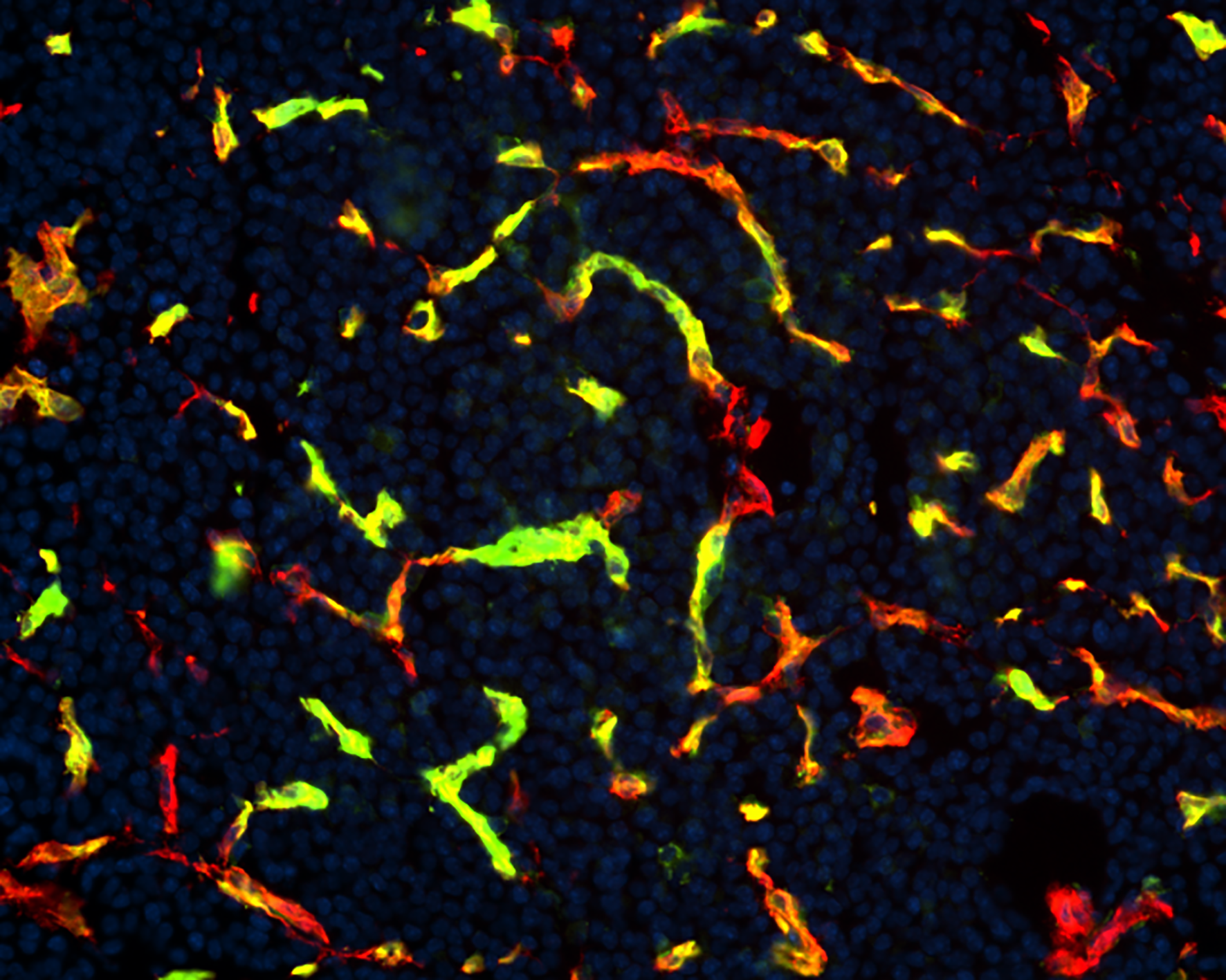

Supplement: Supplementary file 4 — Source data Fig. 2 [file 44321_2025_222_MOESM4_ESM.zip › For EMM submission/Figure 2E/RT5 Eribulin.tif]

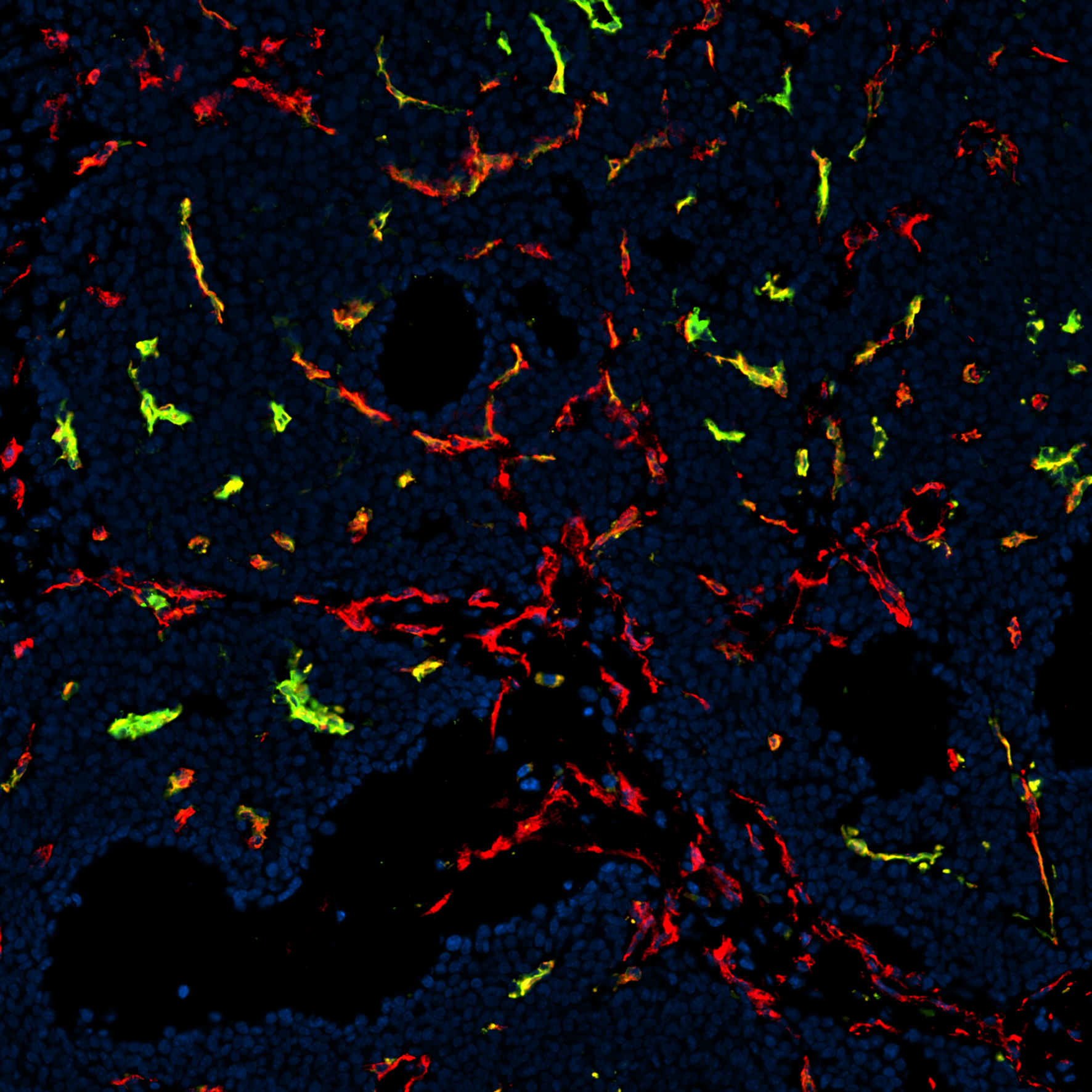

Supplement: Supplementary file 4 — Source data Fig. 2 [file 44321_2025_222_MOESM4_ESM.zip › For EMM submission/Figure 2E/RT5 Paclitaxel.tif]

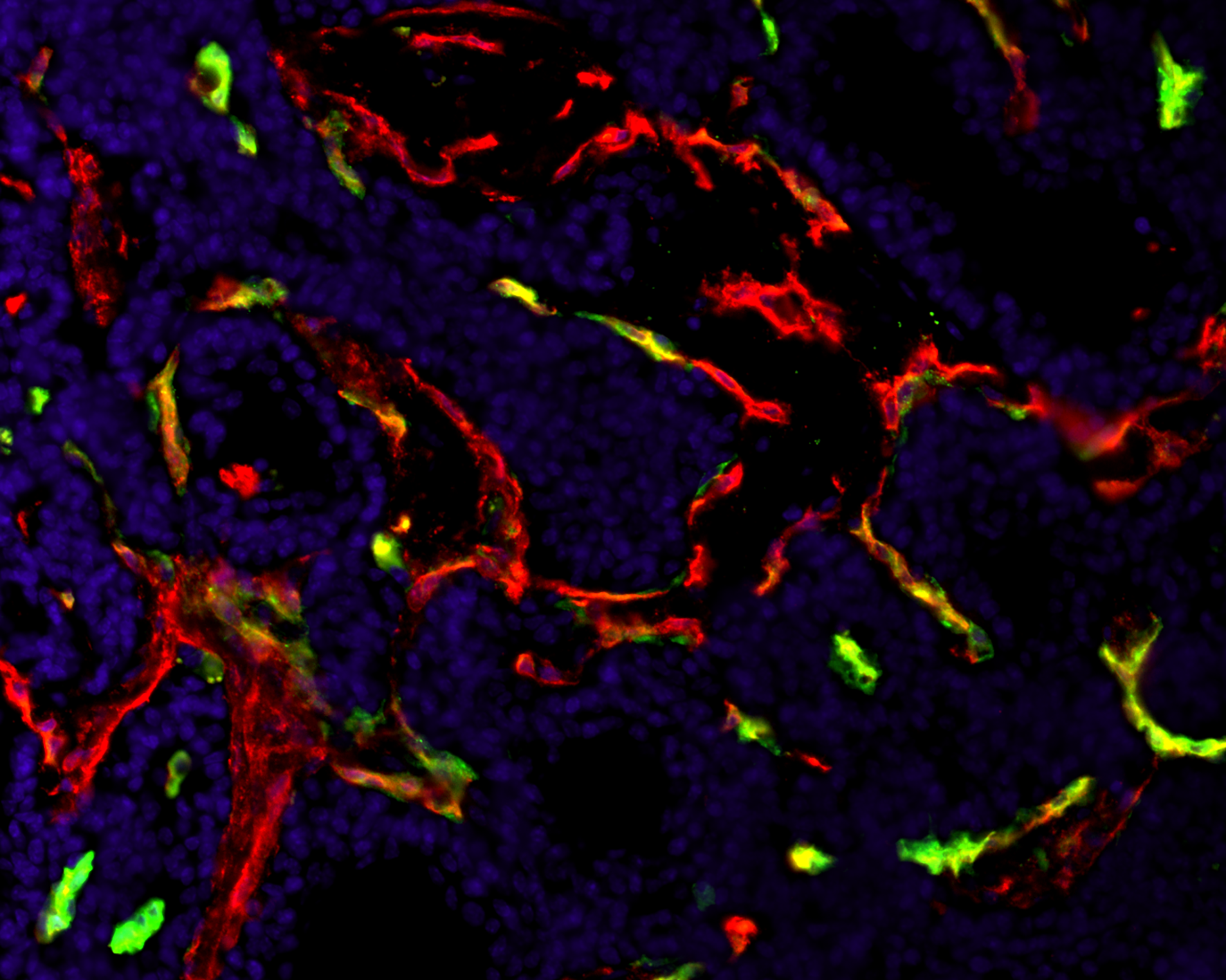

Supplement: Supplementary file 4 — Source data Fig. 2 [file 44321_2025_222_MOESM4_ESM.zip › For EMM submission/Figure 2E/RT5 untreated.tif]

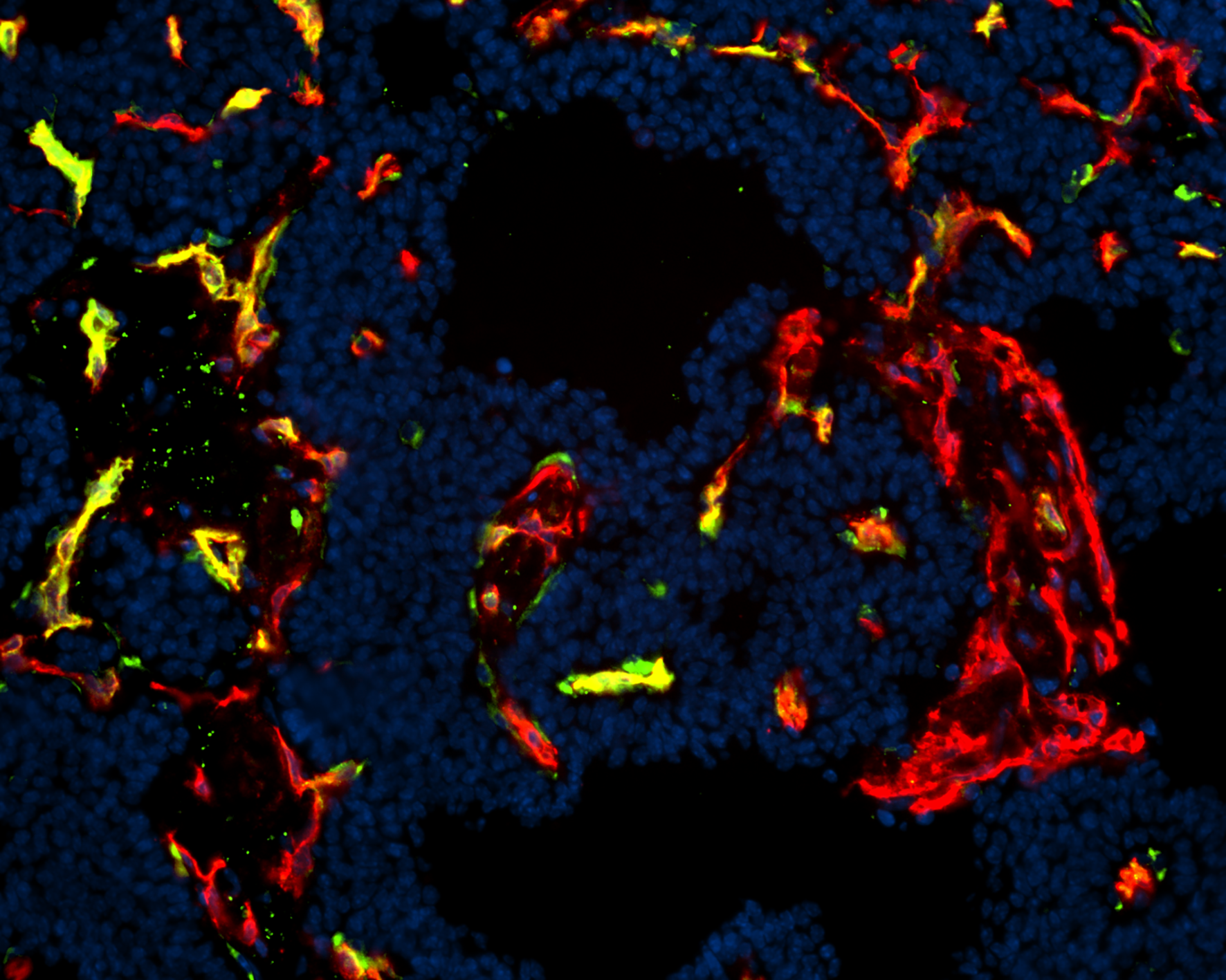

Supplement: Supplementary file 4 — Source data Fig. 2 [file 44321_2025_222_MOESM4_ESM.zip › For EMM submission/Figure 2E/RT5 Vinorelbine.tif]

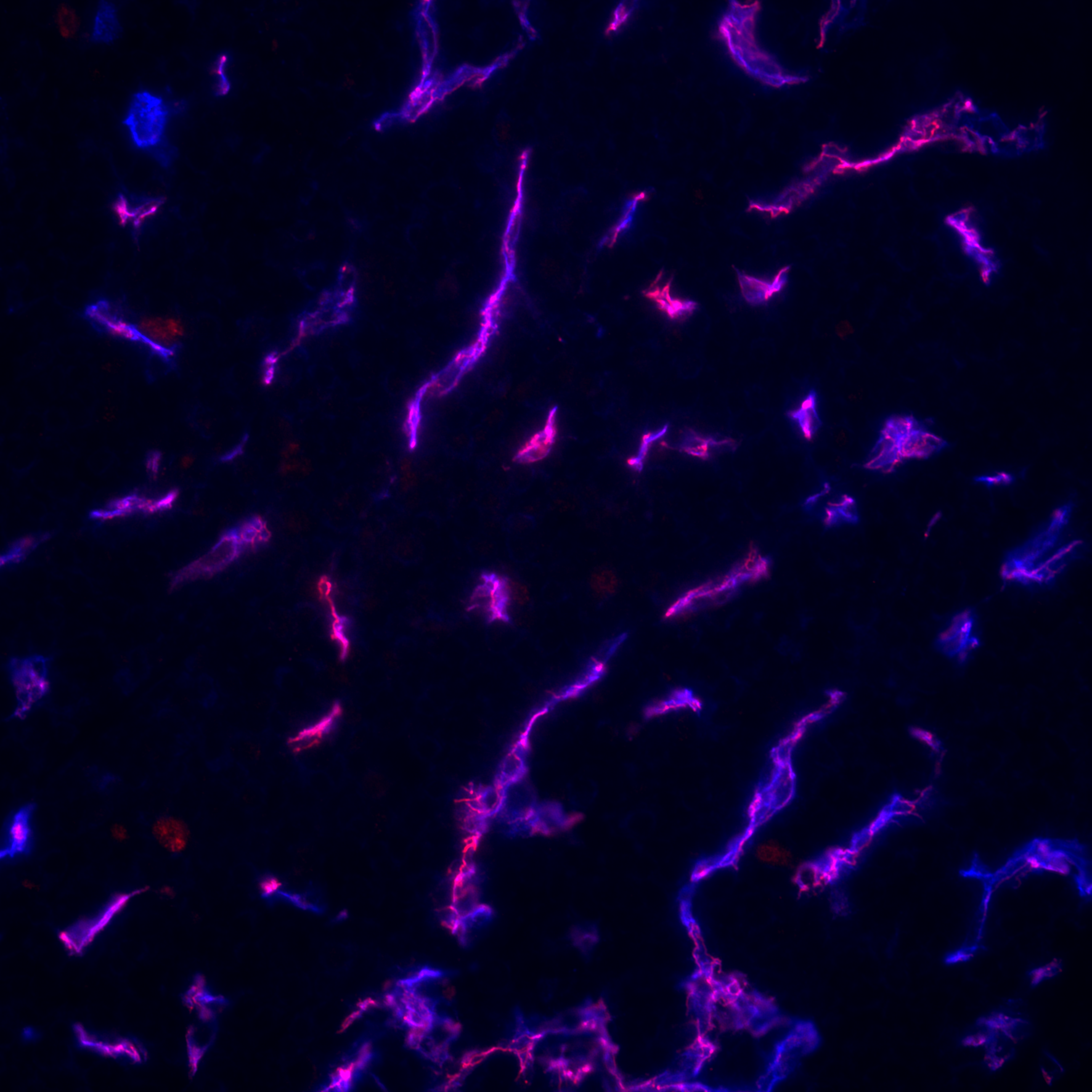

Supplement: Supplementary file 4 — Source data Fig. 2 [file 44321_2025_222_MOESM4_ESM.zip › For EMM submission/Figure 2F/RT5 CA4.tif]

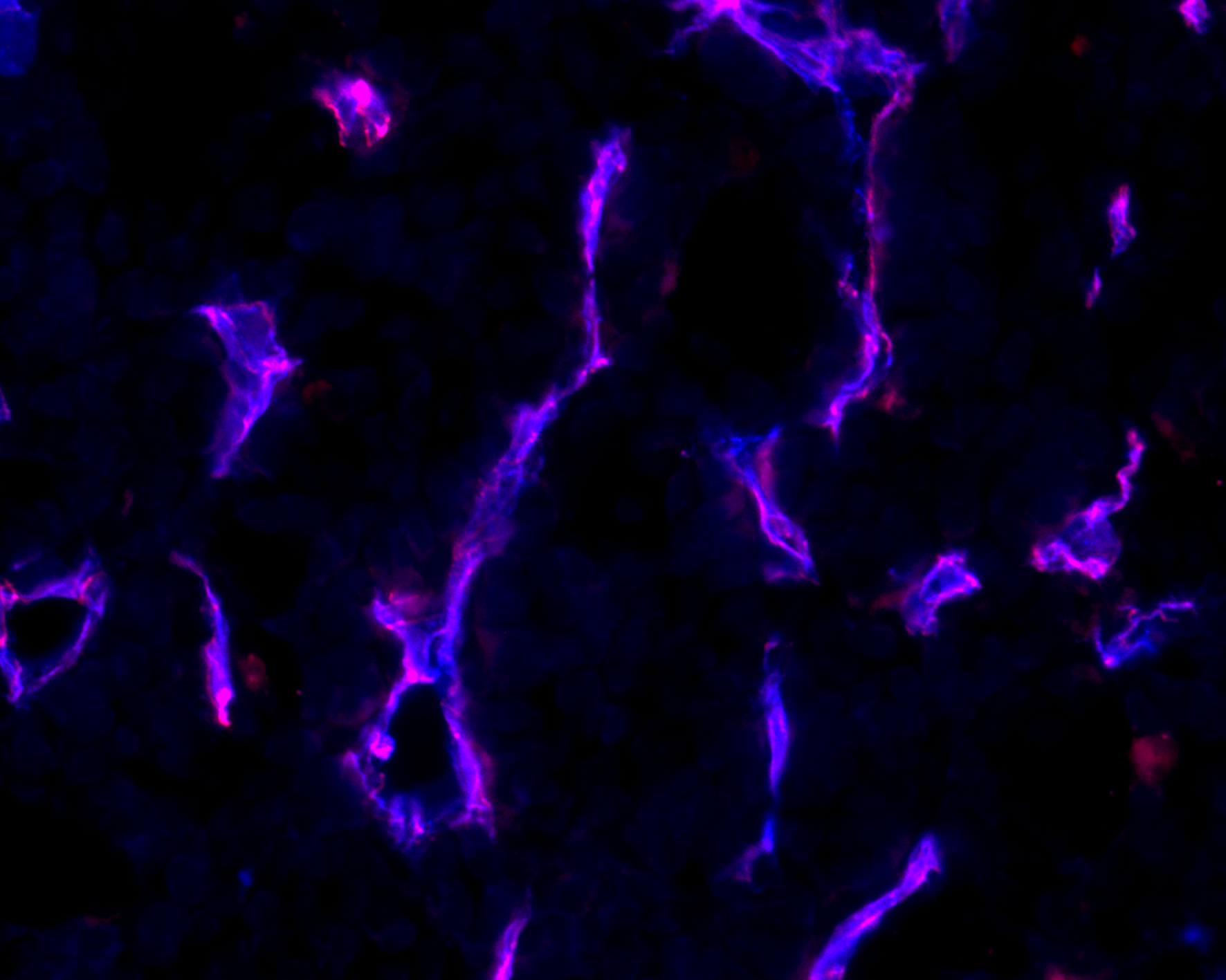

Supplement: Supplementary file 4 — Source data Fig. 2 [file 44321_2025_222_MOESM4_ESM.zip › For EMM submission/Figure 2F/RT5 Eribulin.tif]

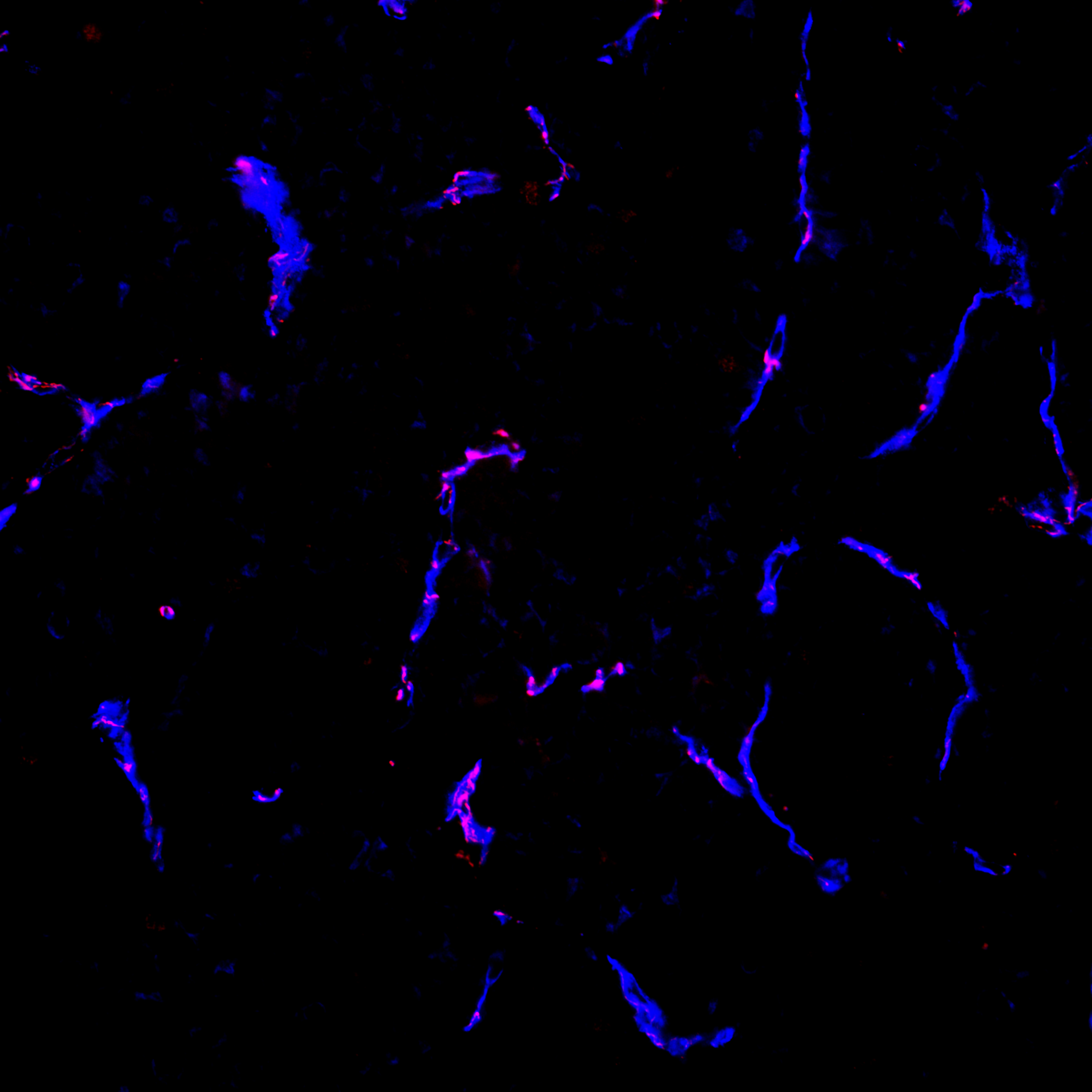

Supplement: Supplementary file 4 — Source data Fig. 2 [file 44321_2025_222_MOESM4_ESM.zip › For EMM submission/Figure 2F/RT5 Paclitaxel.tif]

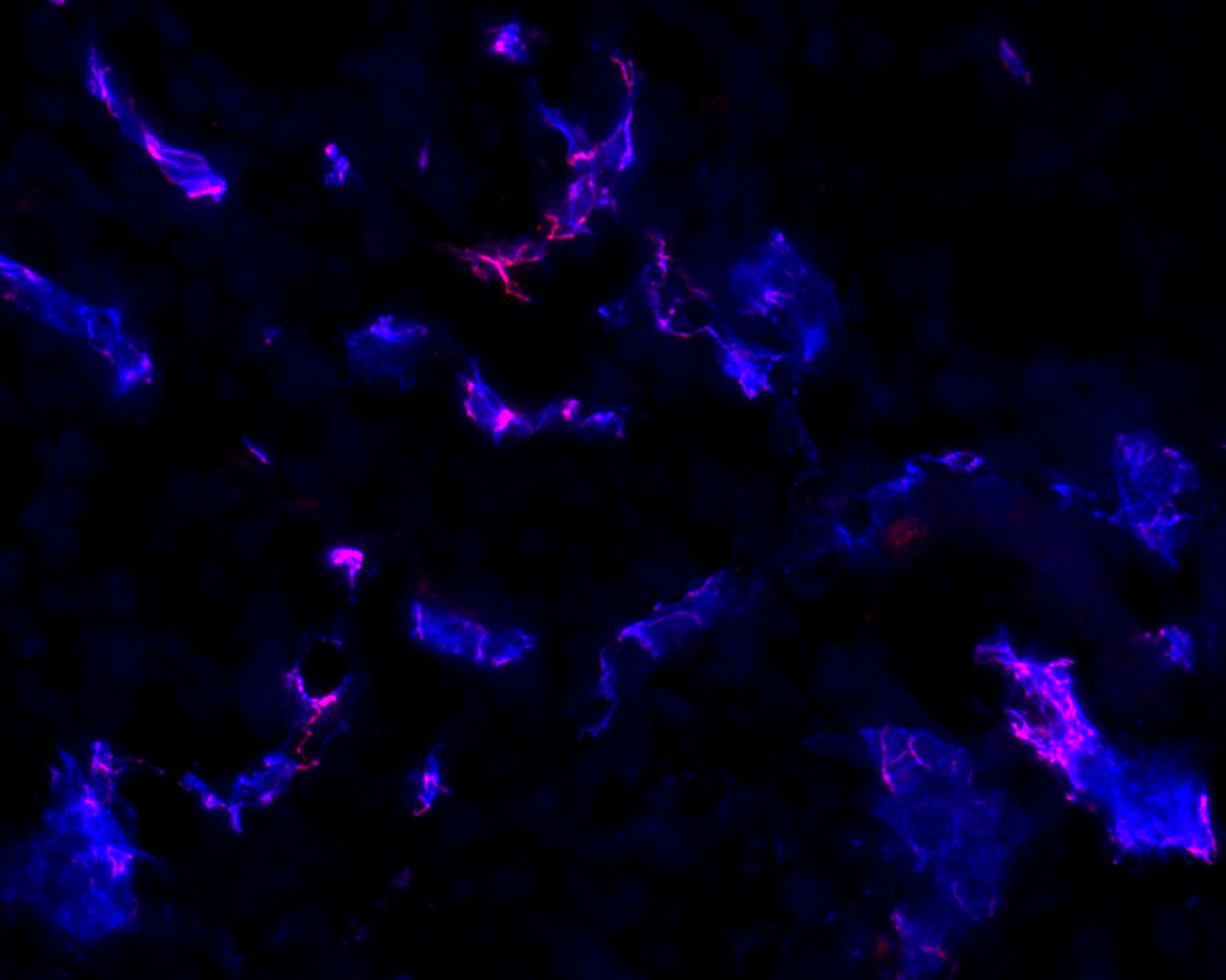

Supplement: Supplementary file 4 — Source data Fig. 2 [file 44321_2025_222_MOESM4_ESM.zip › For EMM submission/Figure 2F/RT5 untreated.tif]

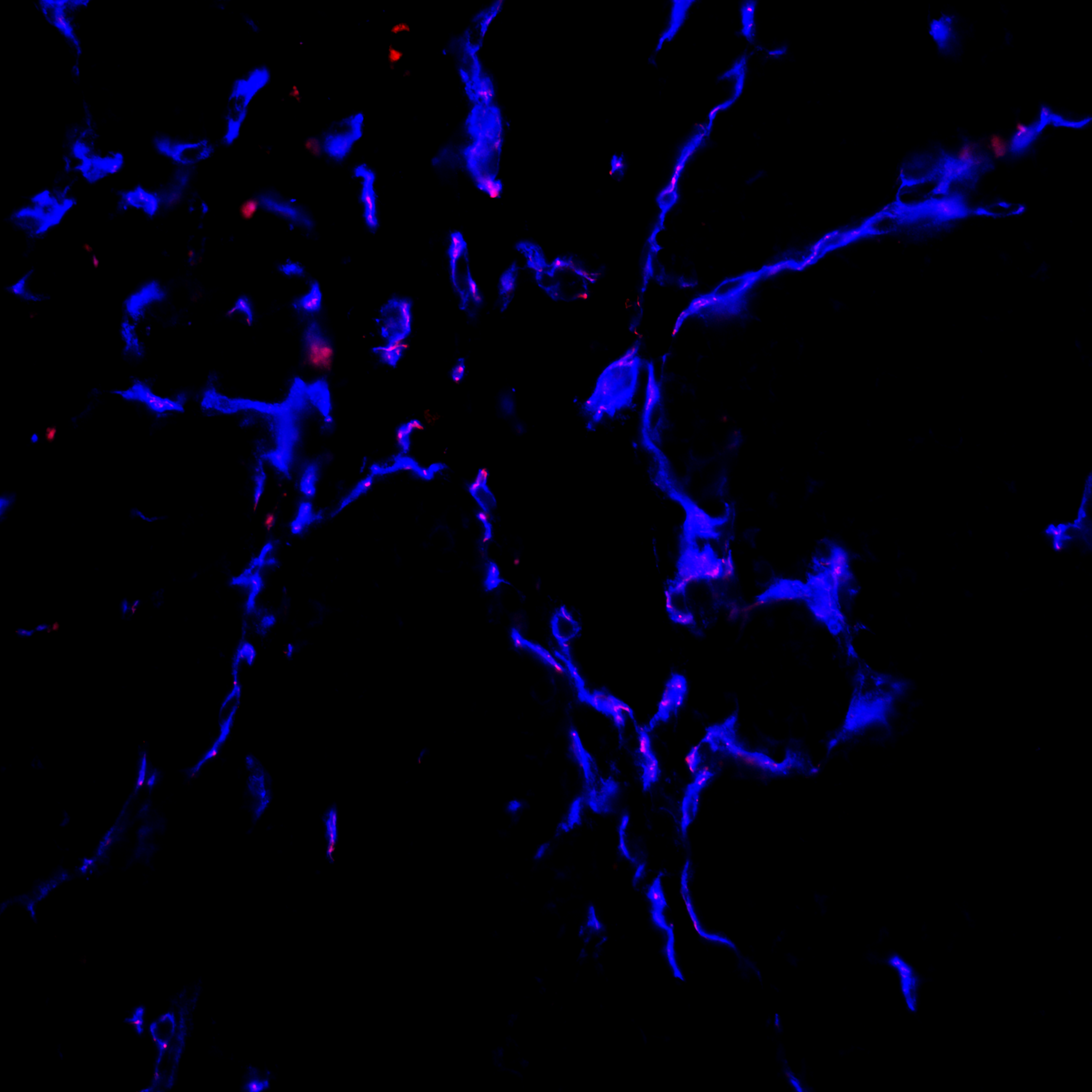

Supplement: Supplementary file 4 — Source data Fig. 2 [file 44321_2025_222_MOESM4_ESM.zip › For EMM submission/Figure 2F/RT5 Vinorelbine.tif]

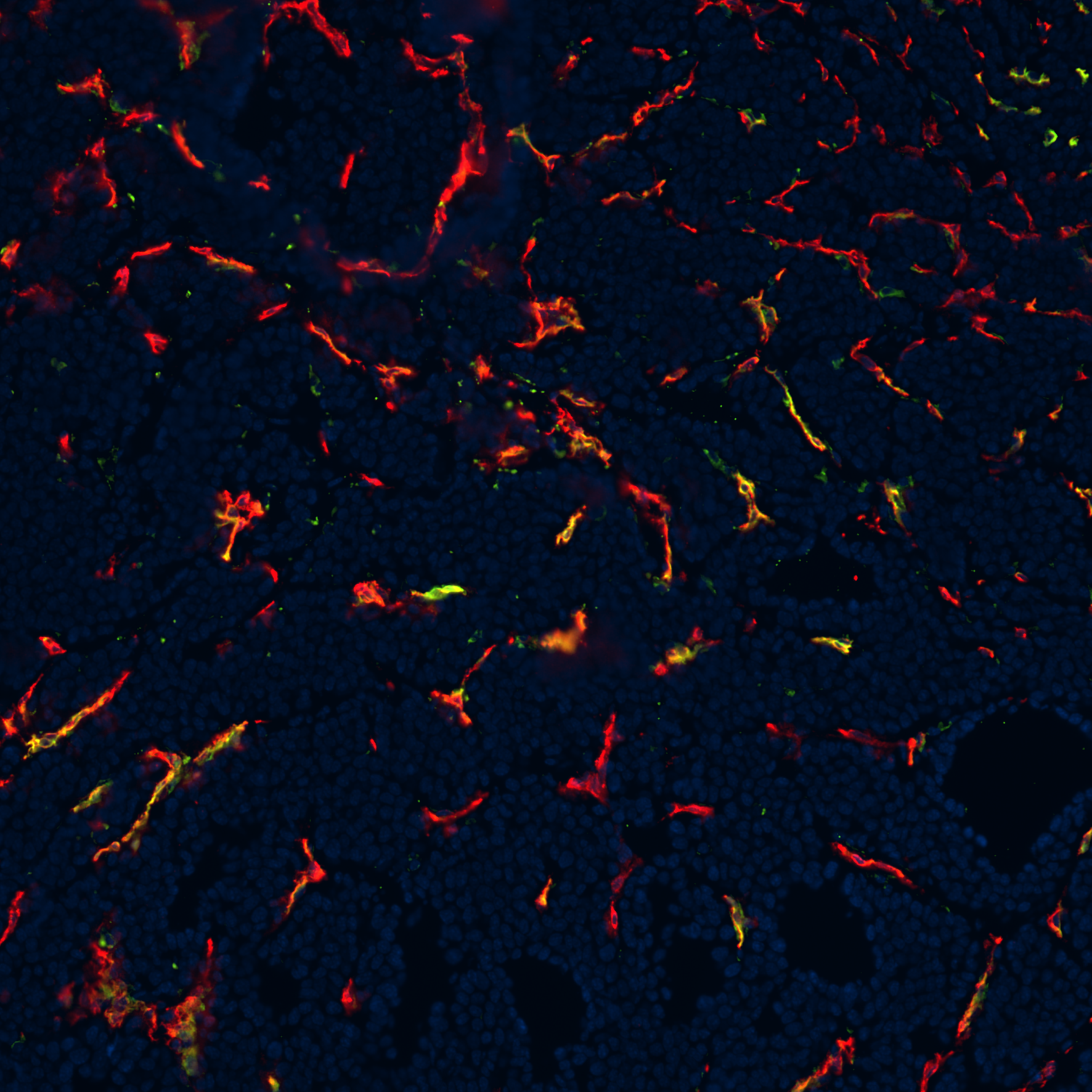

Supplement: Supplementary file 4 — Source data Fig. 2 [file 44321_2025_222_MOESM4_ESM.zip › For EMM submission/Figures 2I/RT5 eribulin + fasudil.tif]

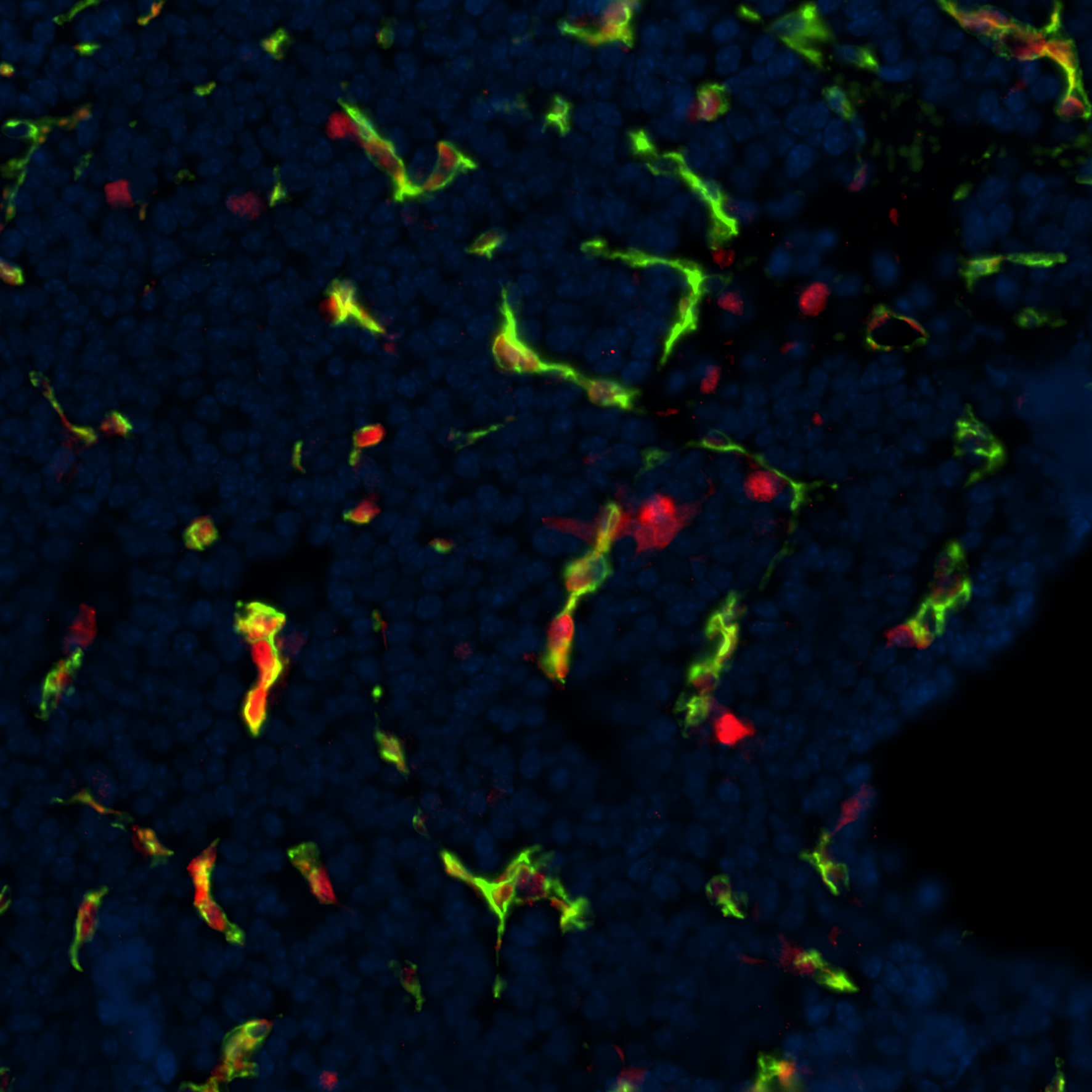

Supplement: Supplementary file 5 — Source data Fig. 3 [file 44321_2025_222_MOESM5_ESM.zip › For EMM submission/Figure 3A/RT5 CA4 - CD31.tif]

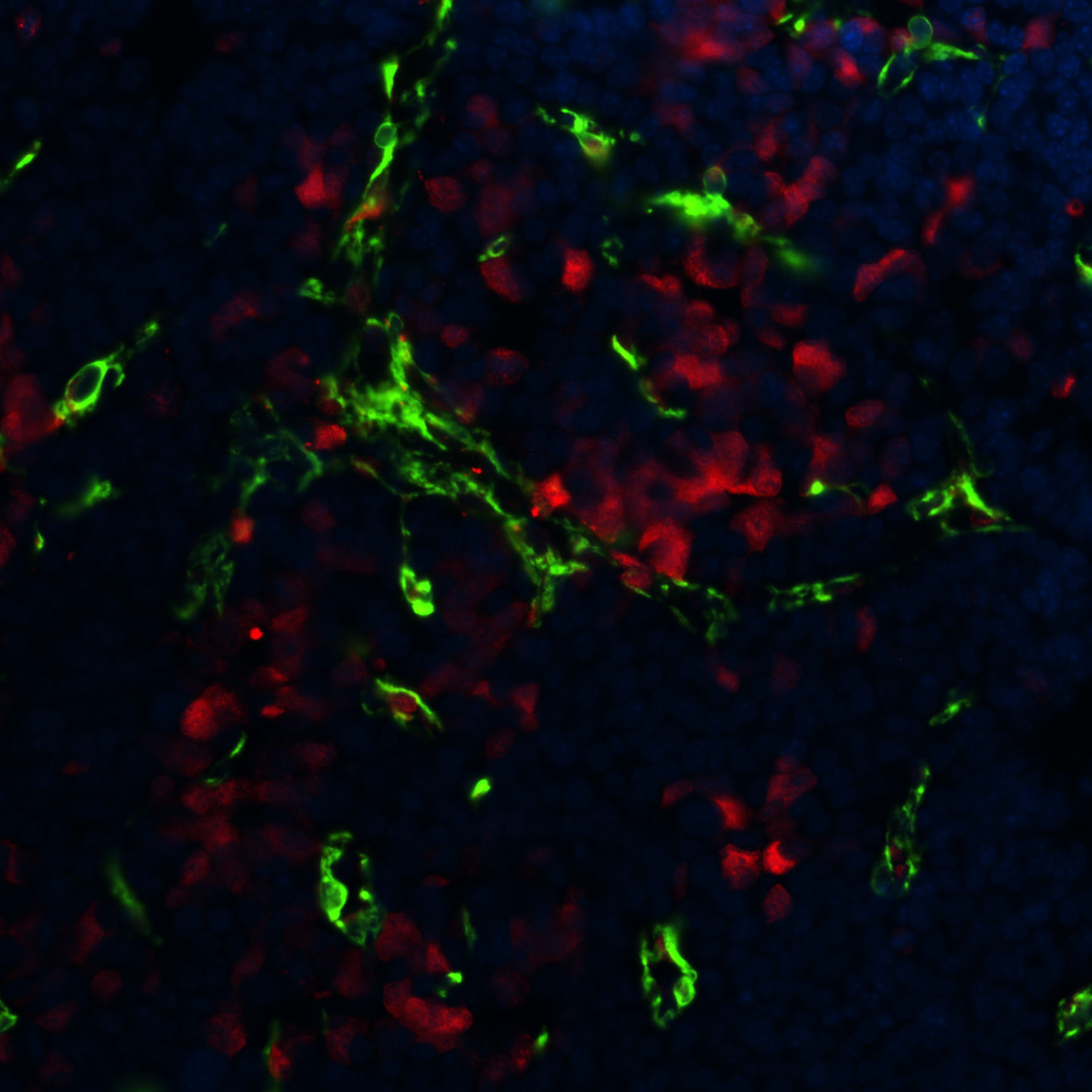

Supplement: Supplementary file 5 — Source data Fig. 3 [file 44321_2025_222_MOESM5_ESM.zip › For EMM submission/Figure 3A/RT5 CA4 - NG2.tif]

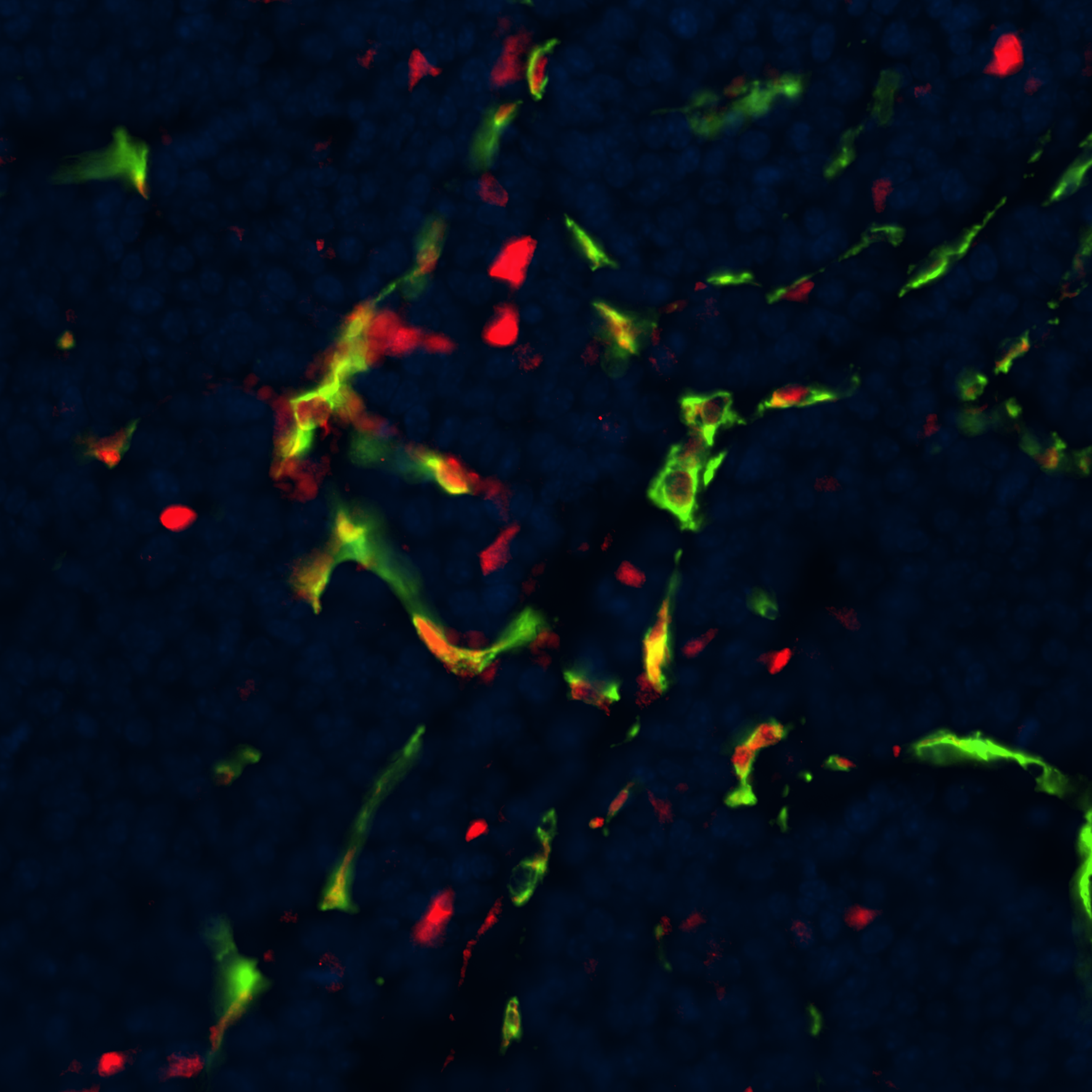

Supplement: Supplementary file 5 — Source data Fig. 3 [file 44321_2025_222_MOESM5_ESM.zip › For EMM submission/Figure 3A/RT5 Eribulin - CD31.tif]

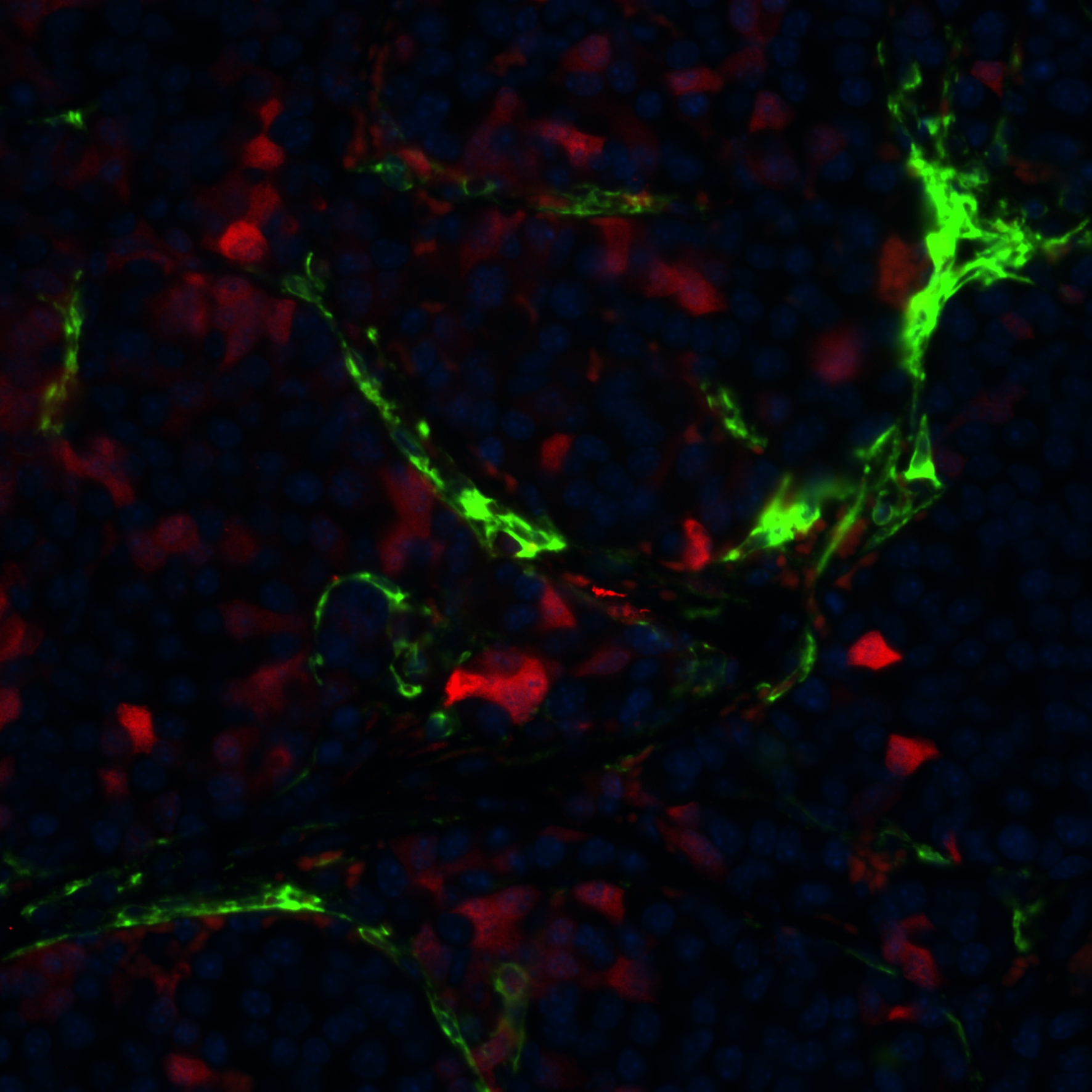

Supplement: Supplementary file 5 — Source data Fig. 3 [file 44321_2025_222_MOESM5_ESM.zip › For EMM submission/Figure 3A/RT5 Eribulin - NG2.tif]

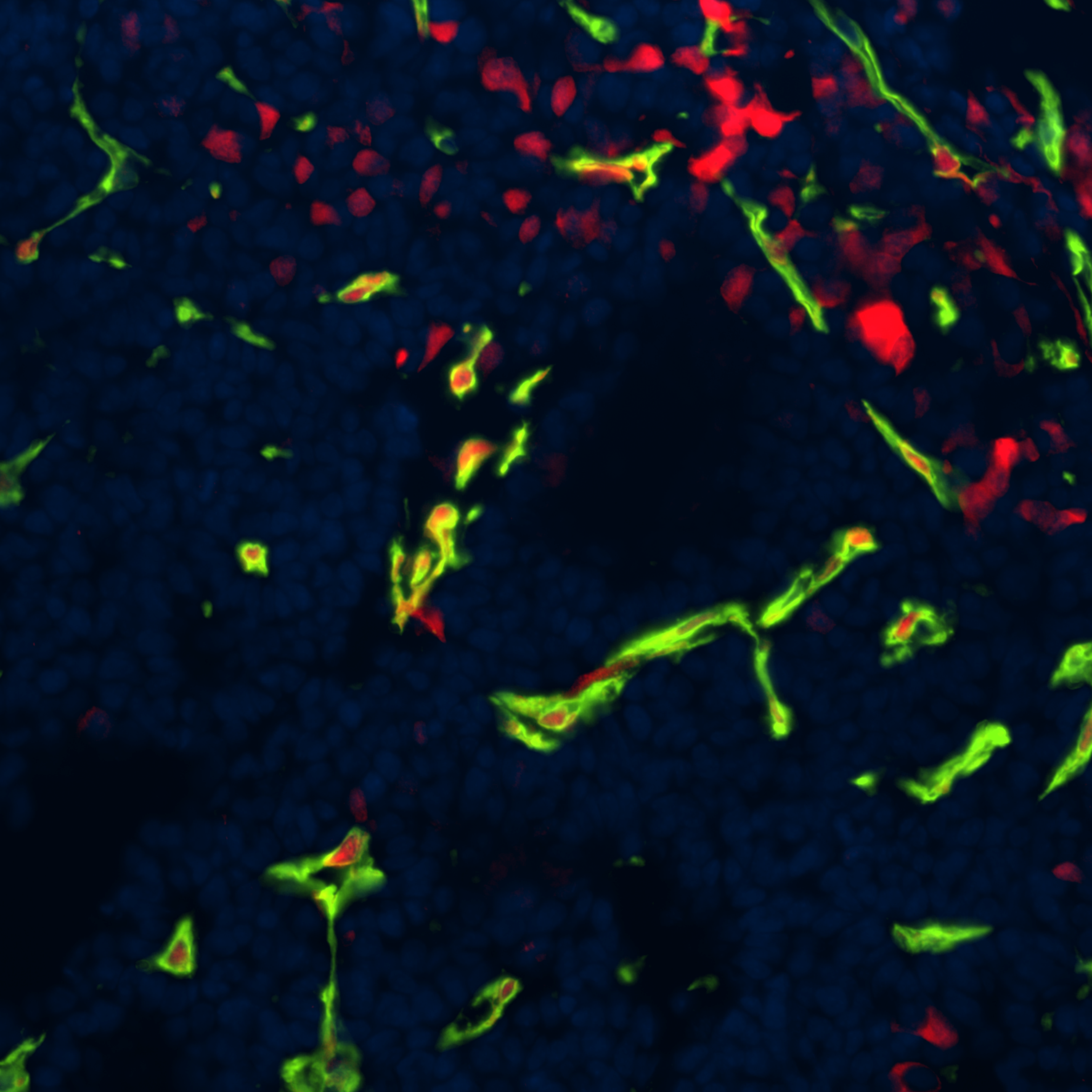

Supplement: Supplementary file 5 — Source data Fig. 3 [file 44321_2025_222_MOESM5_ESM.zip › For EMM submission/Figure 3A/RT5 untreated - CD31.tif]

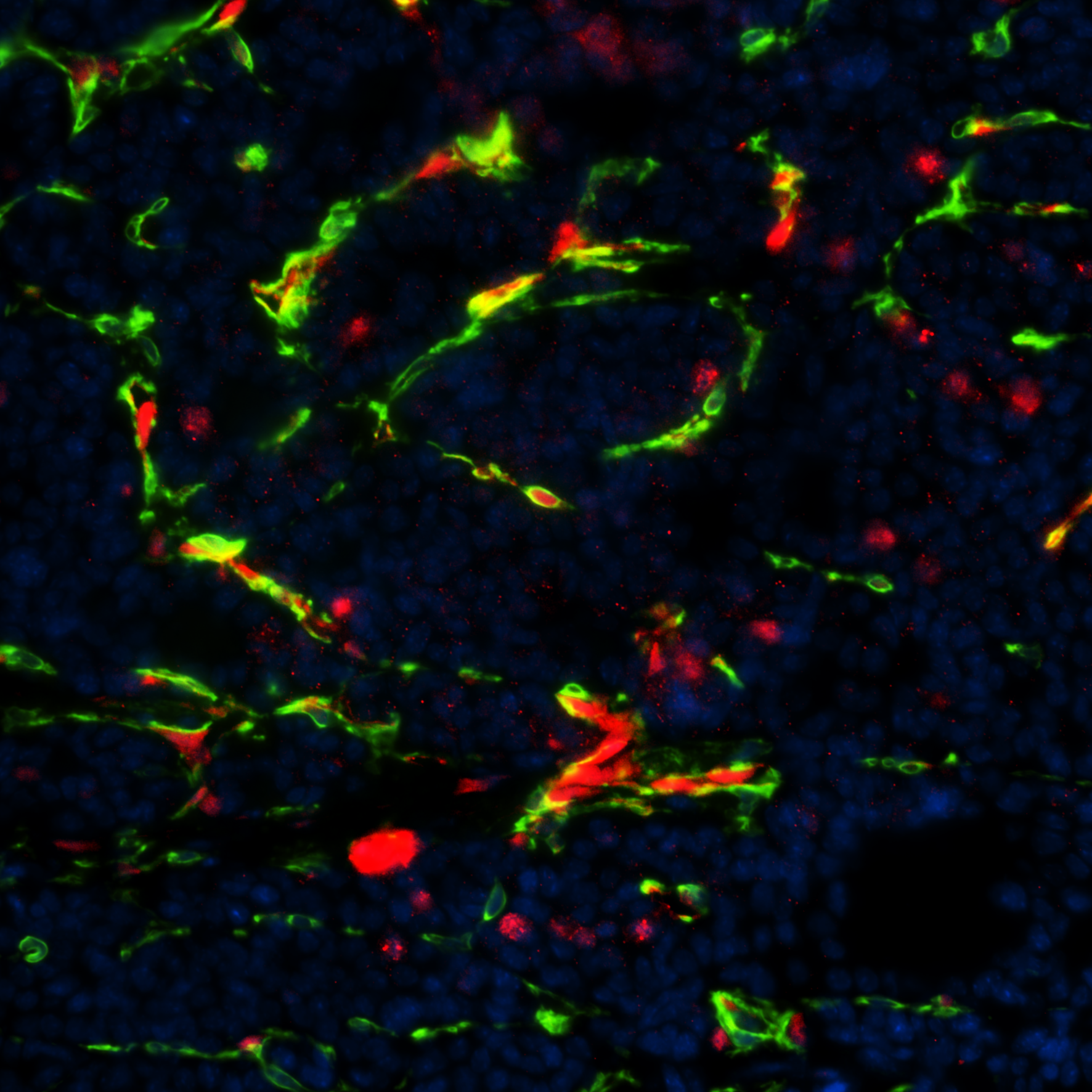

Supplement: Supplementary file 5 — Source data Fig. 3 [file 44321_2025_222_MOESM5_ESM.zip › For EMM submission/Figure 3A/RT5 untreated - NG2.tif]

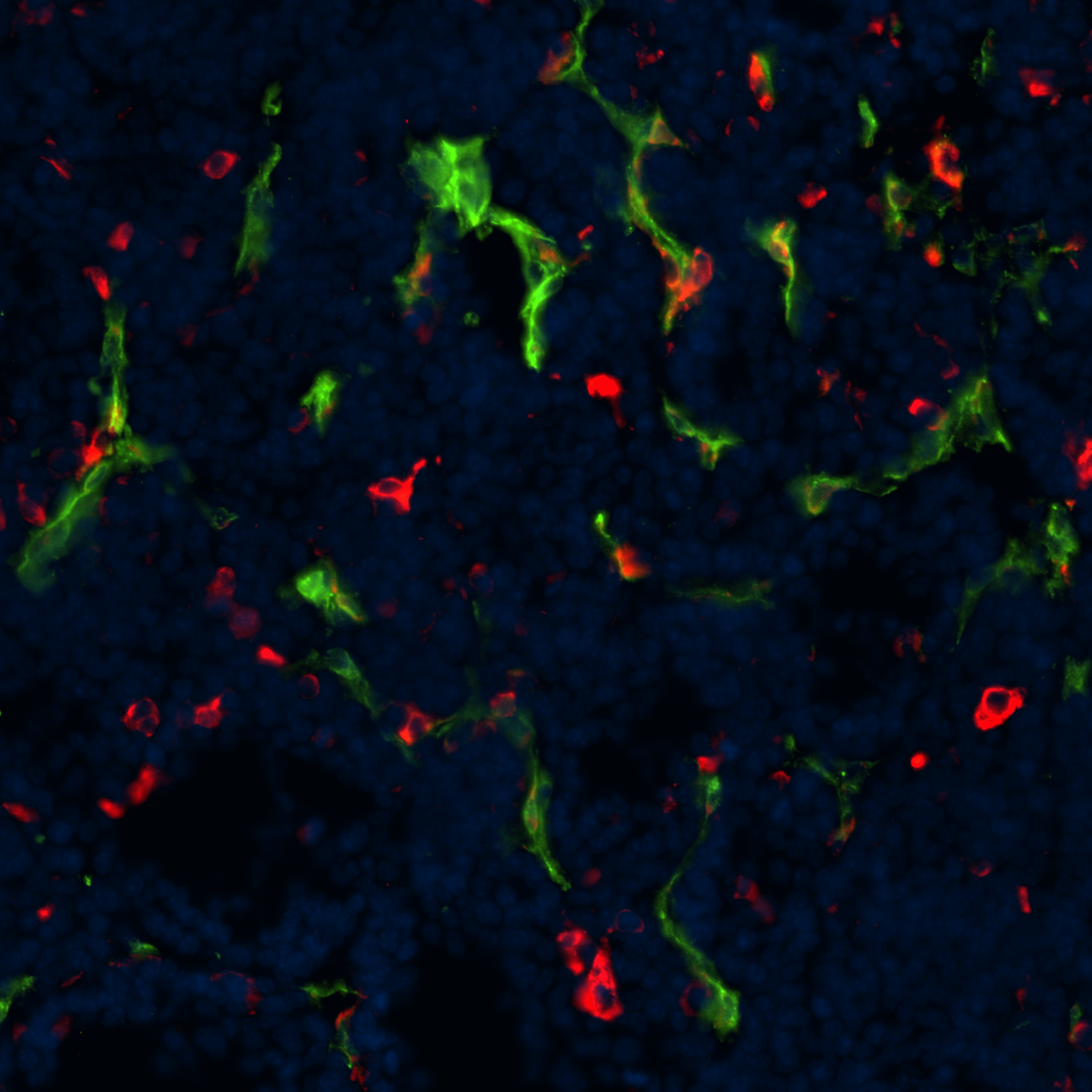

Supplement: Supplementary file 5 — Source data Fig. 3 [file 44321_2025_222_MOESM5_ESM.zip › For EMM submission/Figure 3B/RT5 CD31 CA4.tif]

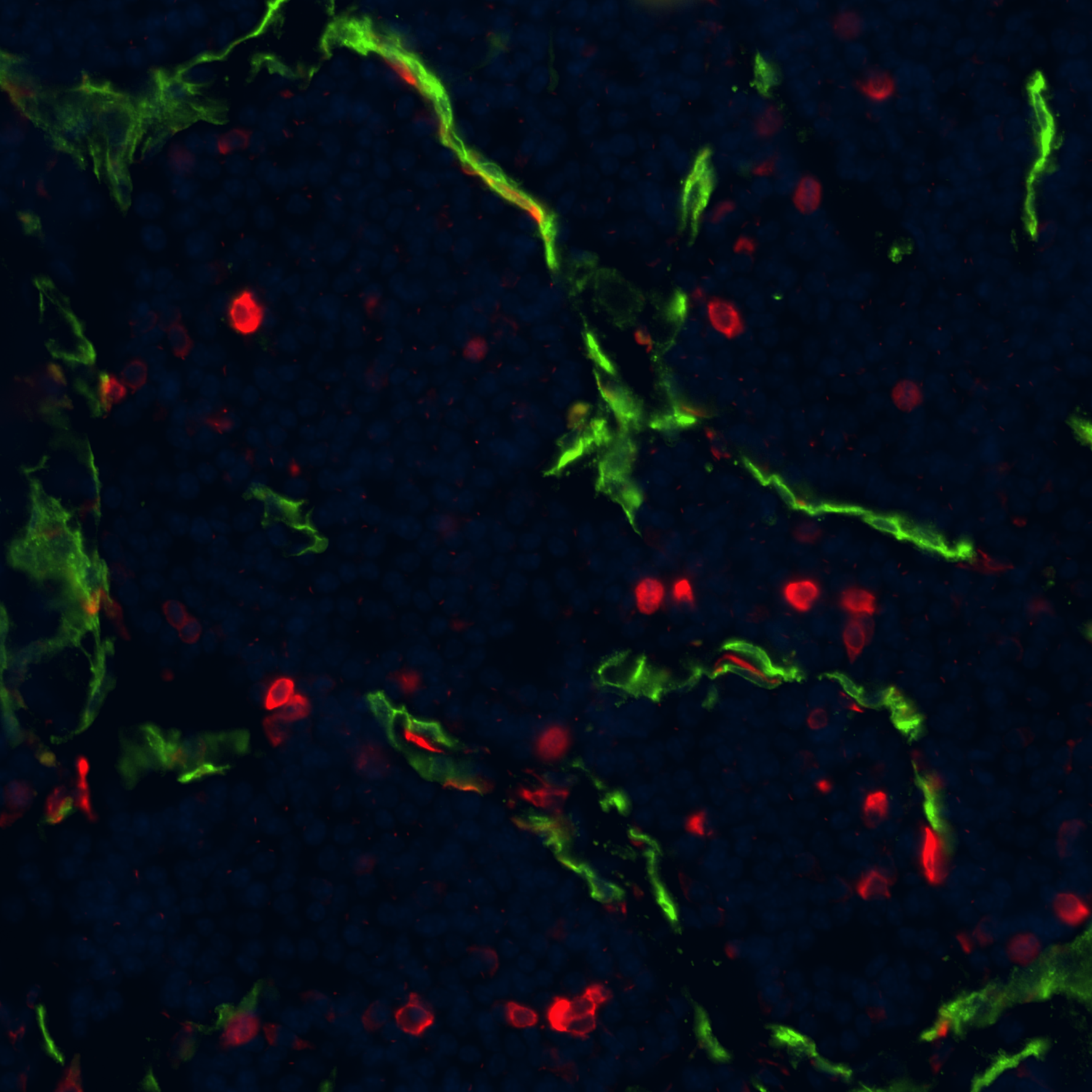

Supplement: Supplementary file 5 — Source data Fig. 3 [file 44321_2025_222_MOESM5_ESM.zip › For EMM submission/Figure 3B/RT5 CD31 Eribulin.tif]

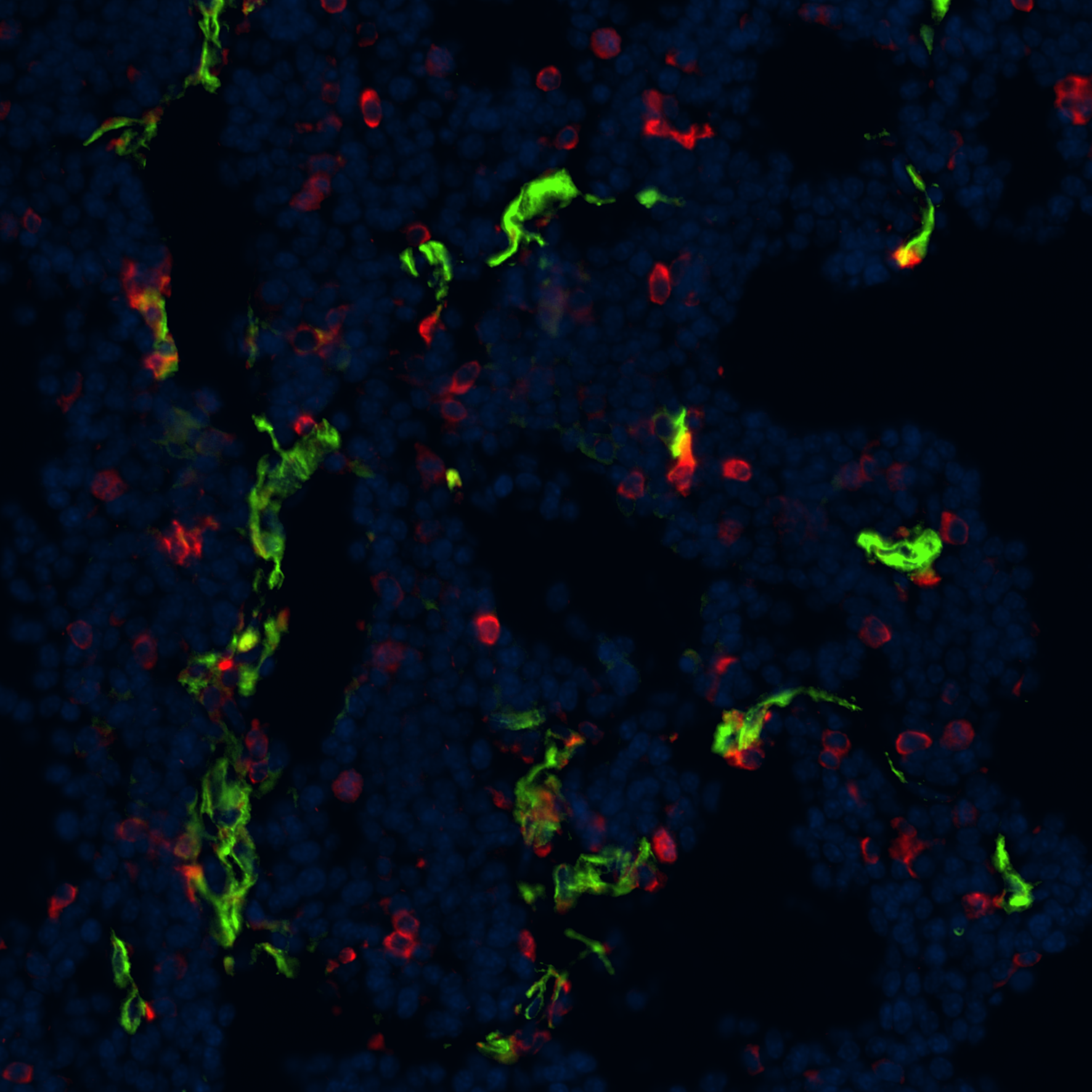

Supplement: Supplementary file 5 — Source data Fig. 3 [file 44321_2025_222_MOESM5_ESM.zip › For EMM submission/Figure 3B/RT5 CD31 untreated.tif]

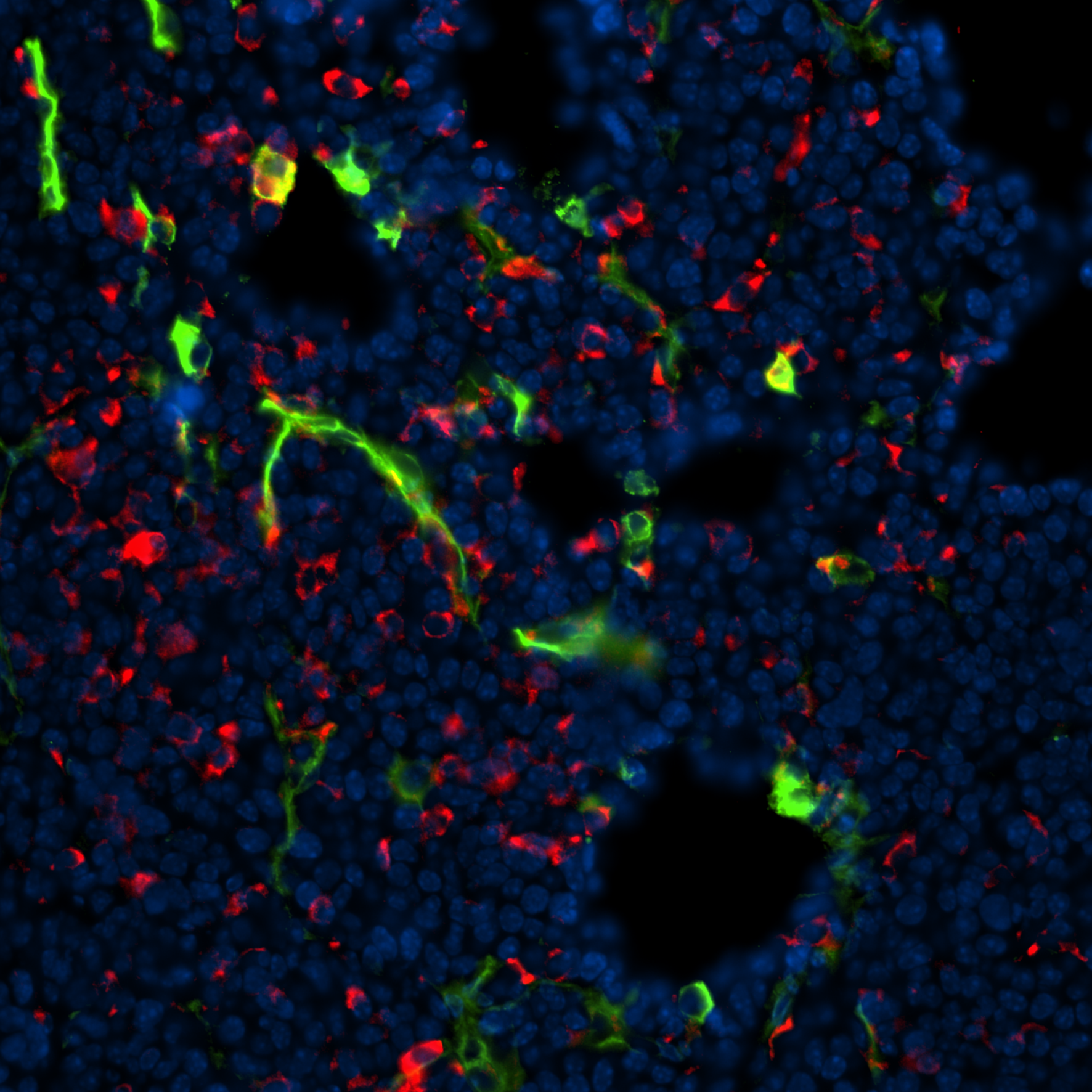

Supplement: Supplementary file 5 — Source data Fig. 3 [file 44321_2025_222_MOESM5_ESM.zip › For EMM submission/Figure 3B/RT5 NG2 CA4.tif]

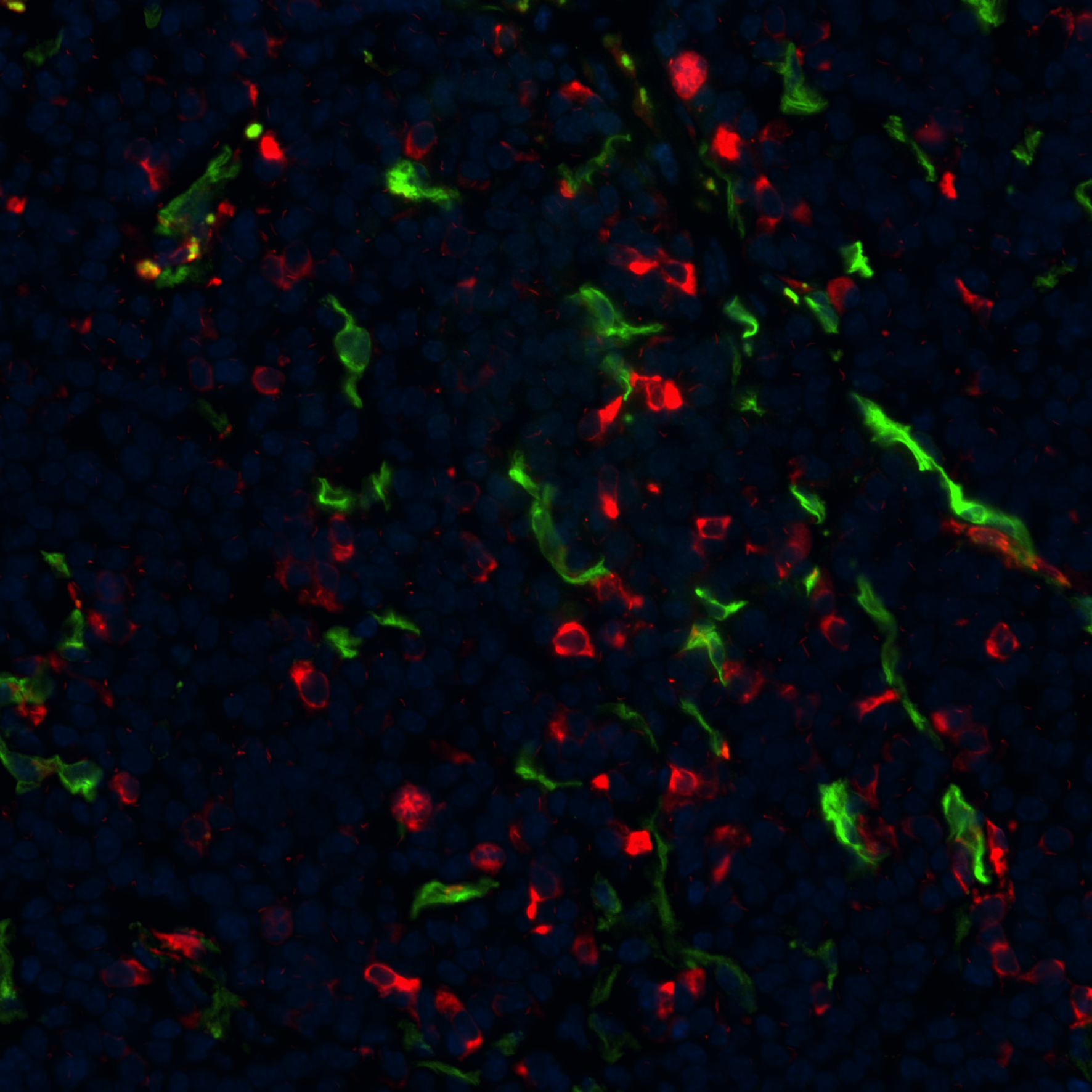

Supplement: Supplementary file 5 — Source data Fig. 3 [file 44321_2025_222_MOESM5_ESM.zip › For EMM submission/Figure 3B/RT5 NG2 Eribulin.tif]

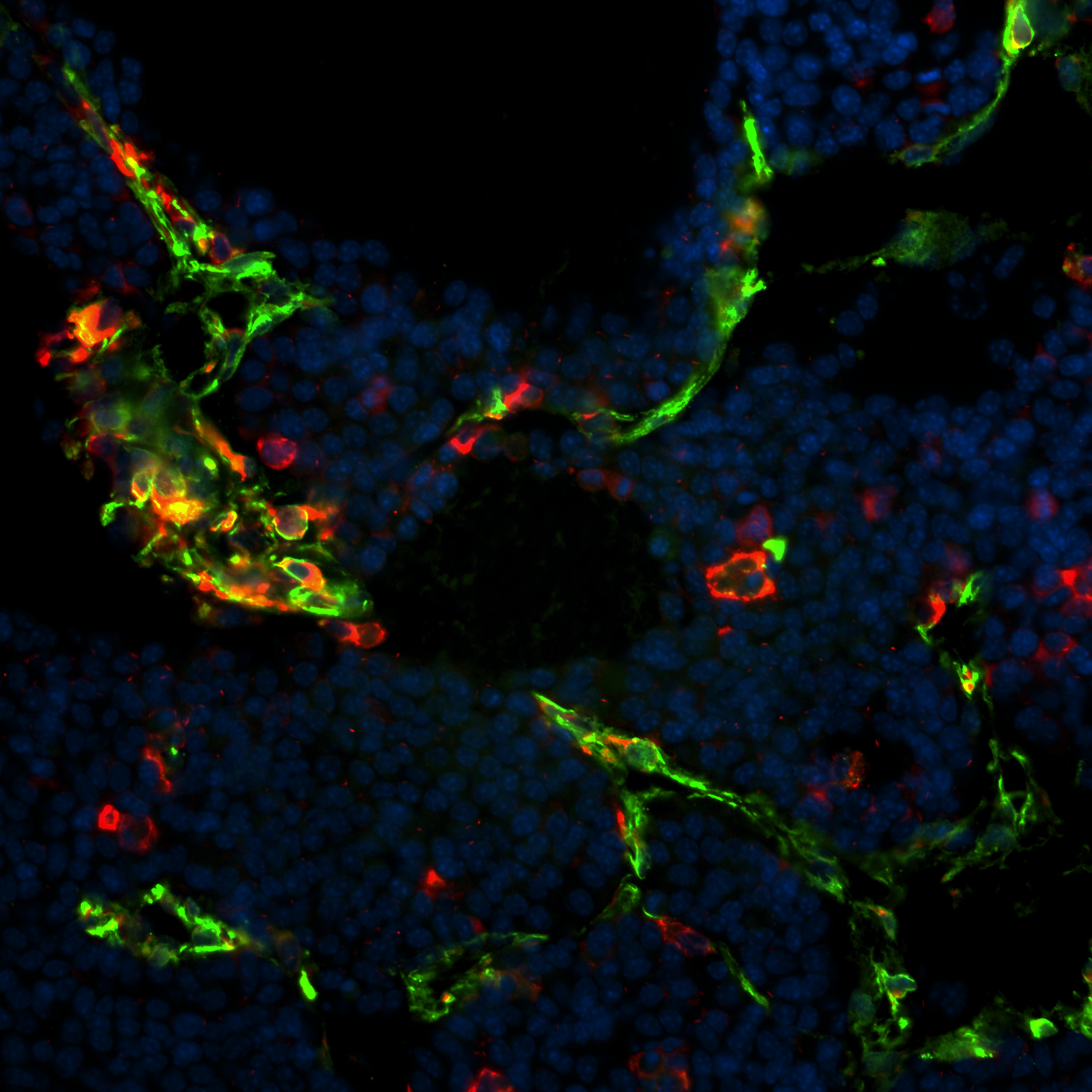

Supplement: Supplementary file 5 — Source data Fig. 3 [file 44321_2025_222_MOESM5_ESM.zip › For EMM submission/Figure 3B/RT5 NG2 untreated.tif]

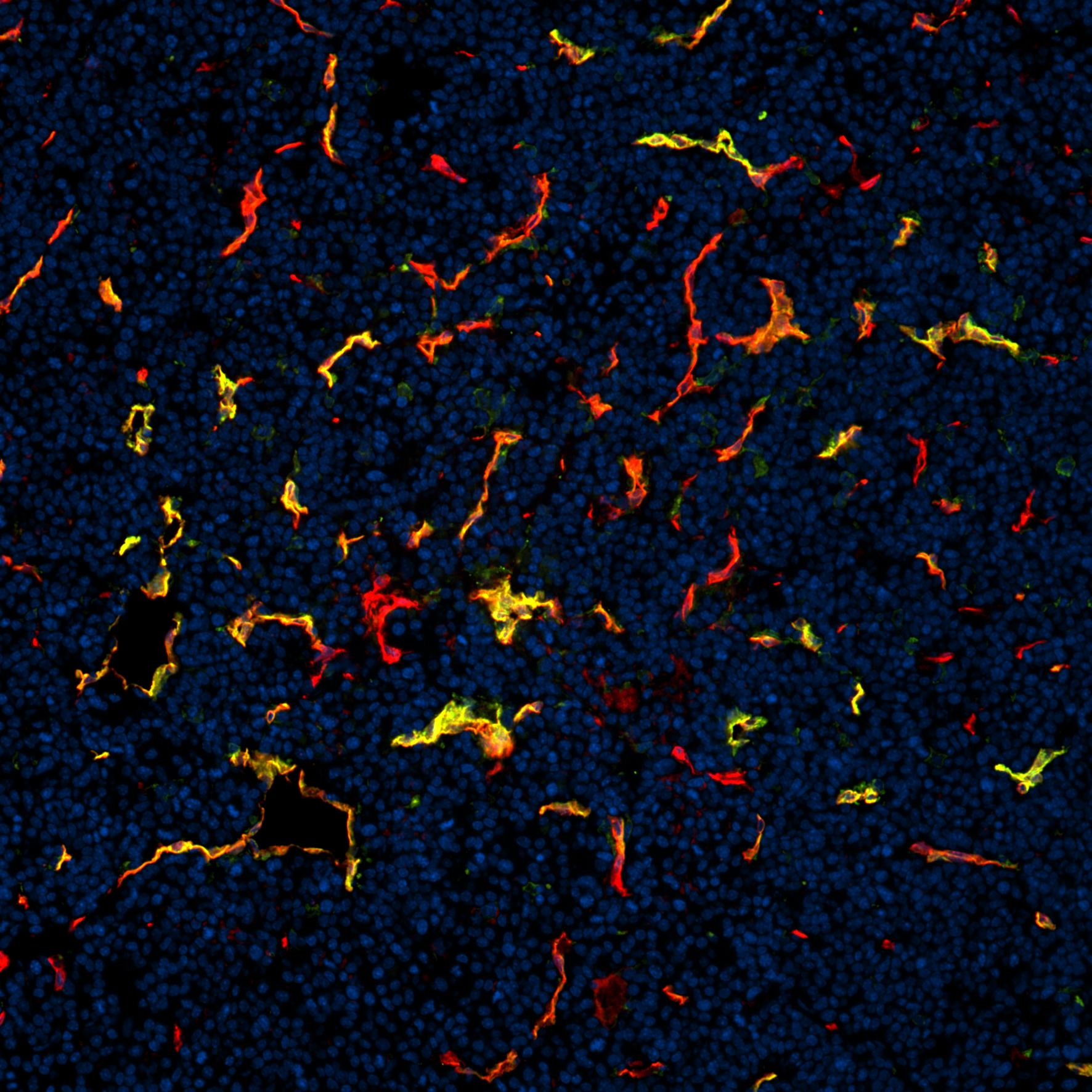

Supplement: Supplementary file 6 — Source data Fig. 4 [file 44321_2025_222_MOESM6_ESM.zip › For EMM submission/Figure 4B/RT5_CA4.tif]

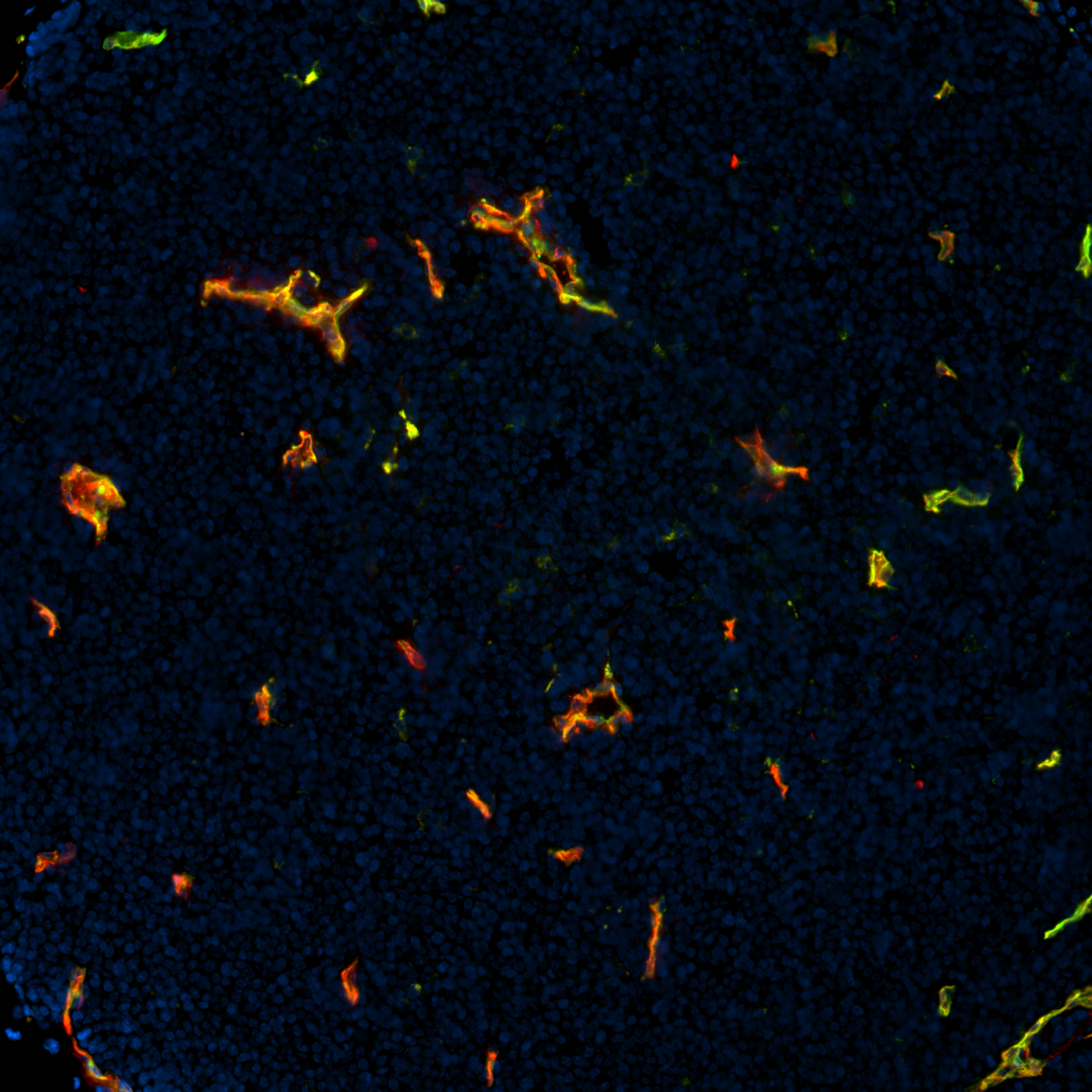

Supplement: Supplementary file 6 — Source data Fig. 4 [file 44321_2025_222_MOESM6_ESM.zip › For EMM submission/Figure 4B/RT5_DC101.tif]

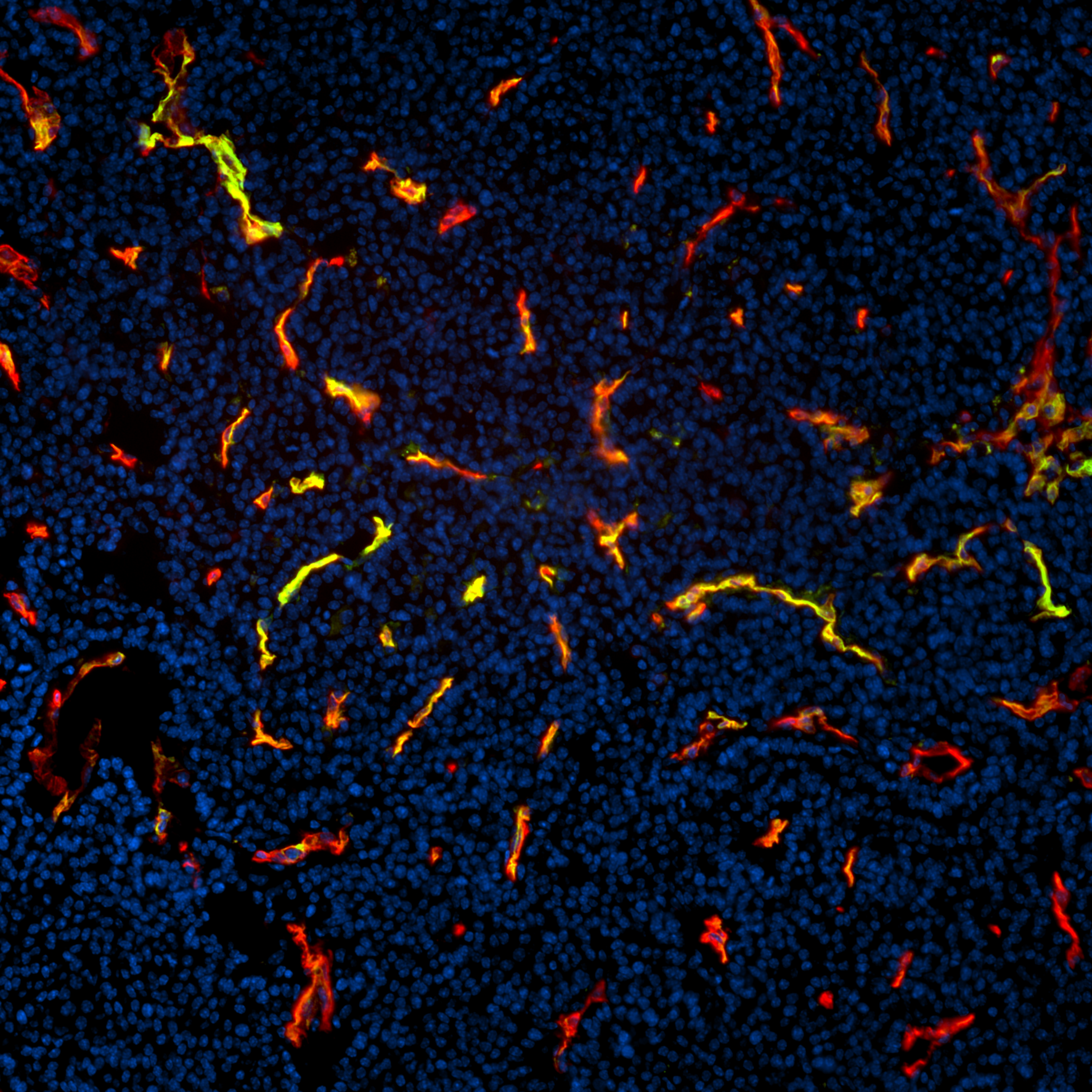

Supplement: Supplementary file 6 — Source data Fig. 4 [file 44321_2025_222_MOESM6_ESM.zip › For EMM submission/Figure 4B/RT5_Eribulin.tif]

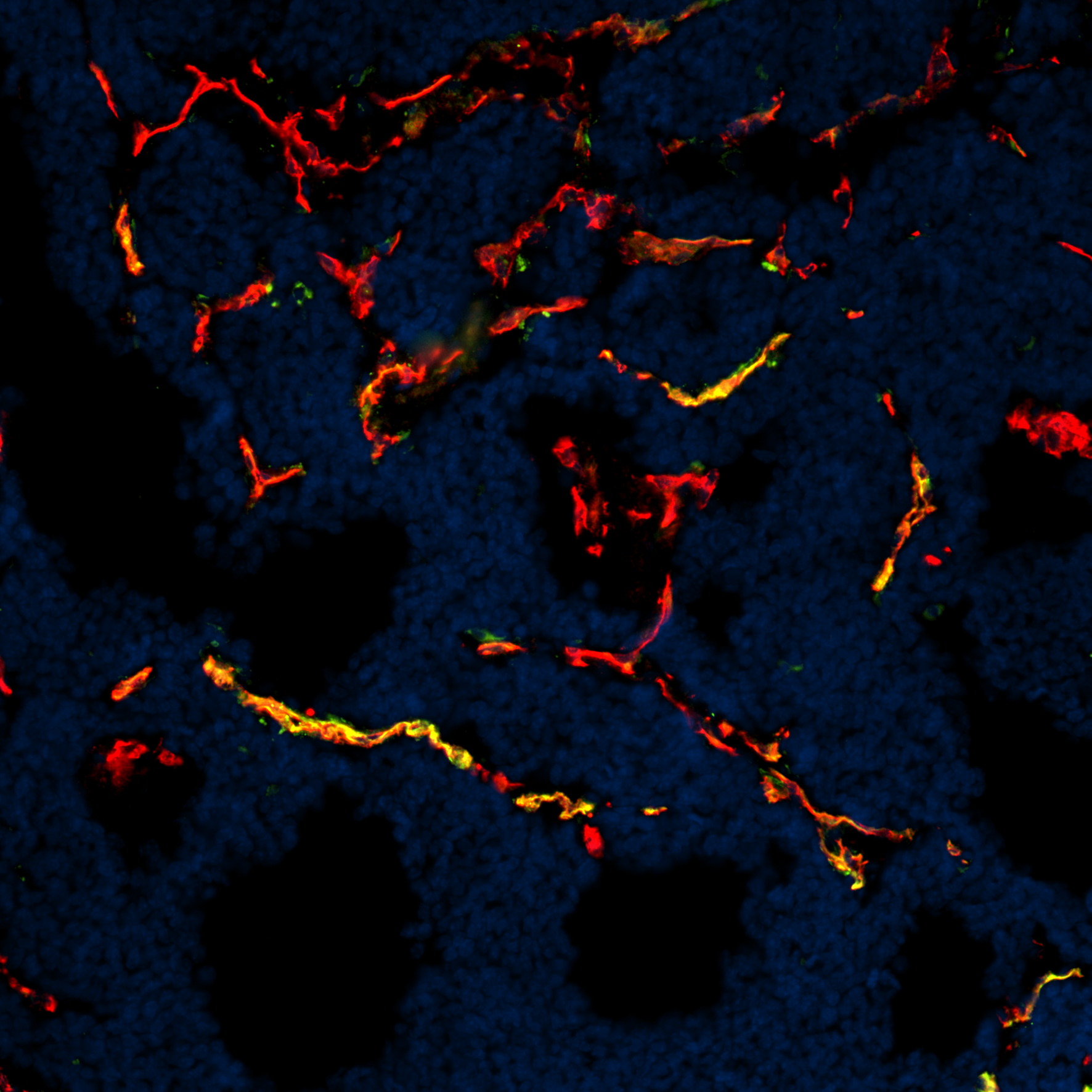

Supplement: Supplementary file 6 — Source data Fig. 4 [file 44321_2025_222_MOESM6_ESM.zip › For EMM submission/Figure 4B/RT5_Untreated.tif]

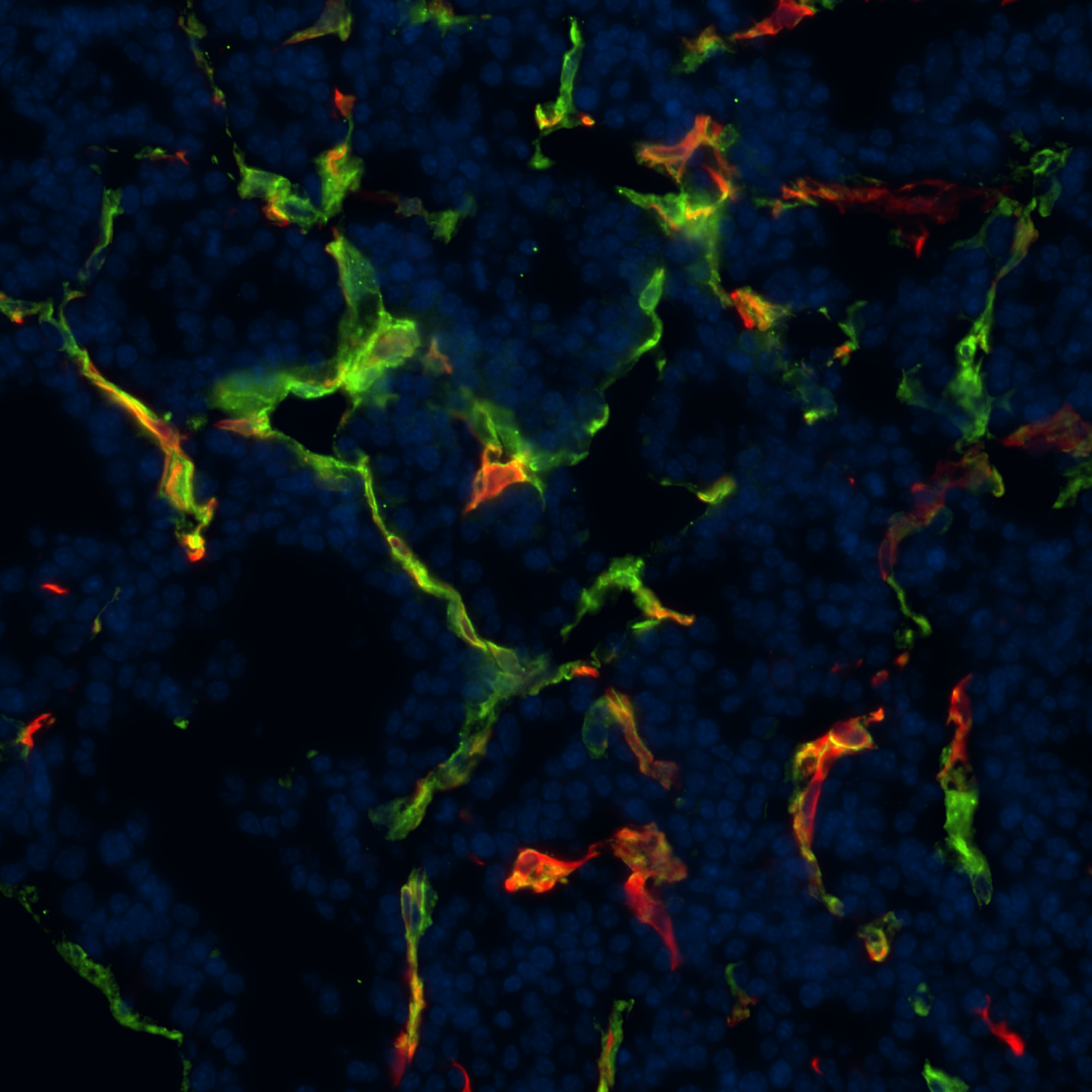

Supplement: Supplementary file 6 — Source data Fig. 4 [file 44321_2025_222_MOESM6_ESM.zip › For EMM submission/Figure 4C/RT5_CA4.tif]

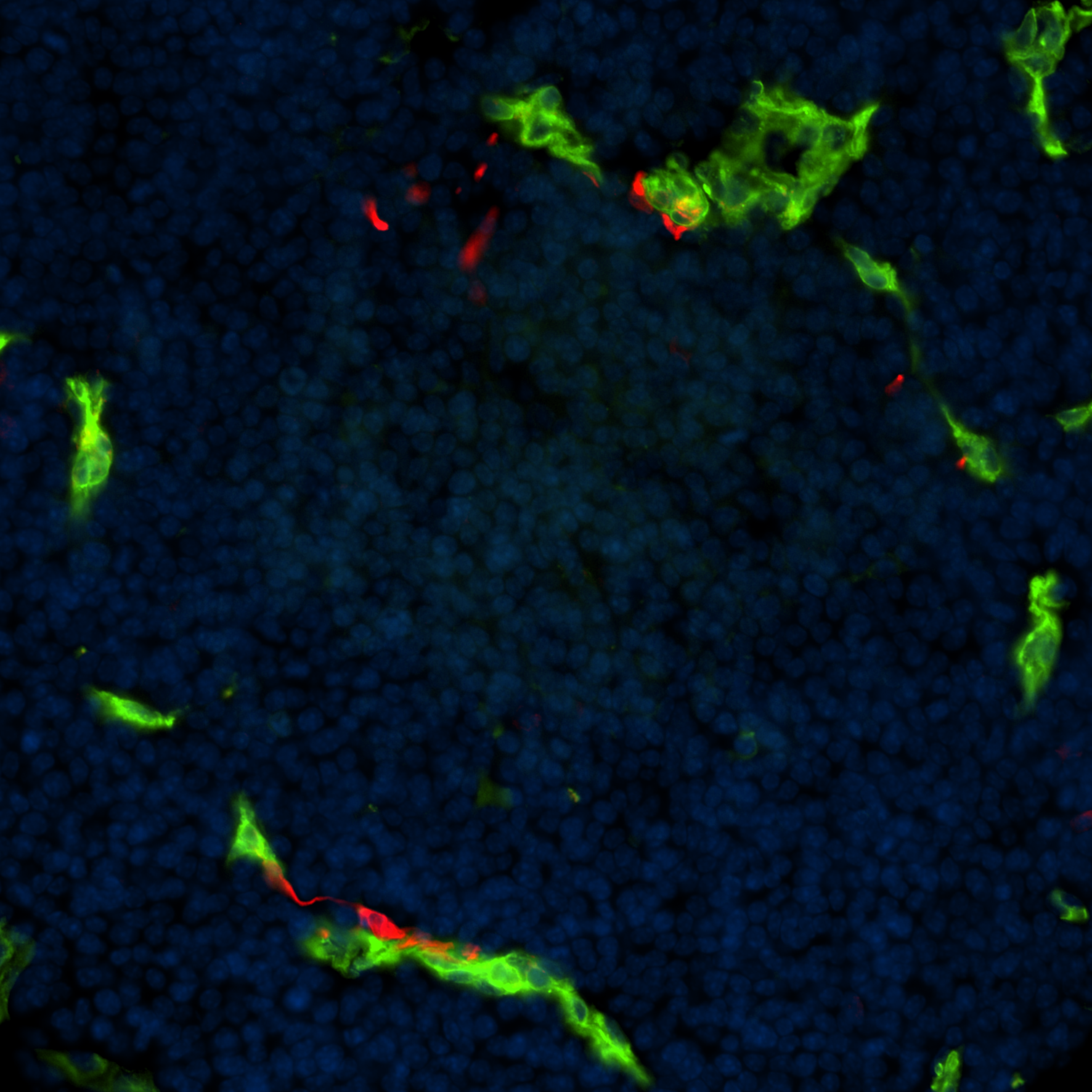

Supplement: Supplementary file 6 — Source data Fig. 4 [file 44321_2025_222_MOESM6_ESM.zip › For EMM submission/Figure 4C/RT5_DC101.tif]

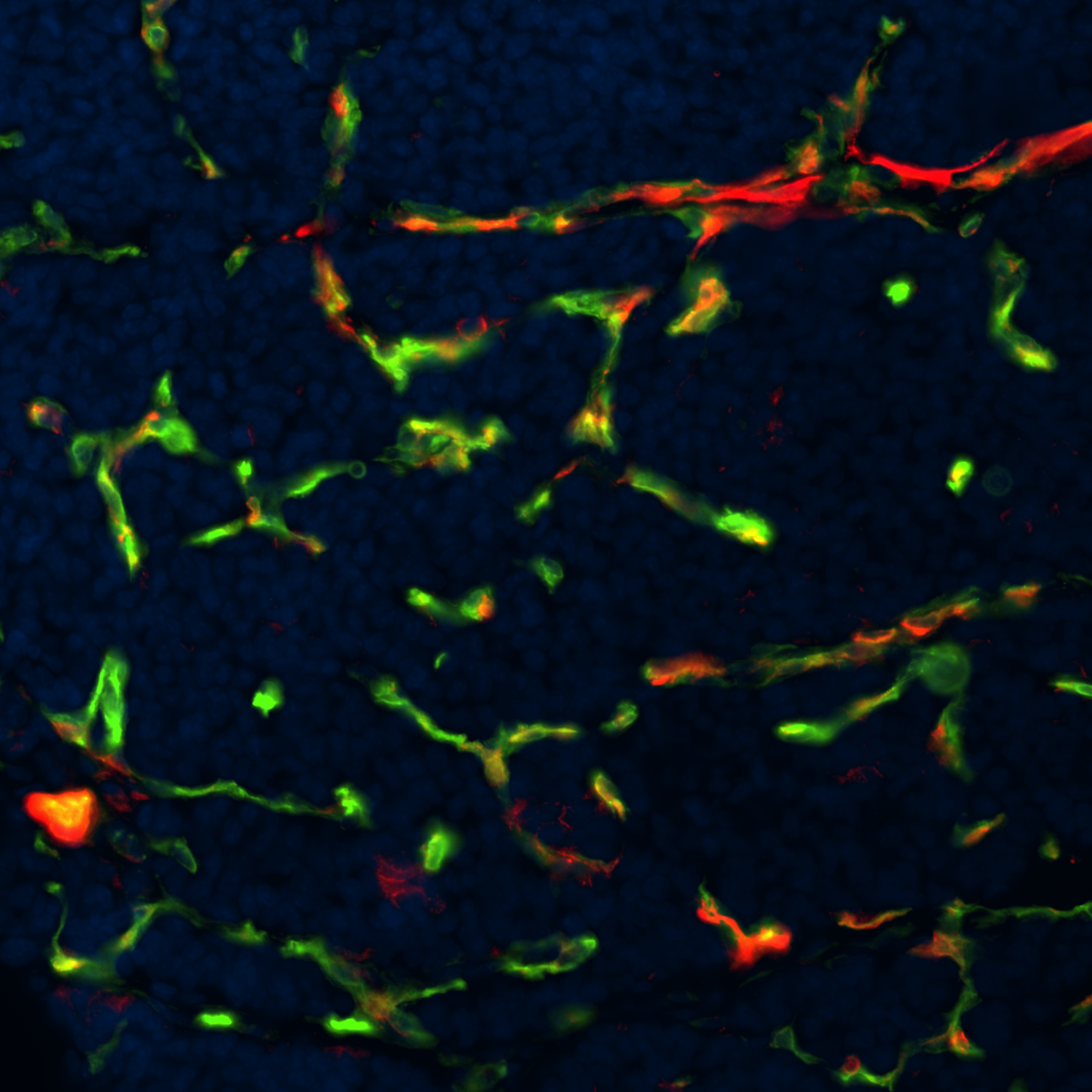

Supplement: Supplementary file 6 — Source data Fig. 4 [file 44321_2025_222_MOESM6_ESM.zip › For EMM submission/Figure 4C/RT5_Eribulin.tif]

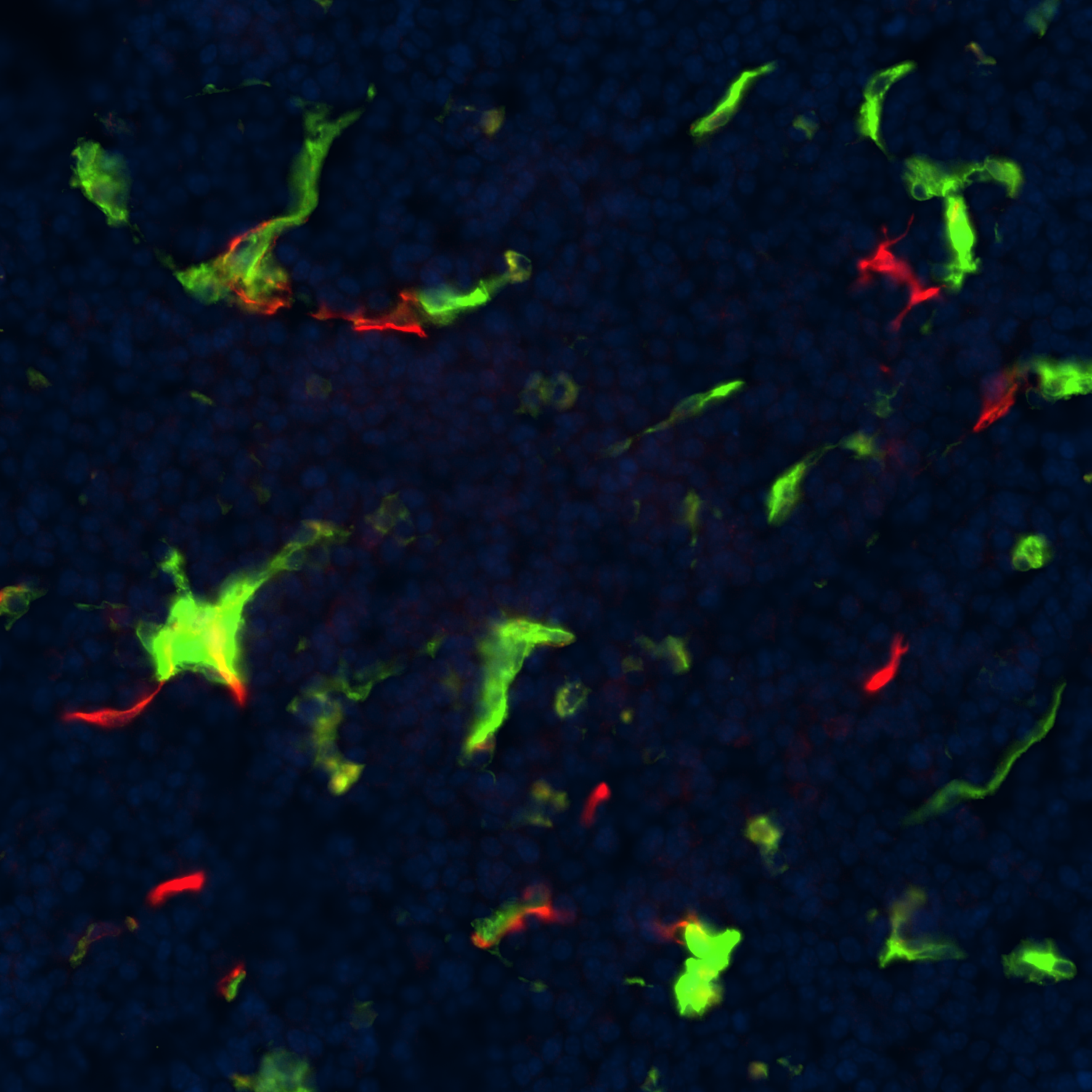

Supplement: Supplementary file 6 — Source data Fig. 4 [file 44321_2025_222_MOESM6_ESM.zip › For EMM submission/Figure 4C/RT5_Untreated.tif]

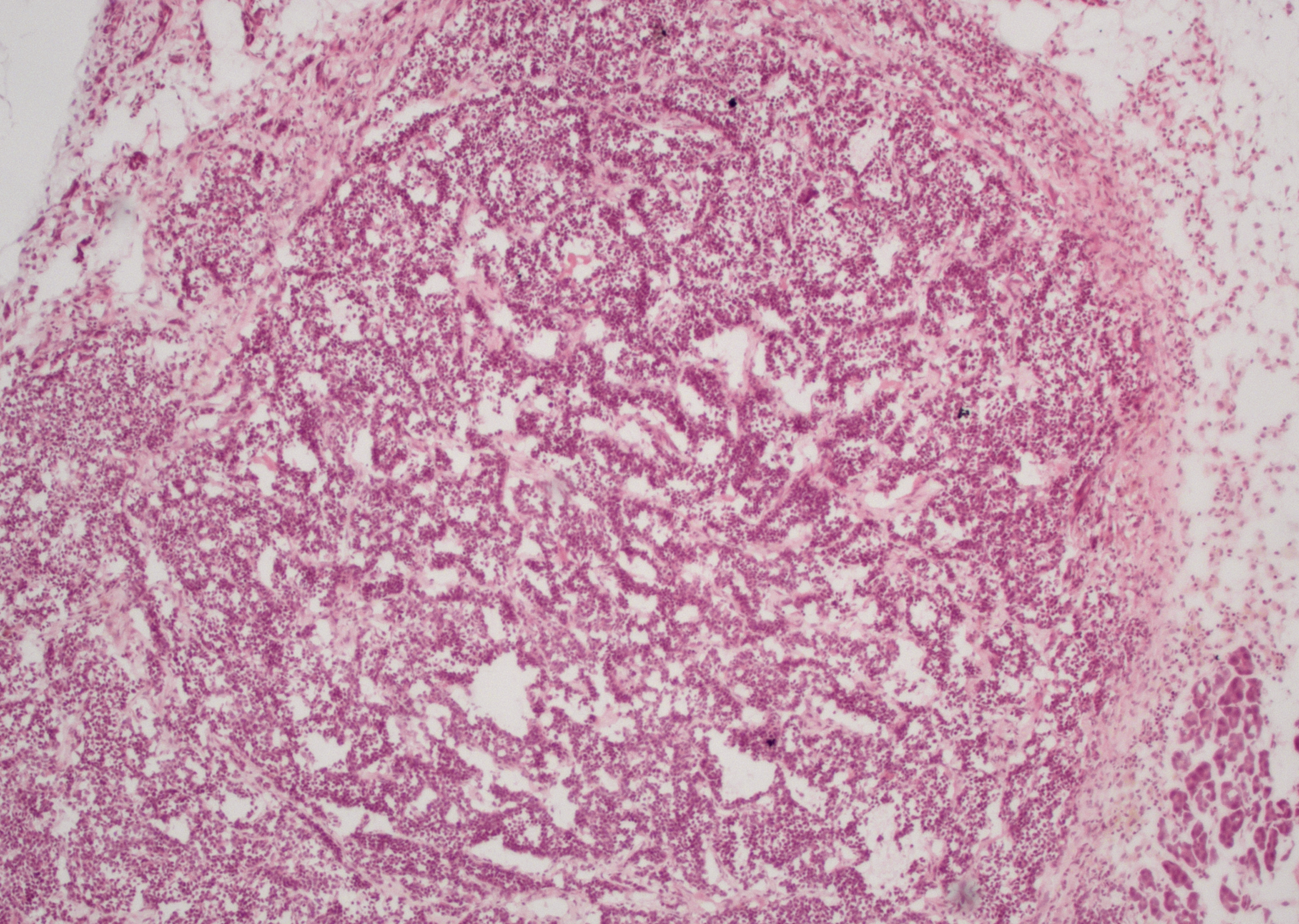

Supplement: Supplementary file 6 — Source data Fig. 4 [file 44321_2025_222_MOESM6_ESM.zip › For EMM submission/Figure 4E/RT5_LT_CA4.tif]

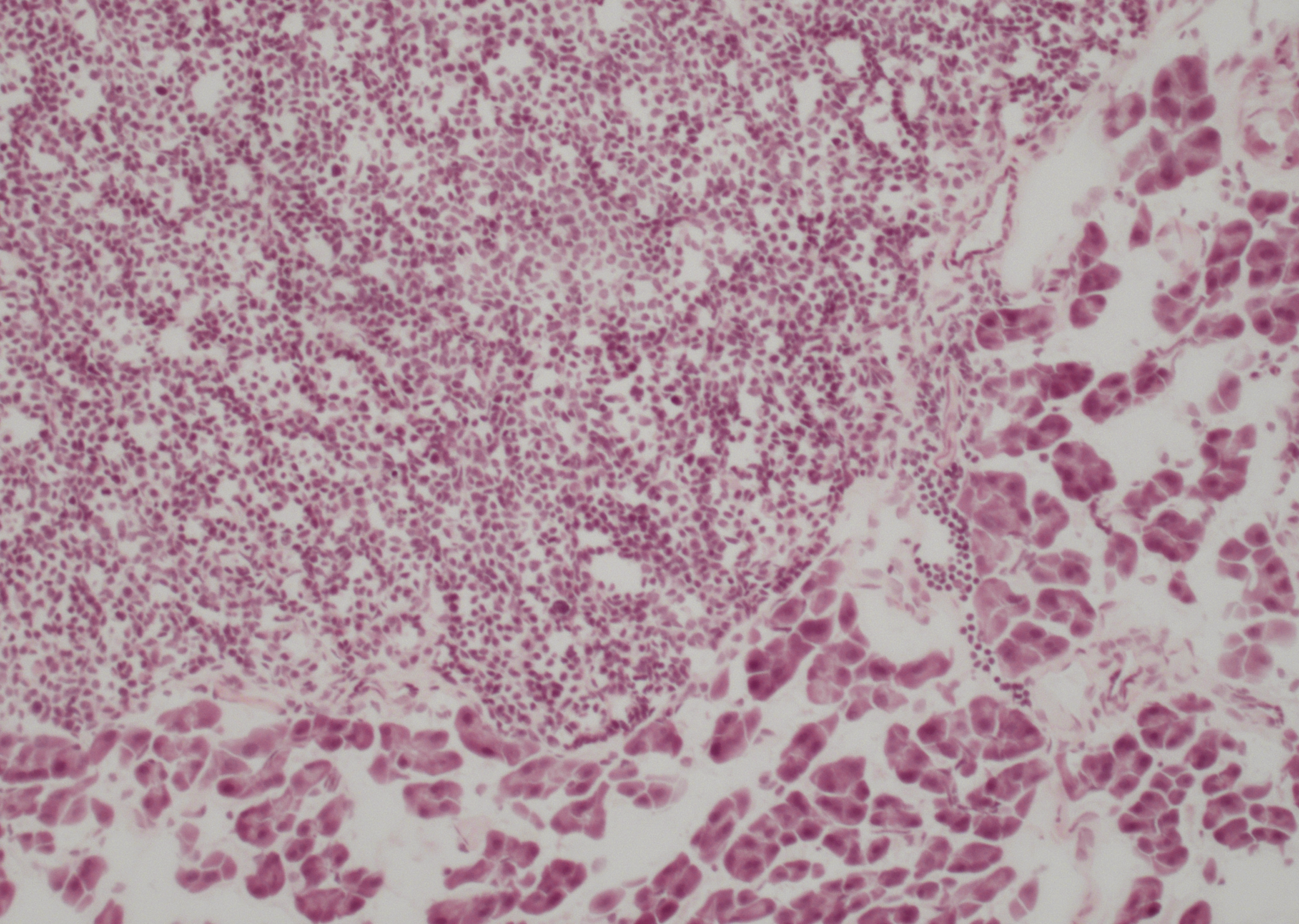

Supplement: Supplementary file 6 — Source data Fig. 4 [file 44321_2025_222_MOESM6_ESM.zip › For EMM submission/Figure 4E/RT5_LT_DC101-10 x.tif]

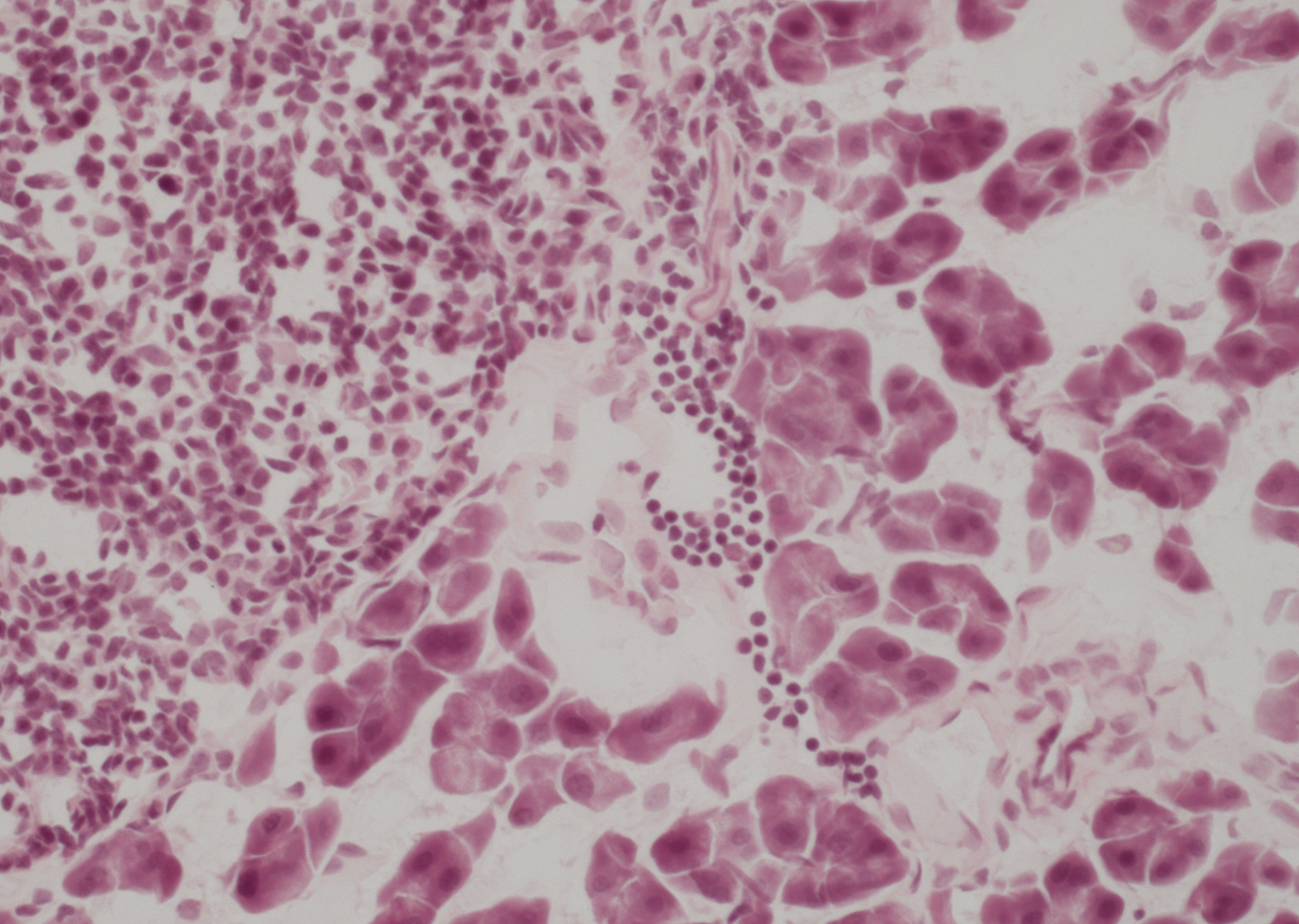

Supplement: Supplementary file 6 — Source data Fig. 4 [file 44321_2025_222_MOESM6_ESM.zip › For EMM submission/Figure 4E/RT5_LT_DC101-20 x.tif]

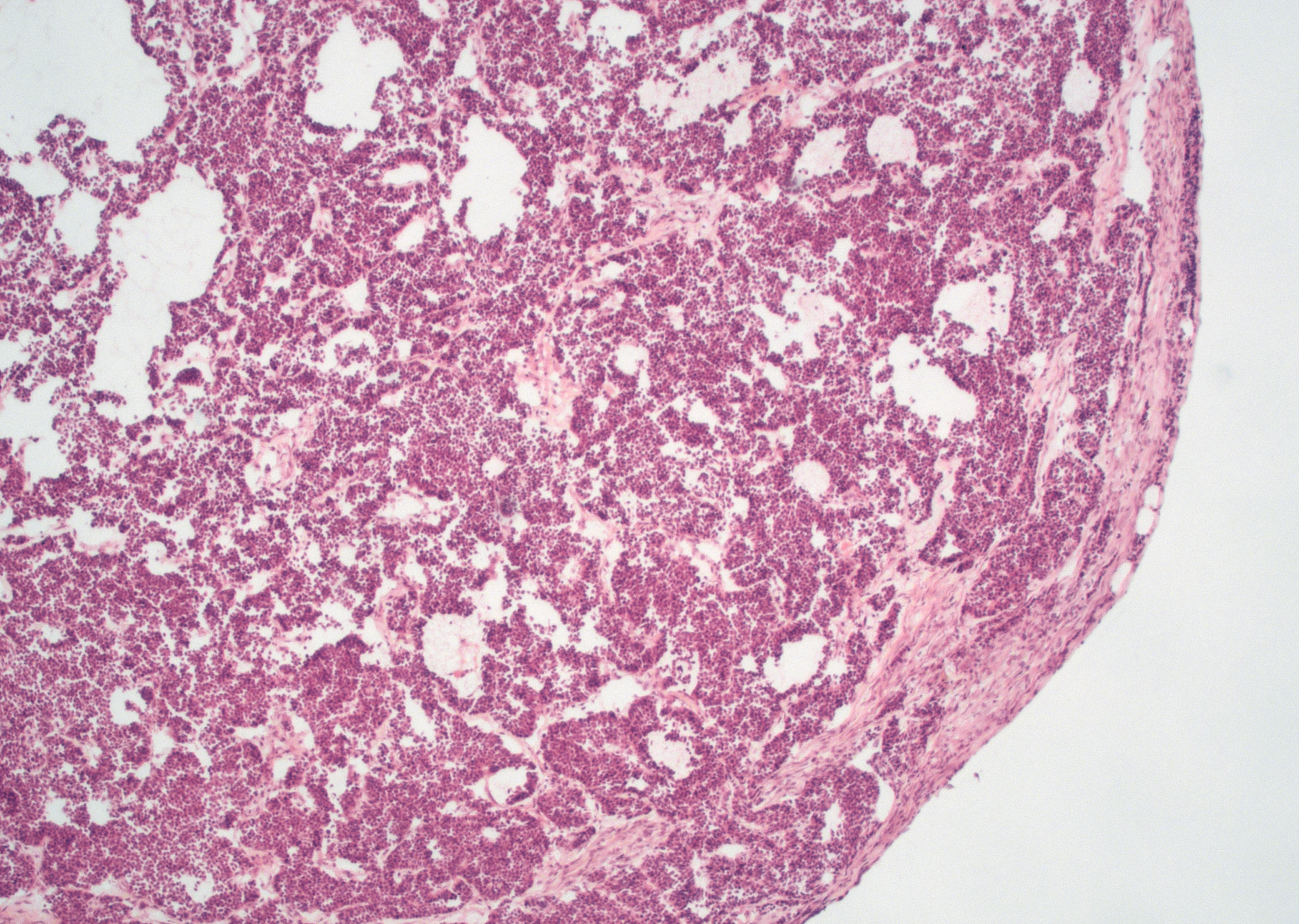

Supplement: Supplementary file 6 — Source data Fig. 4 [file 44321_2025_222_MOESM6_ESM.zip › For EMM submission/Figure 4E/RT5_LT_Erib.tif]

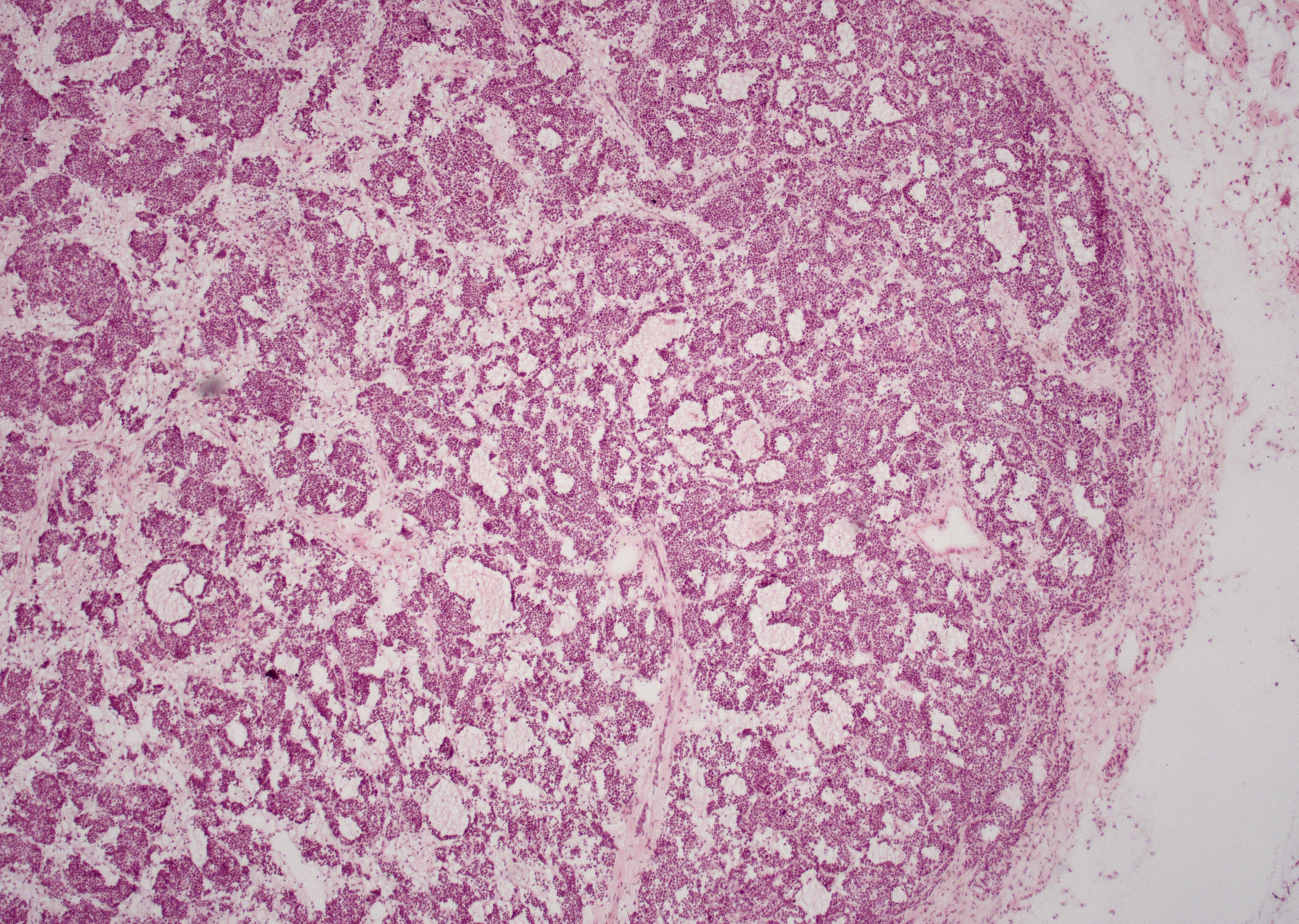

Supplement: Supplementary file 6 — Source data Fig. 4 [file 44321_2025_222_MOESM6_ESM.zip › For EMM submission/Figure 4E/RT5_LT_Untr.tif]

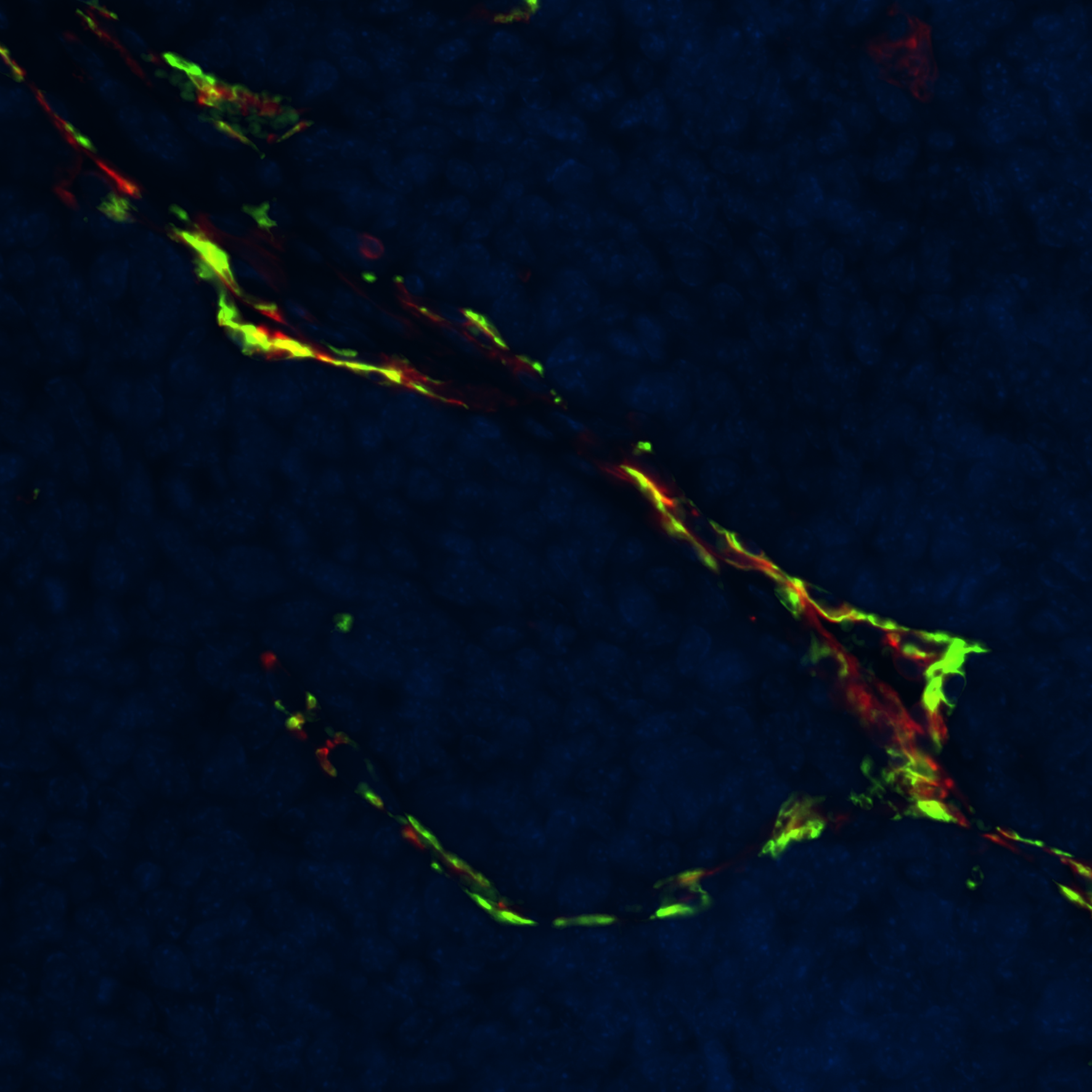

Supplement: Supplementary file 7 — Source data Fig. 5 [file 44321_2025_222_MOESM7_ESM.zip › For EMM submission/Figure 5B/AT3_CA4.tif]

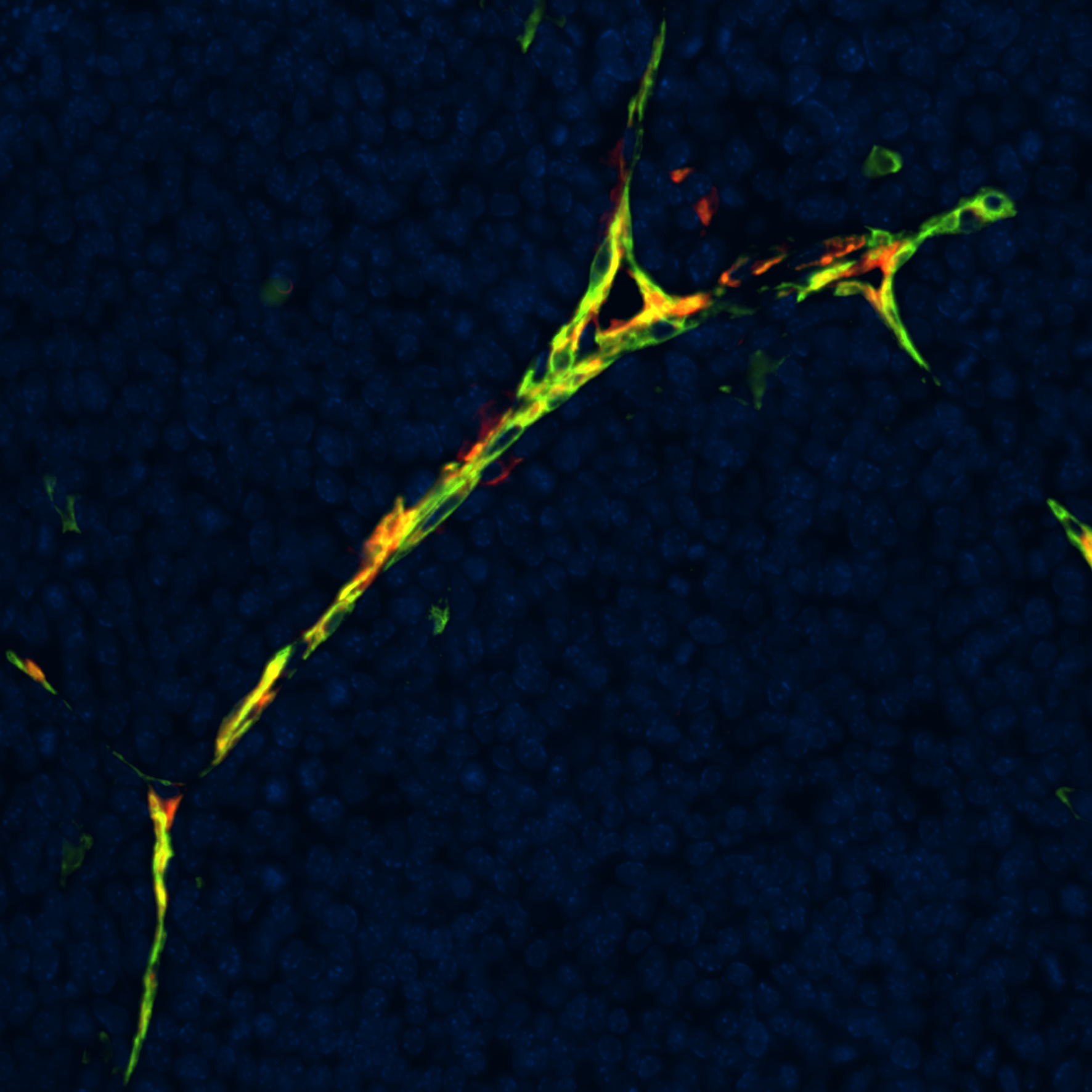

Supplement: Supplementary file 7 — Source data Fig. 5 [file 44321_2025_222_MOESM7_ESM.zip › For EMM submission/Figure 5B/AT3_Eribulin.tif]

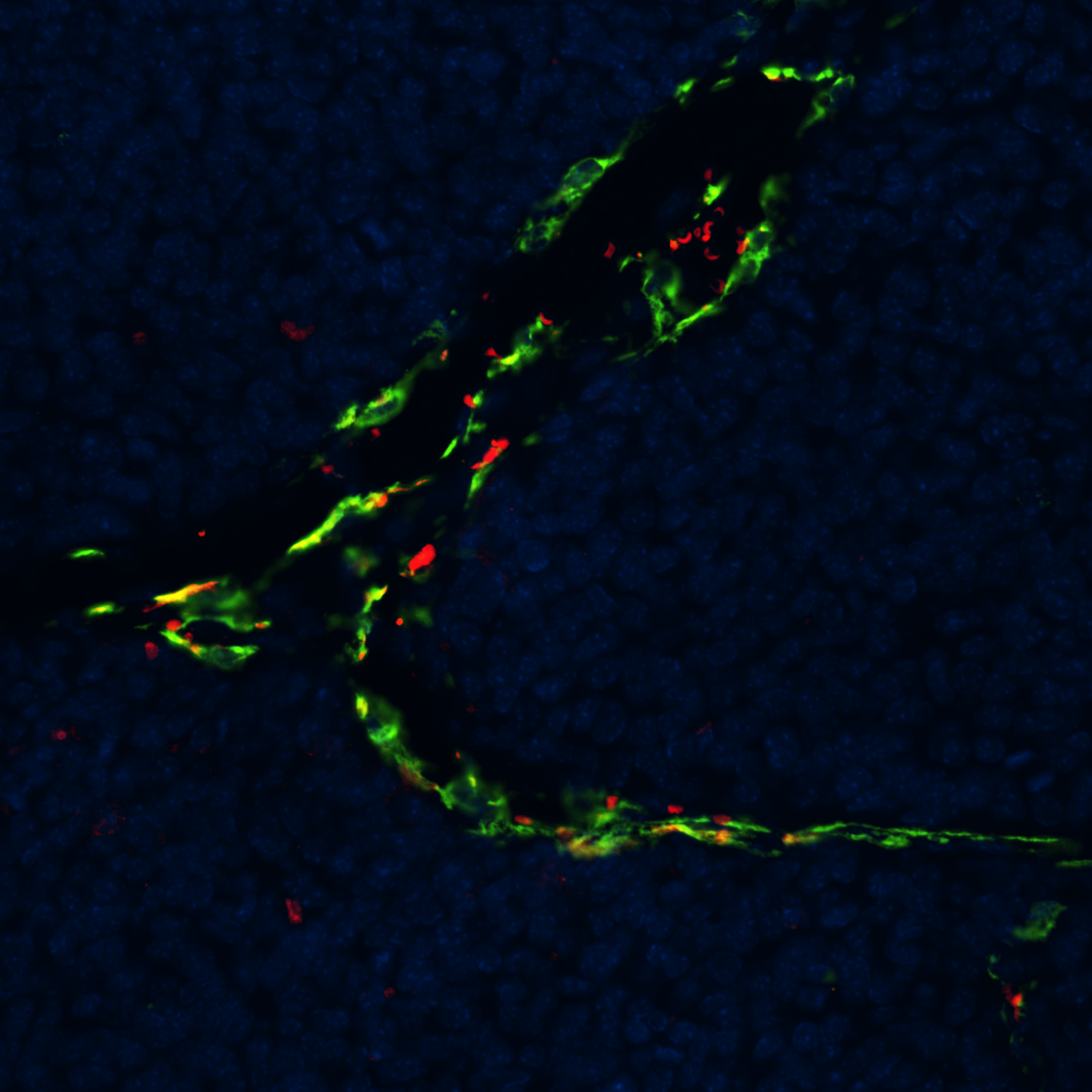

Supplement: Supplementary file 7 — Source data Fig. 5 [file 44321_2025_222_MOESM7_ESM.zip › For EMM submission/Figure 5B/AT3_Paclitaxel.tif]

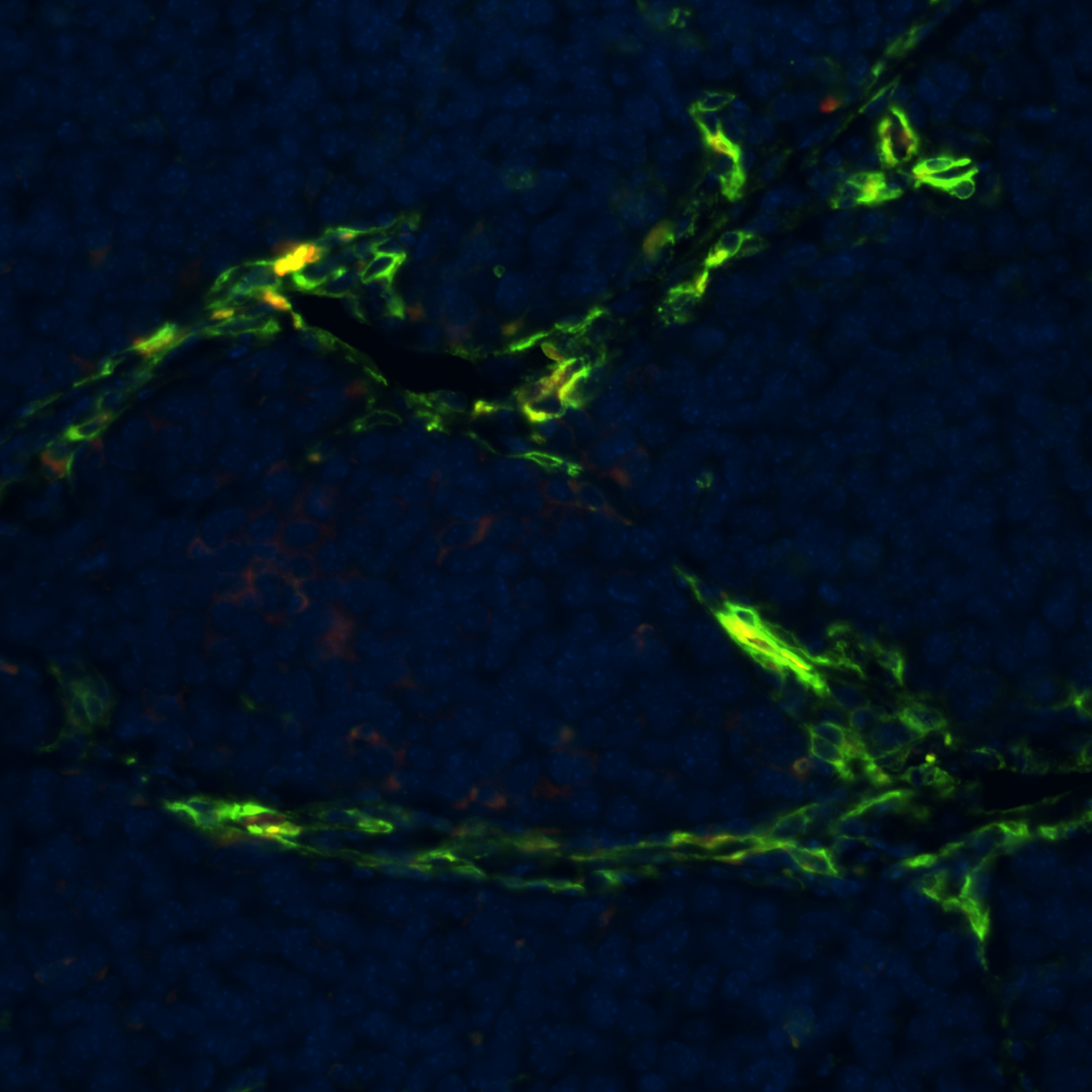

Supplement: Supplementary file 7 — Source data Fig. 5 [file 44321_2025_222_MOESM7_ESM.zip › For EMM submission/Figure 5B/AT3_Untreated.tif]

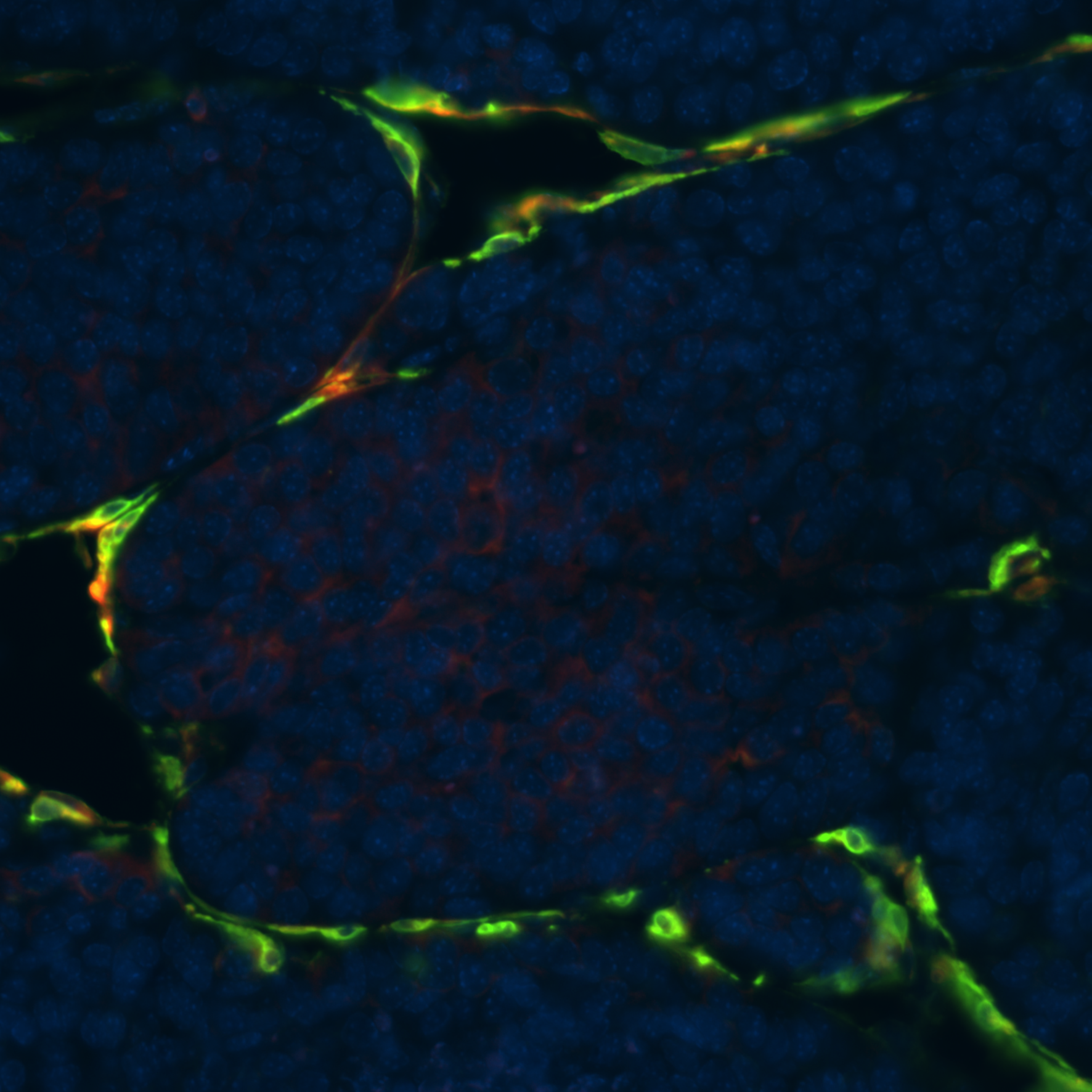

Supplement: Supplementary file 7 — Source data Fig. 5 [file 44321_2025_222_MOESM7_ESM.zip › For EMM submission/Figure 5B/AT3_Vinorelbine.tif]

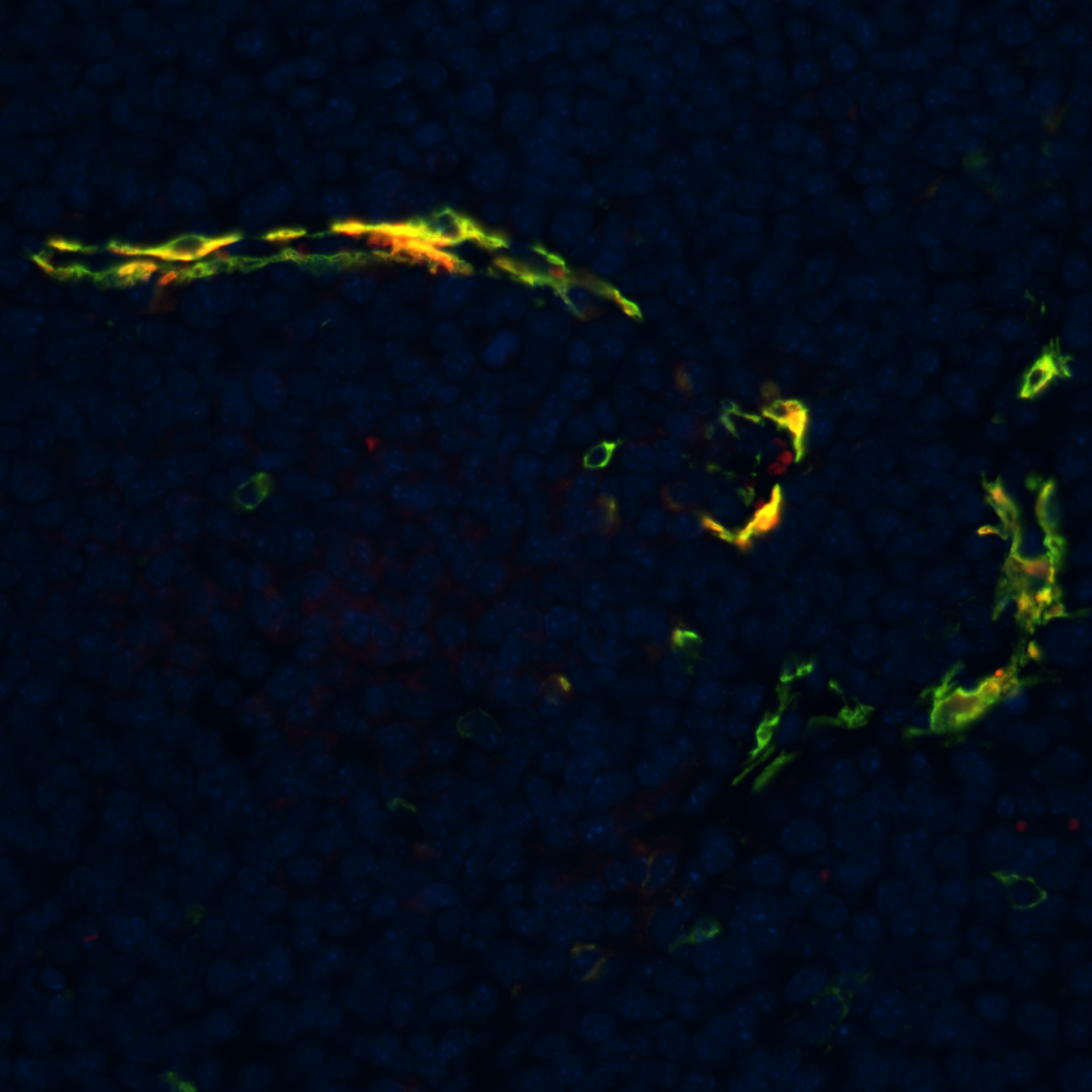

Supplement: Supplementary file 7 — Source data Fig. 5 [file 44321_2025_222_MOESM7_ESM.zip › For EMM submission/Figure 5C/AT3_CA4.tif]

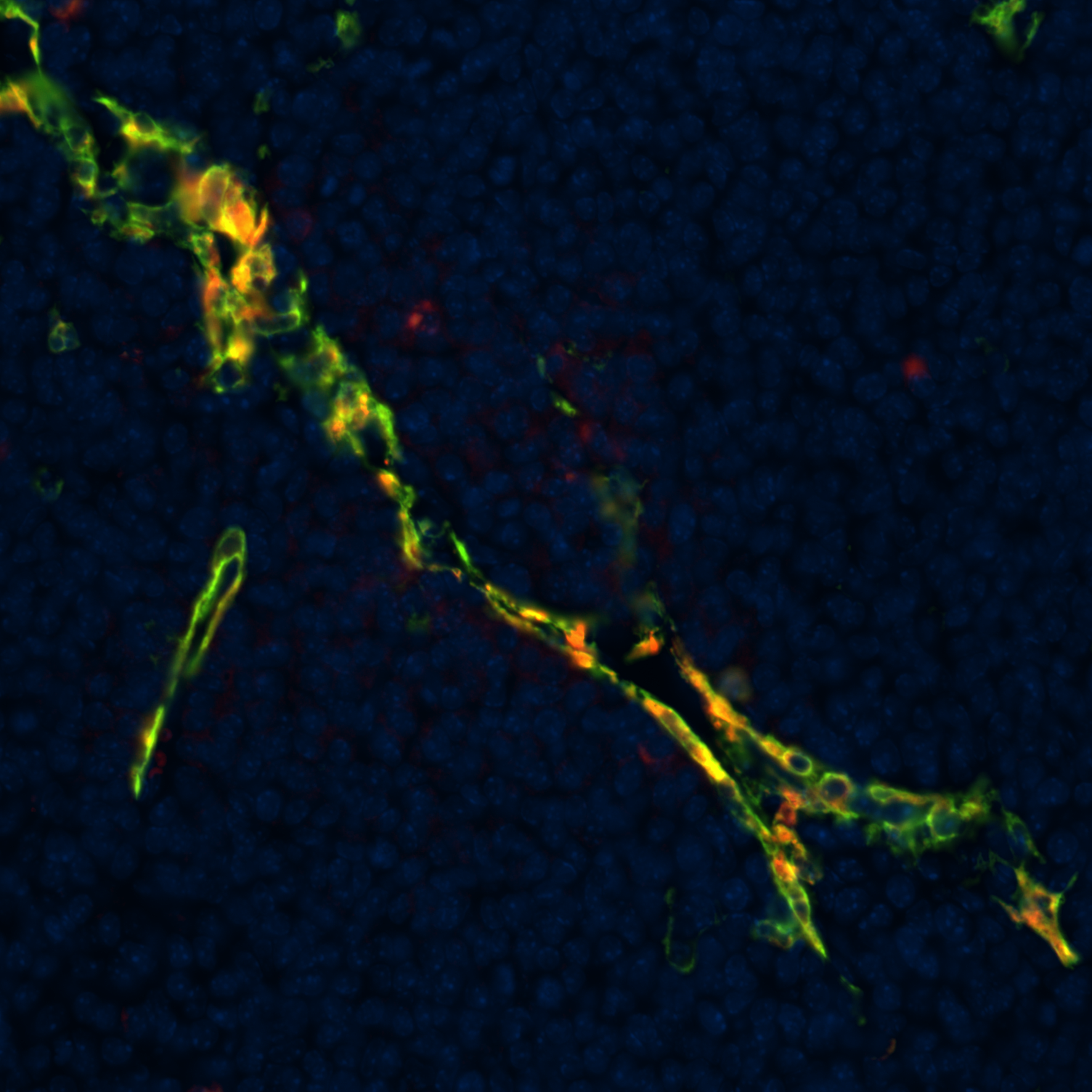

Supplement: Supplementary file 7 — Source data Fig. 5 [file 44321_2025_222_MOESM7_ESM.zip › For EMM submission/Figure 5C/AT3_Eribulin.tif]

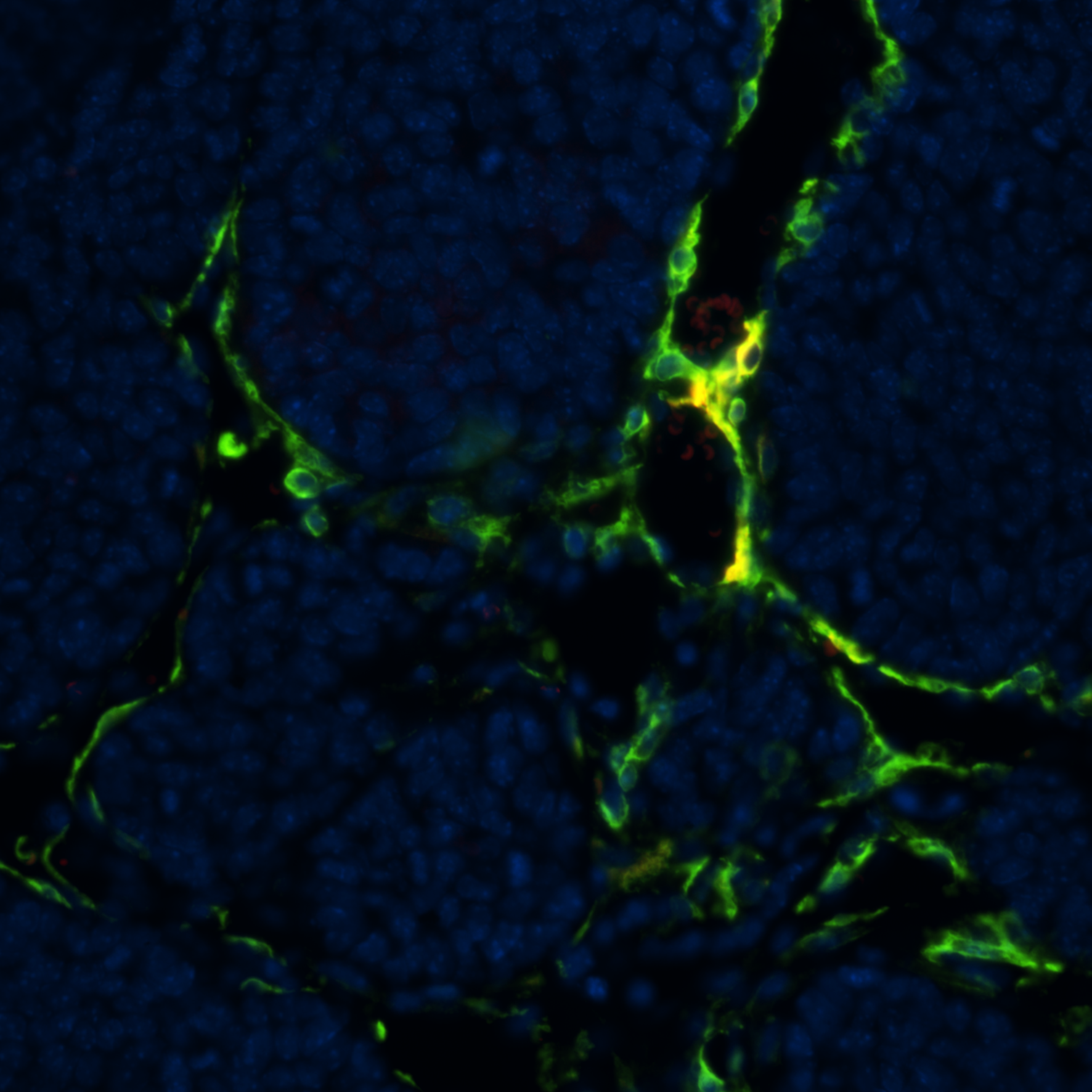

Supplement: Supplementary file 7 — Source data Fig. 5 [file 44321_2025_222_MOESM7_ESM.zip › For EMM submission/Figure 5C/AT3_Paclitaxel.tif]

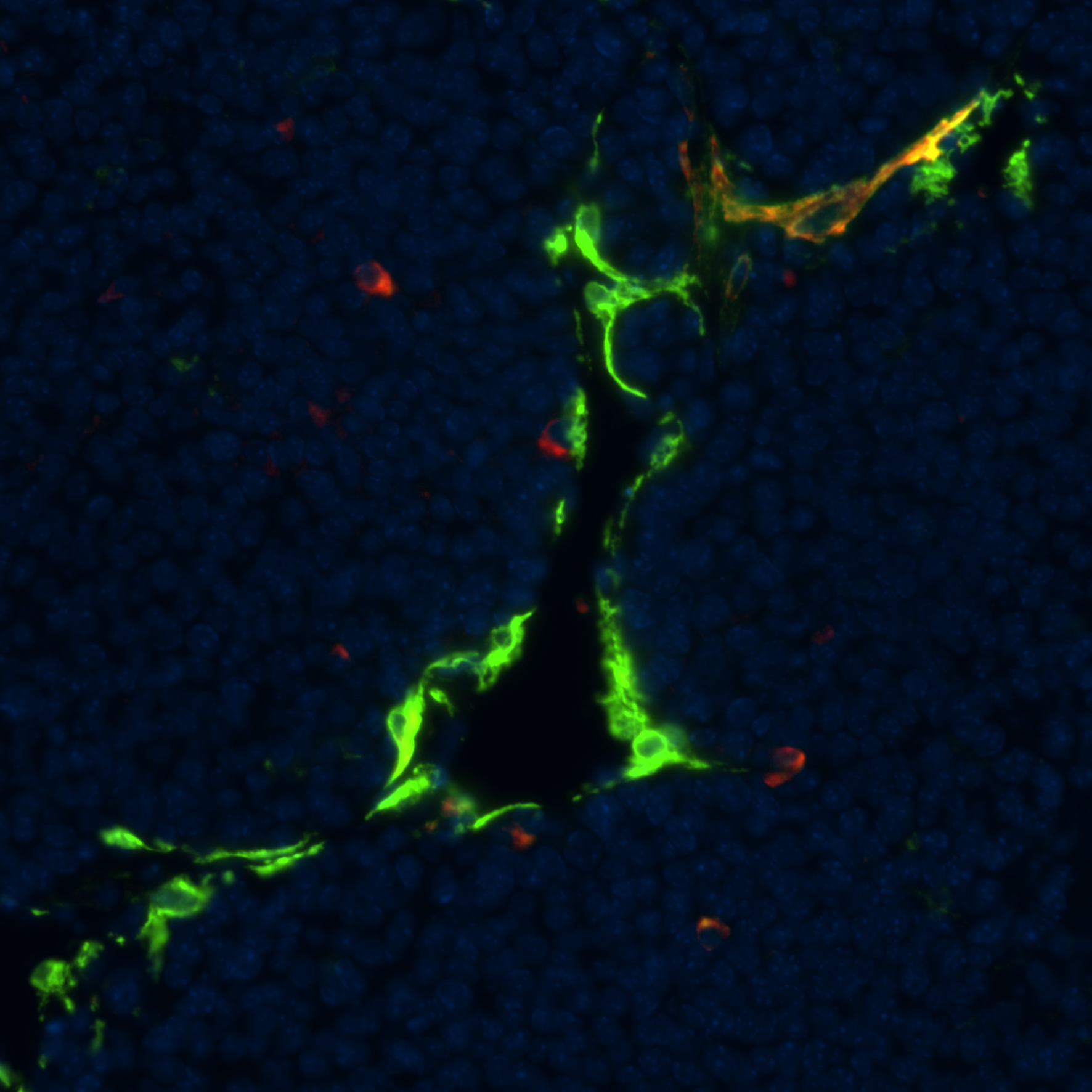

Supplement: Supplementary file 7 — Source data Fig. 5 [file 44321_2025_222_MOESM7_ESM.zip › For EMM submission/Figure 5C/AT3_Untreated.tif]

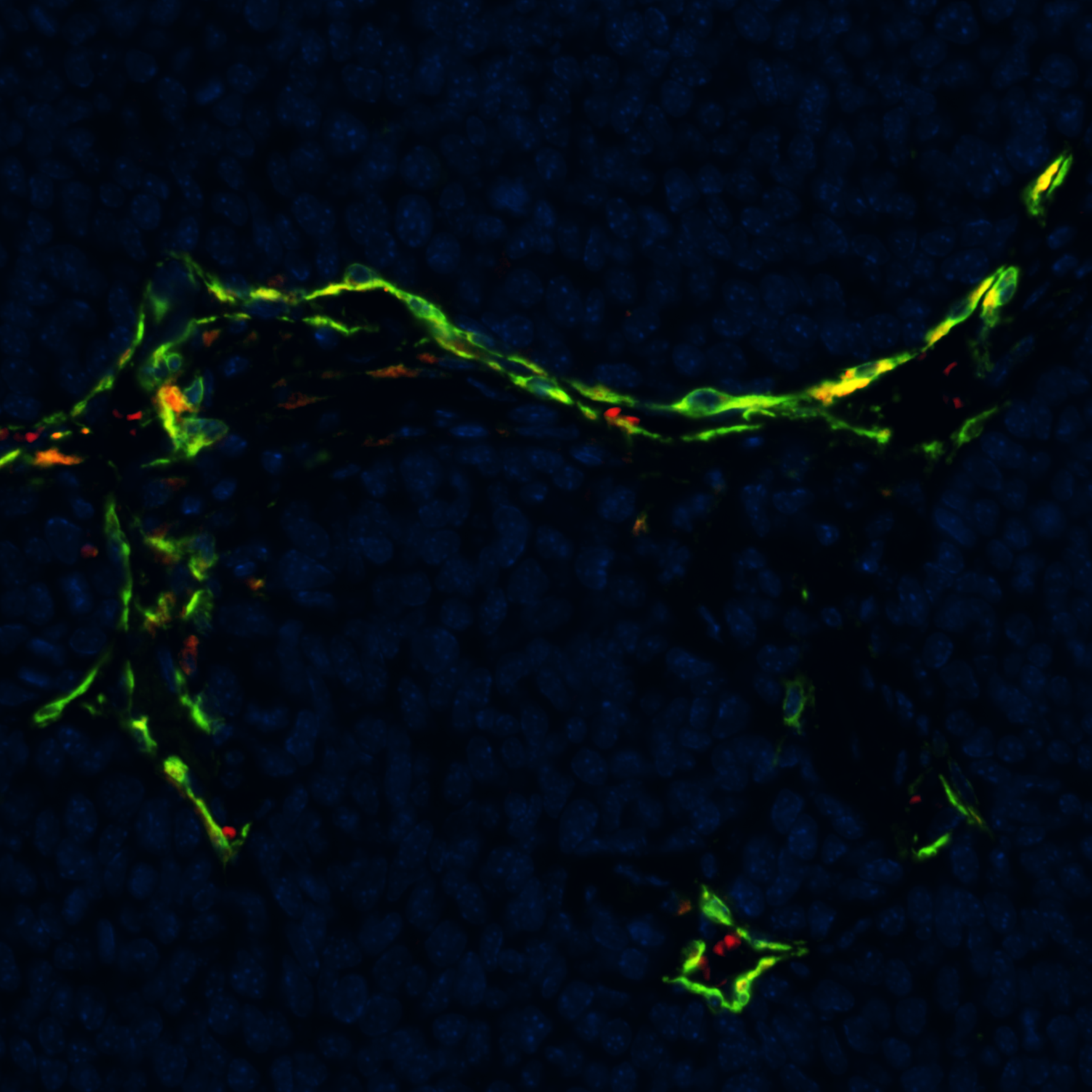

Supplement: Supplementary file 7 — Source data Fig. 5 [file 44321_2025_222_MOESM7_ESM.zip › For EMM submission/Figure 5C/AT3_Vinorelbine.tif]

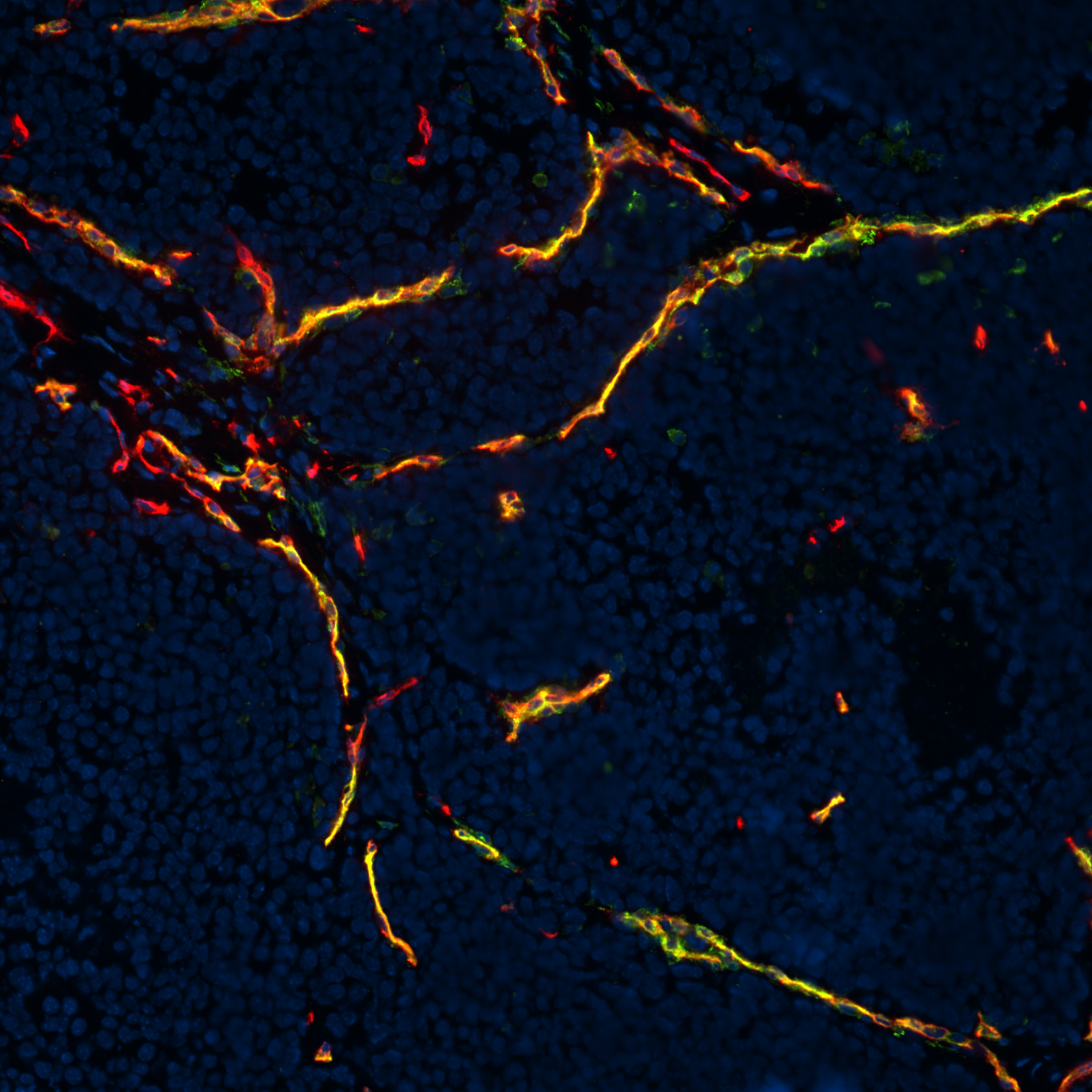

Supplement: Supplementary file 7 — Source data Fig. 5 [file 44321_2025_222_MOESM7_ESM.zip › For EMM submission/Figure 5D/AT3_CA4.tif]

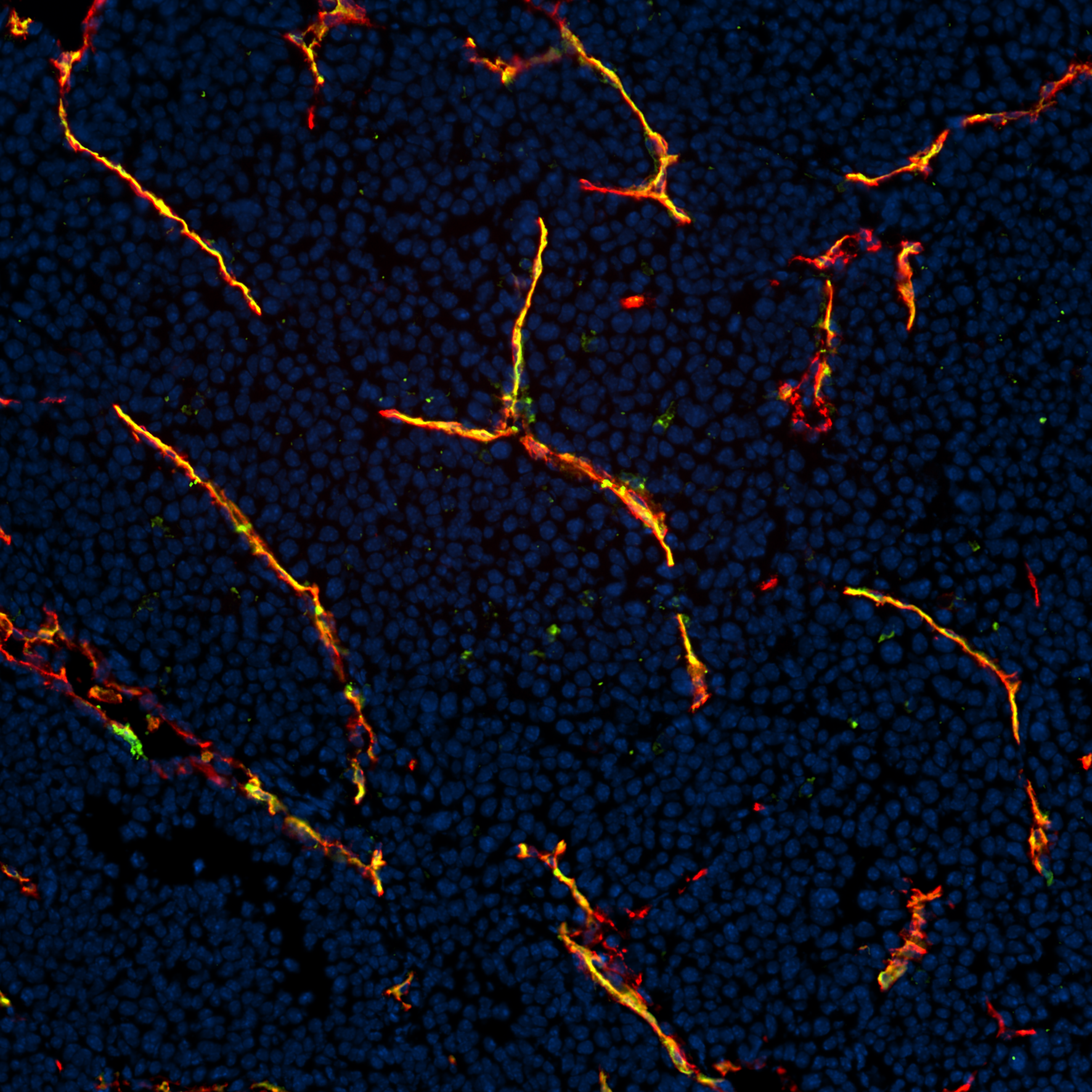

Supplement: Supplementary file 7 — Source data Fig. 5 [file 44321_2025_222_MOESM7_ESM.zip › For EMM submission/Figure 5D/AT3_Eribulin.tif]

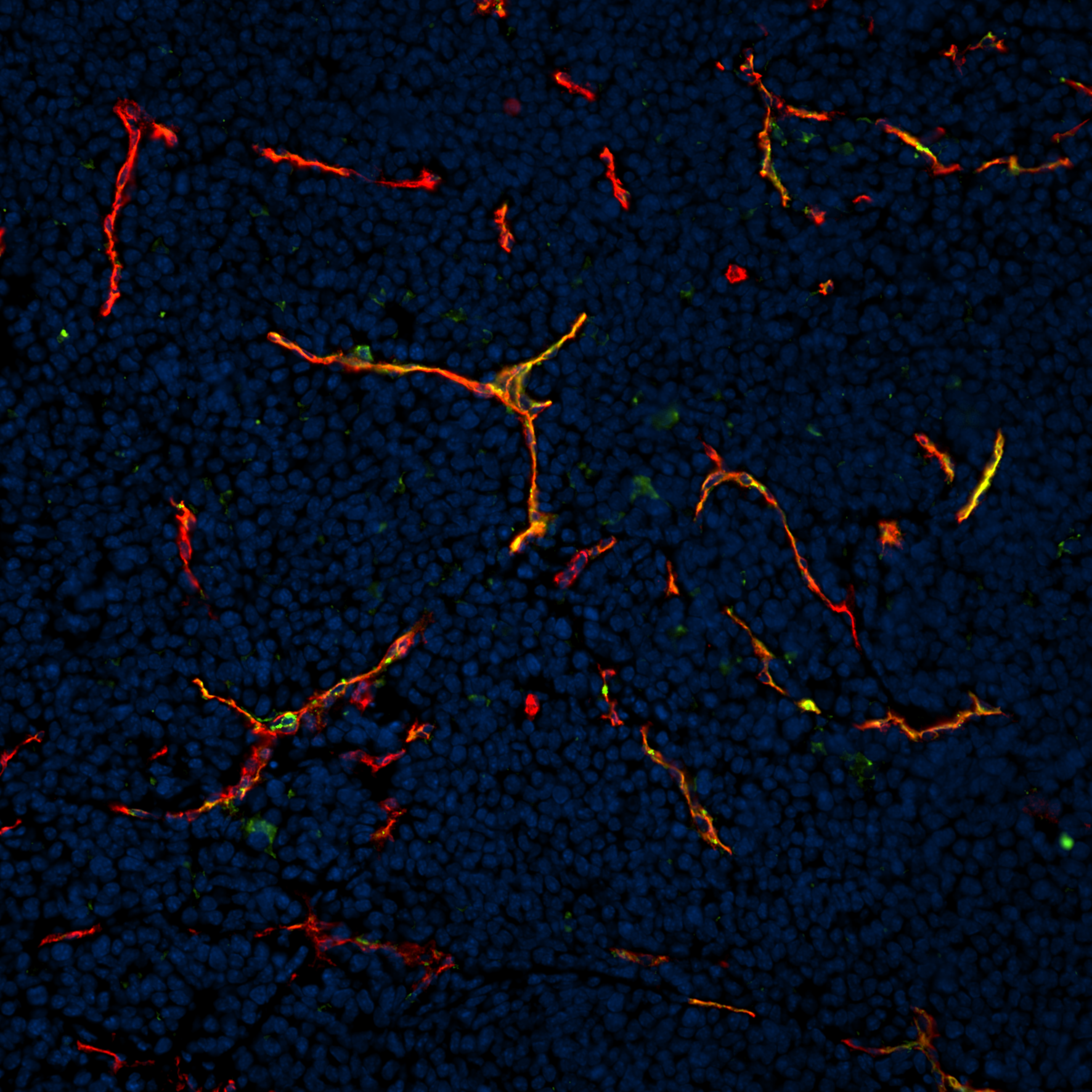

Supplement: Supplementary file 7 — Source data Fig. 5 [file 44321_2025_222_MOESM7_ESM.zip › For EMM submission/Figure 5D/AT3_Paclitaxel.tif]

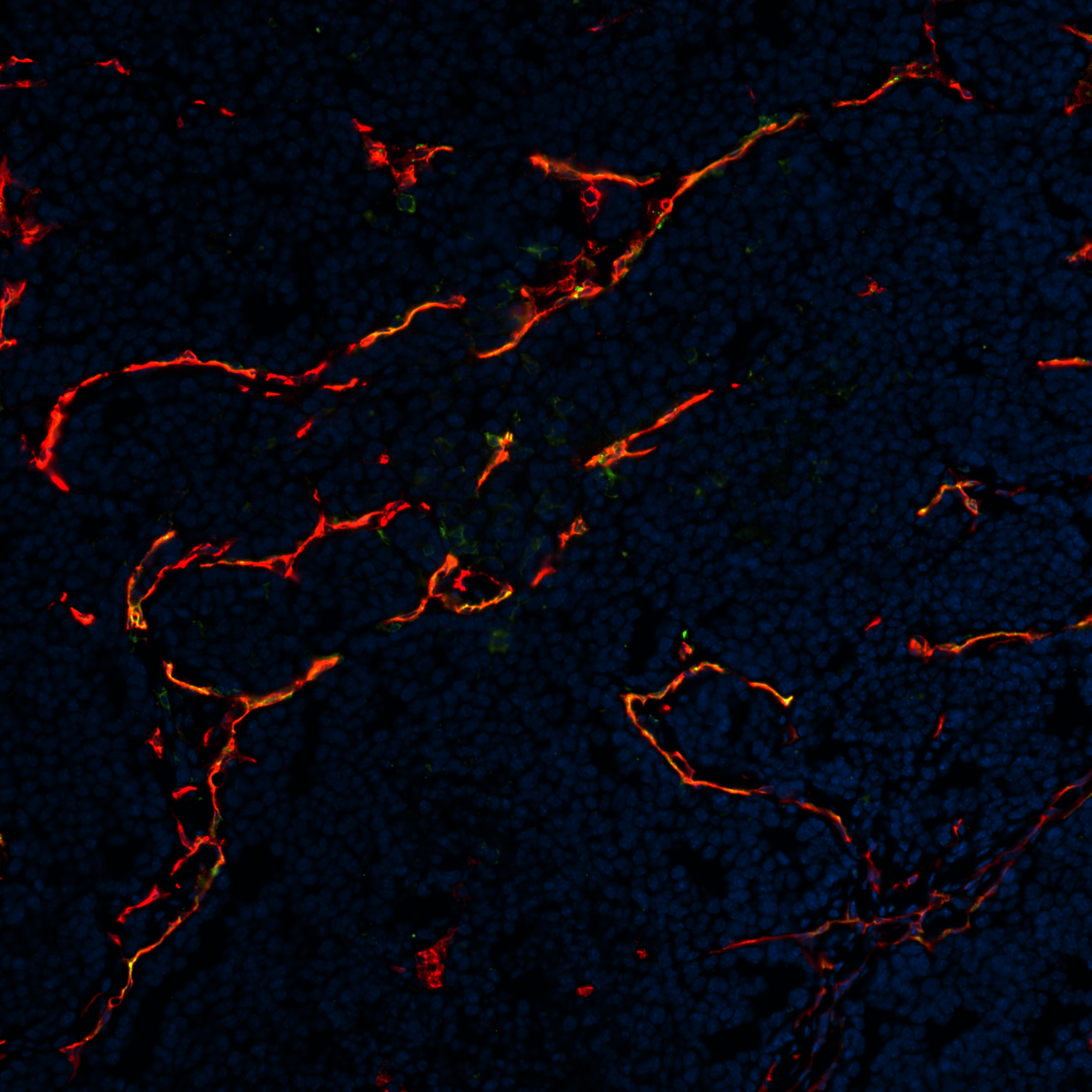

Supplement: Supplementary file 7 — Source data Fig. 5 [file 44321_2025_222_MOESM7_ESM.zip › For EMM submission/Figure 5D/AT3_Untreated.tif]

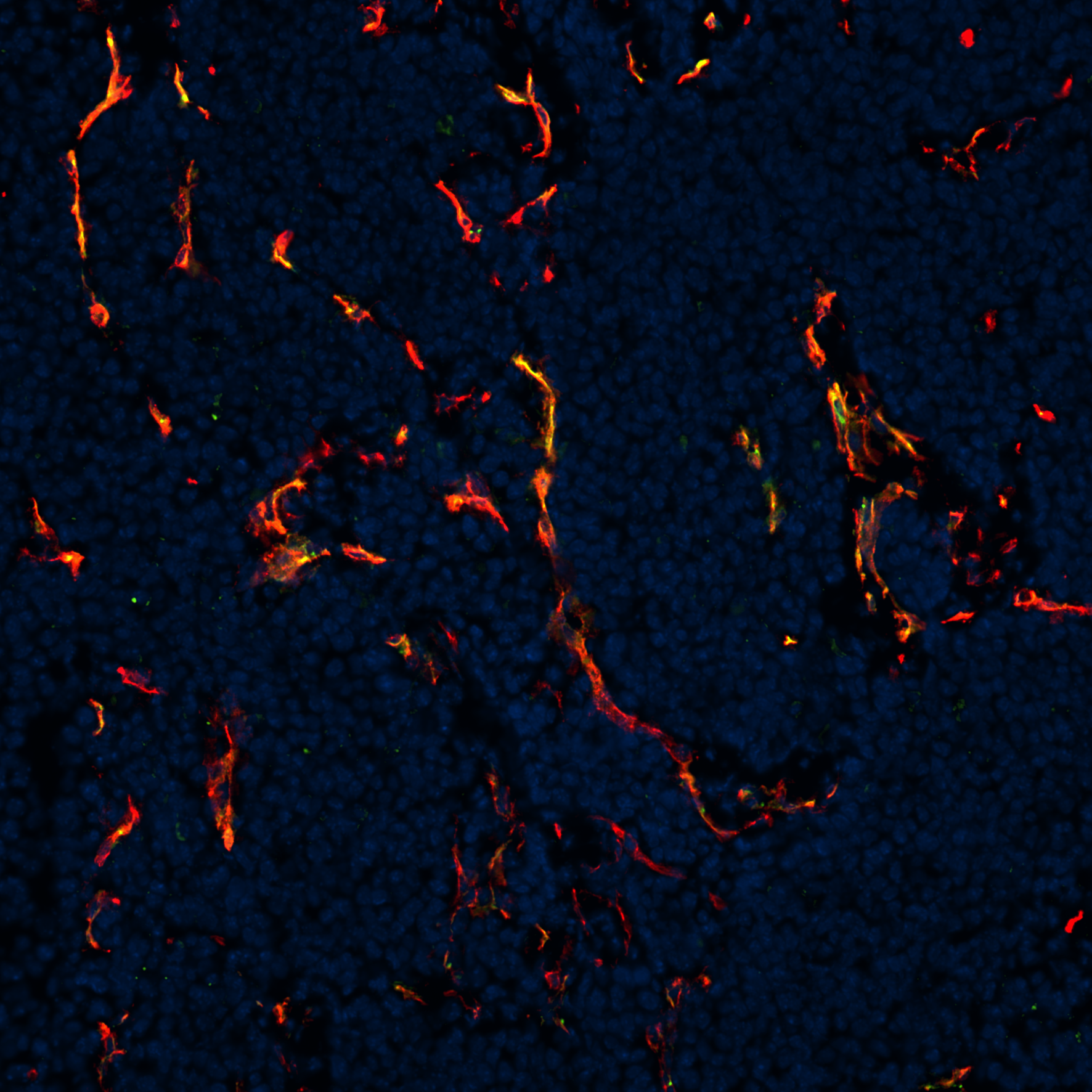

Supplement: Supplementary file 7 — Source data Fig. 5 [file 44321_2025_222_MOESM7_ESM.zip › For EMM submission/Figure 5D/AT3_Vinorelbine.tif]

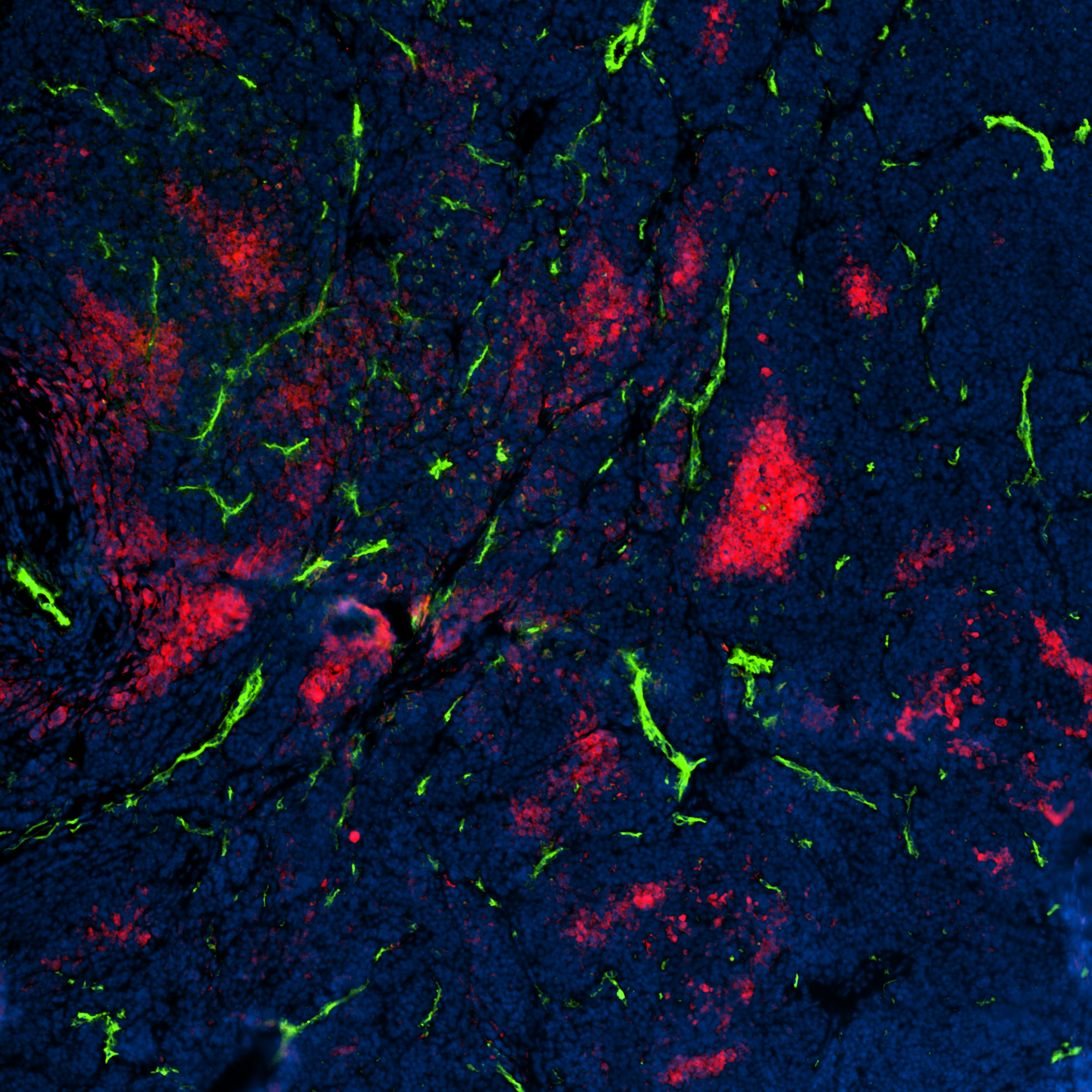

Supplement: Supplementary file 8 — Source data Fig. 6 [file 44321_2025_222_MOESM8_ESM.zip › For EMM submission/Figure 6A/Hypoxia/AT3 _ Eribulin.tif]

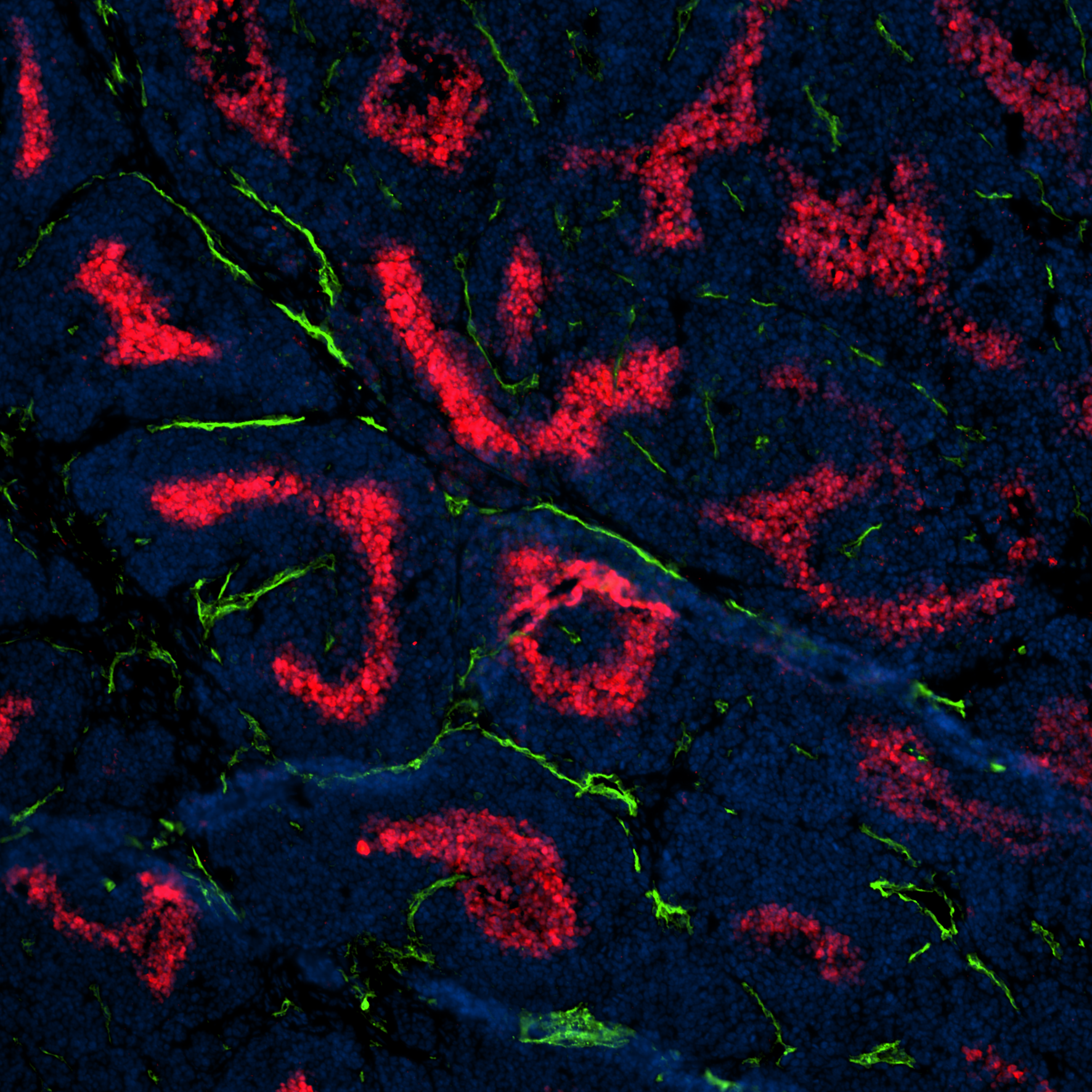

Supplement: Supplementary file 8 — Source data Fig. 6 [file 44321_2025_222_MOESM8_ESM.zip › For EMM submission/Figure 6A/Hypoxia/AT3 _ Untr.tif]

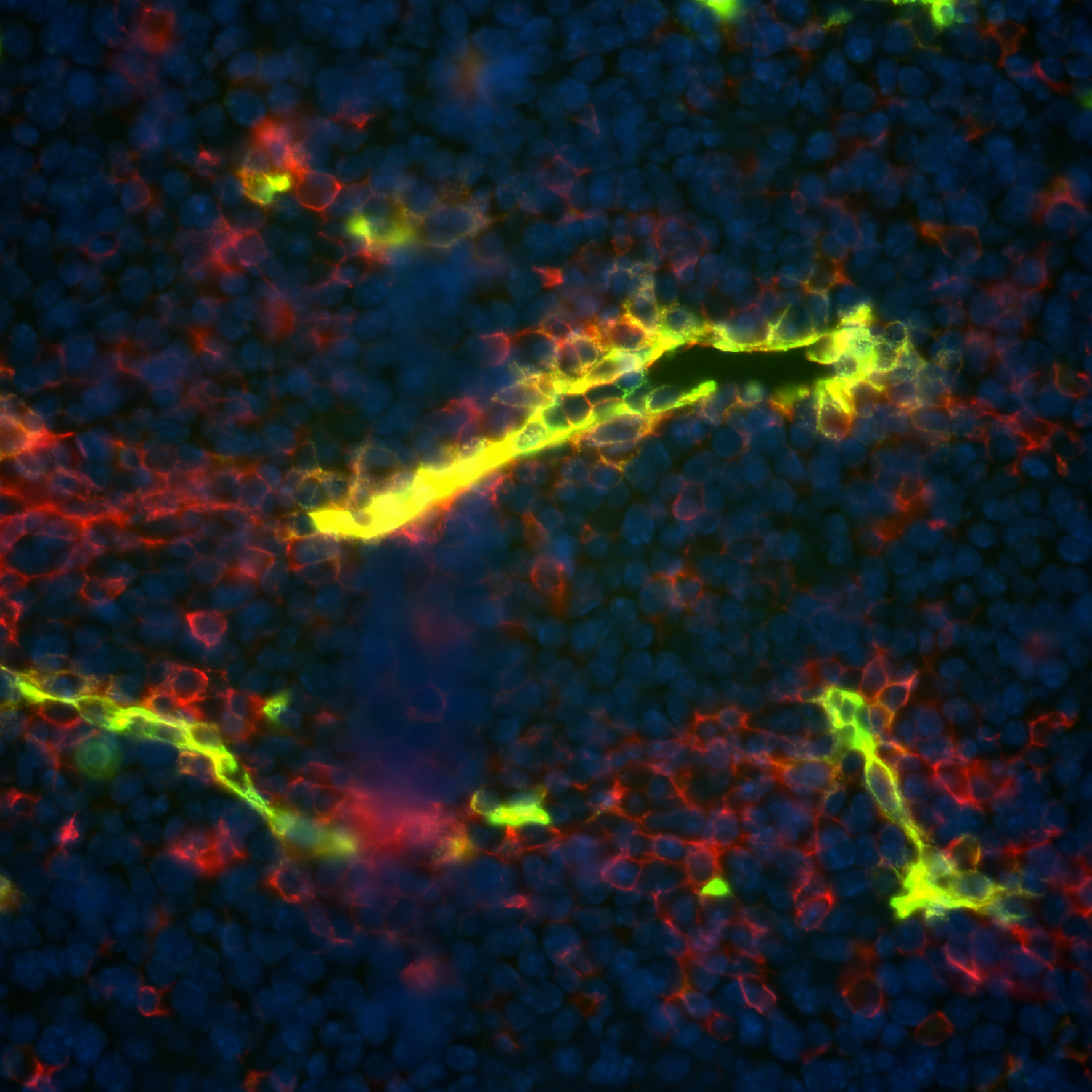

Supplement: Supplementary file 8 — Source data Fig. 6 [file 44321_2025_222_MOESM8_ESM.zip › For EMM submission/Figure 6A/ICAM/AT3_eribulin.tif]

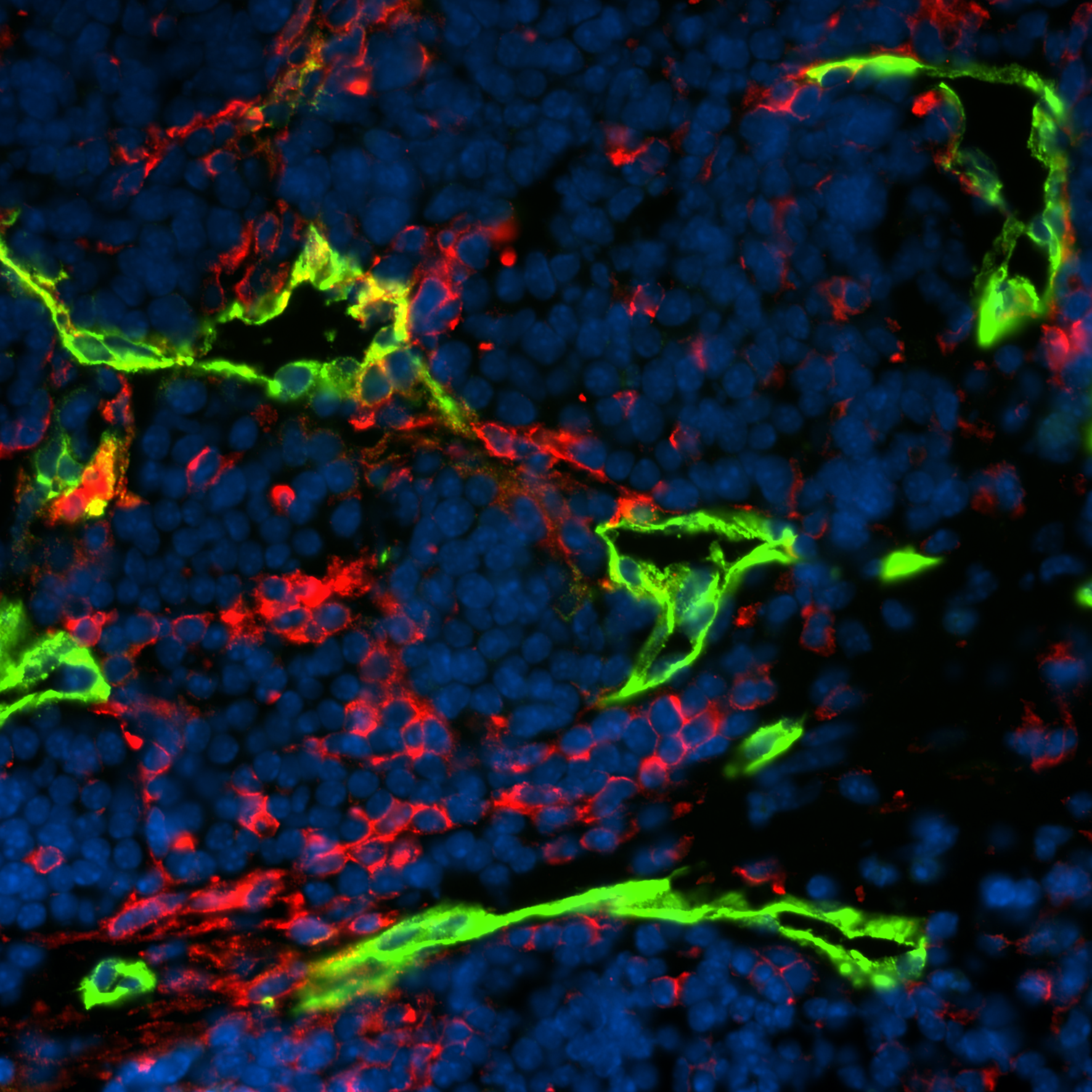

Supplement: Supplementary file 8 — Source data Fig. 6 [file 44321_2025_222_MOESM8_ESM.zip › For EMM submission/Figure 6A/ICAM/AT3_Untreated.tif]

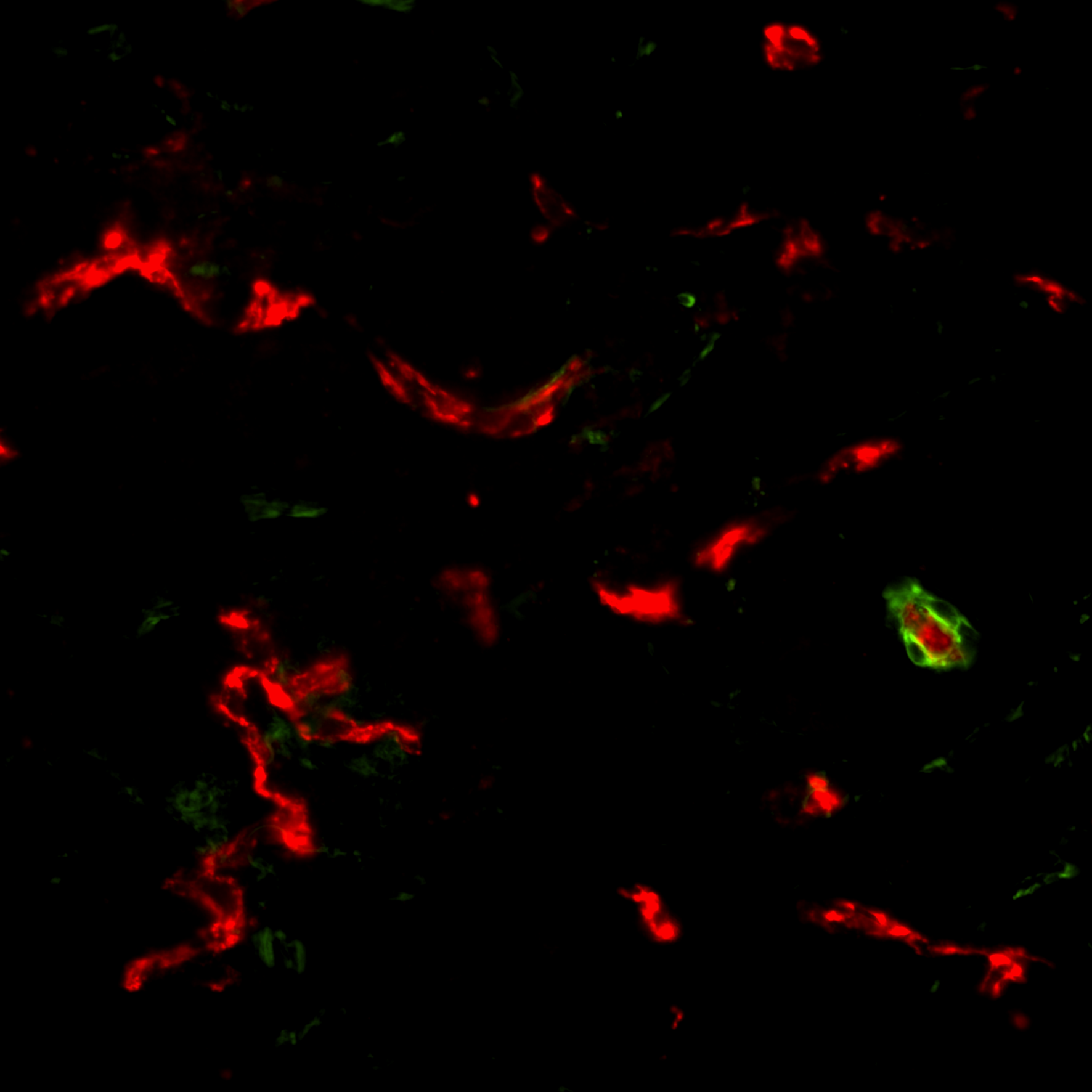

Supplement: Supplementary file 10 — Source data Fig. 8 [file 44321_2025_222_MOESM10_ESM.zip › For EMM submission/Figure 8A/CD31 - aSMA/SOLTI 1 cycle eribulin.tif]

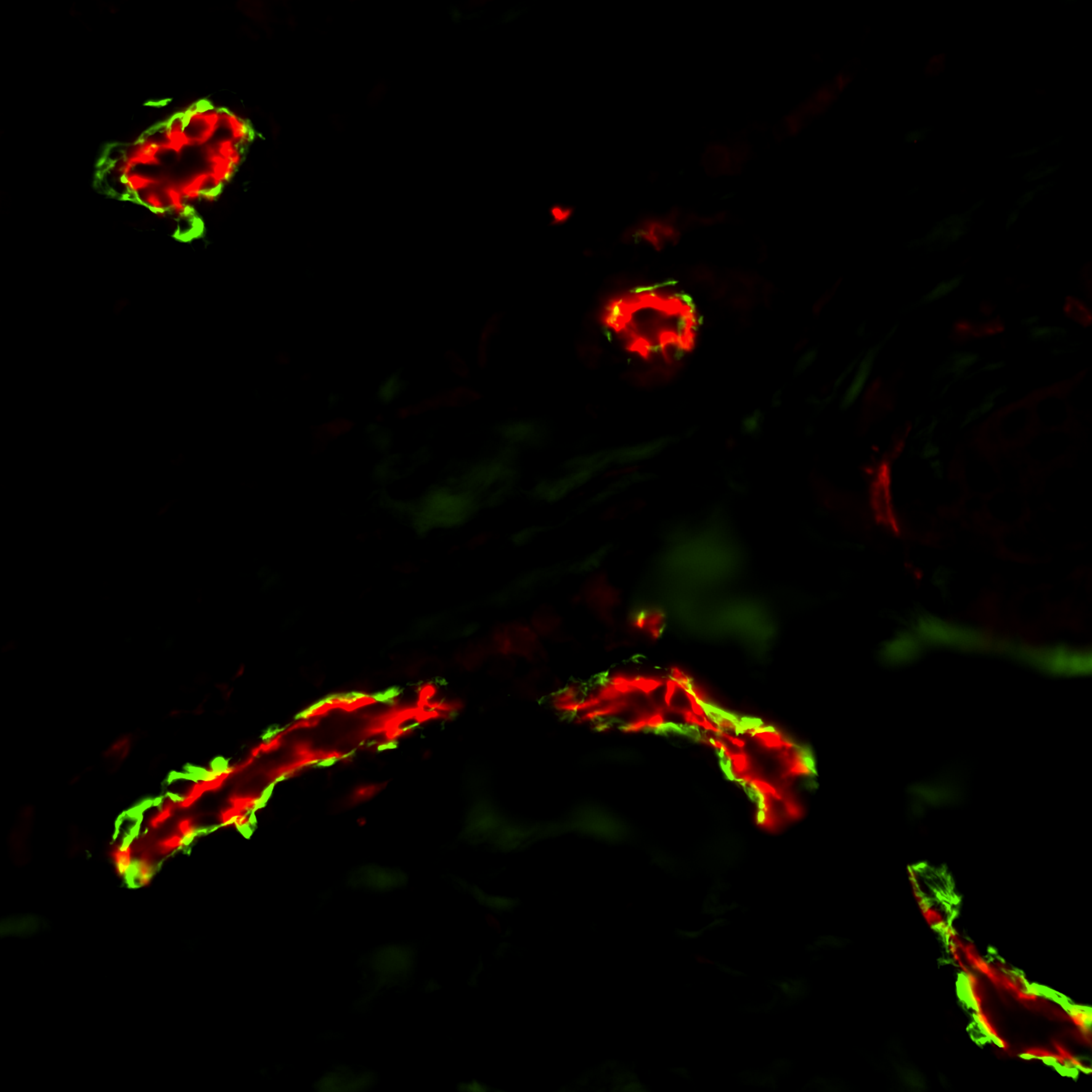

Supplement: Supplementary file 10 — Source data Fig. 8 [file 44321_2025_222_MOESM10_ESM.zip › For EMM submission/Figure 8A/CD31 - aSMA/SOLTI 4 cycles eribulin.tif]

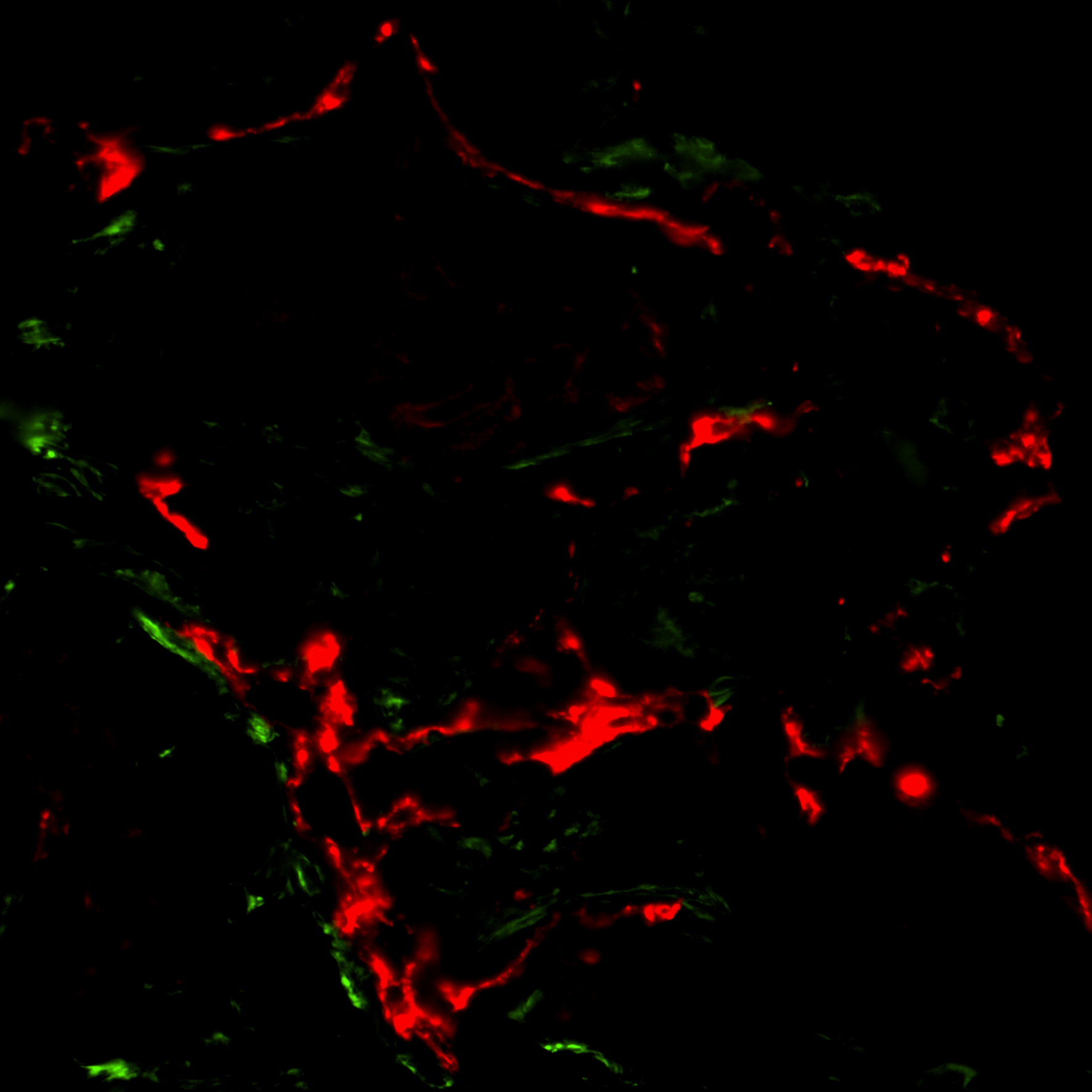

Supplement: Supplementary file 10 — Source data Fig. 8 [file 44321_2025_222_MOESM10_ESM.zip › For EMM submission/Figure 8A/CD31 - aSMA/SOLTI pre-treatment.tif]

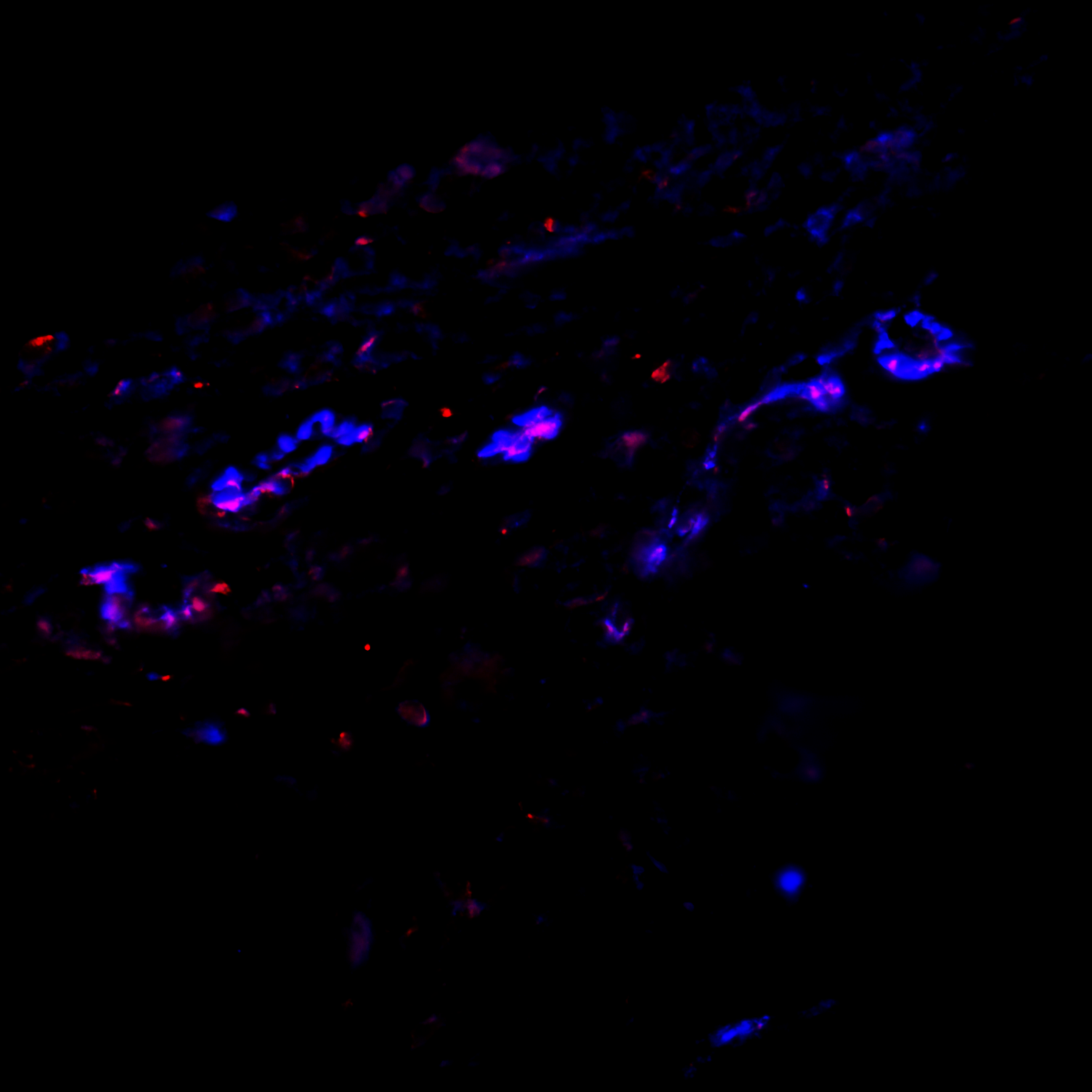

Supplement: Supplementary file 10 — Source data Fig. 8 [file 44321_2025_222_MOESM10_ESM.zip › For EMM submission/Figure 8A/CD31 - CDH5/SOLTI 1 cycle eribulin.tif]

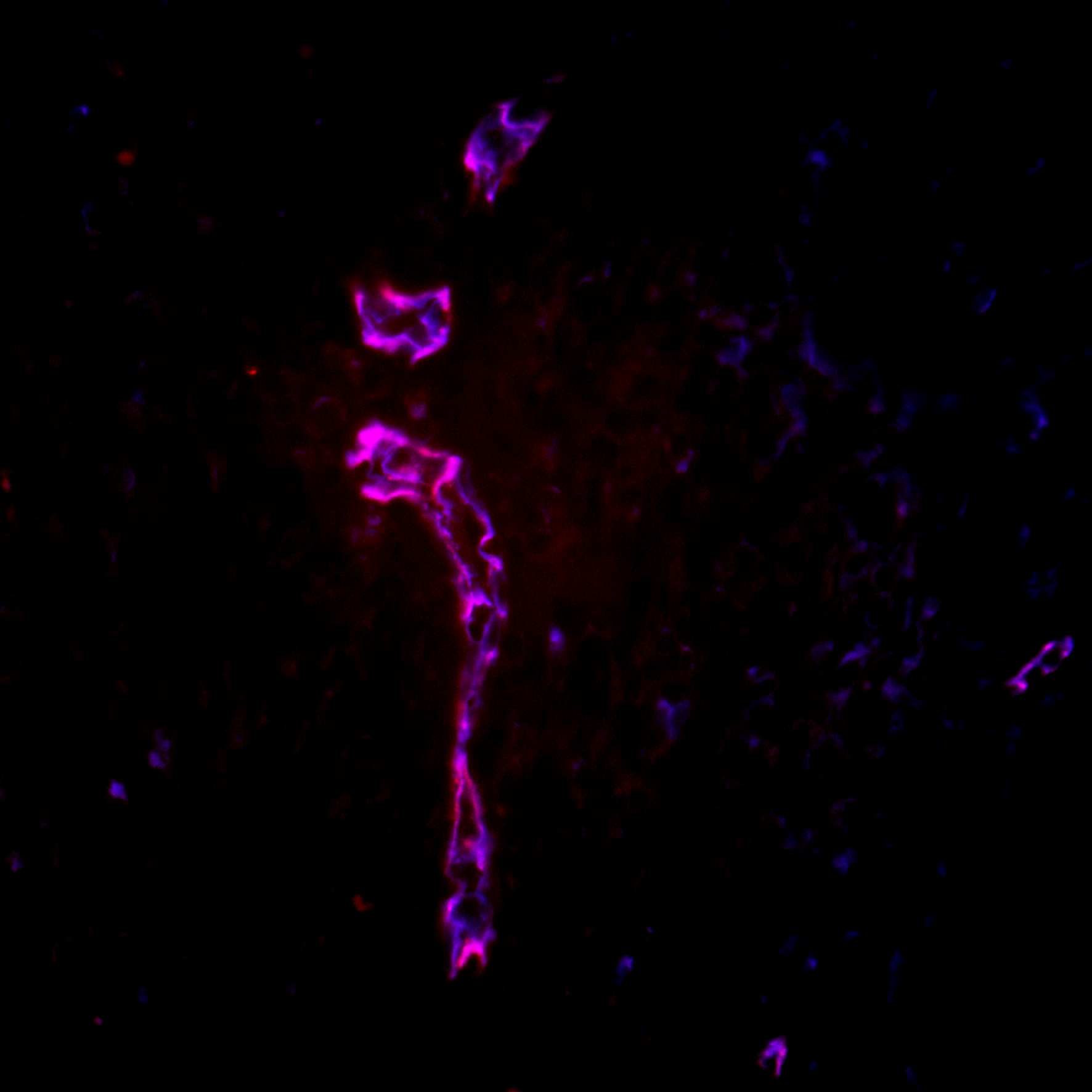

Supplement: Supplementary file 10 — Source data Fig. 8 [file 44321_2025_222_MOESM10_ESM.zip › For EMM submission/Figure 8A/CD31 - CDH5/SOLTI 4 cycles eribulin.tif]

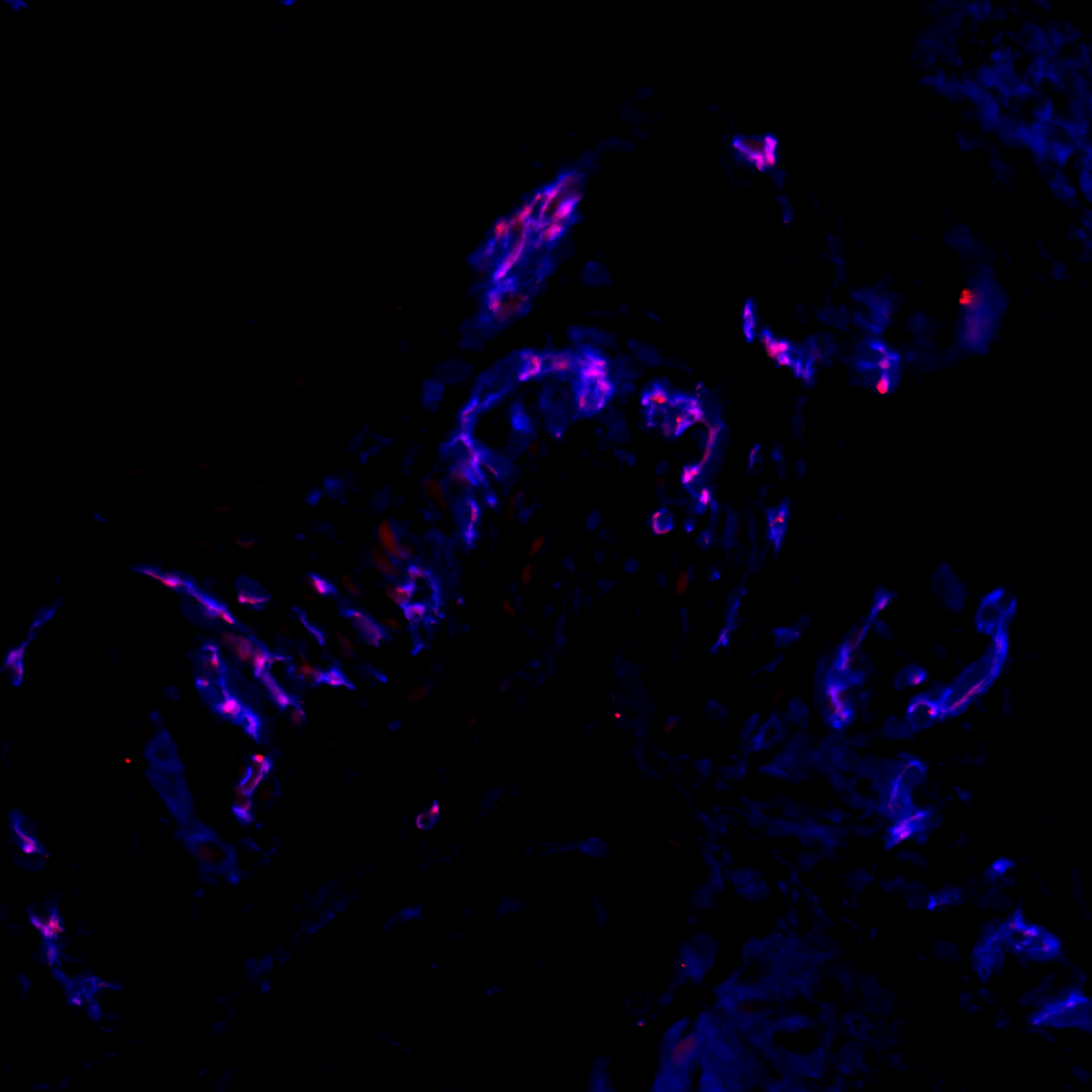

Supplement: Supplementary file 10 — Source data Fig. 8 [file 44321_2025_222_MOESM10_ESM.zip › For EMM submission/Figure 8A/CD31 - CDH5/SOLTI pre-treatment.tif]

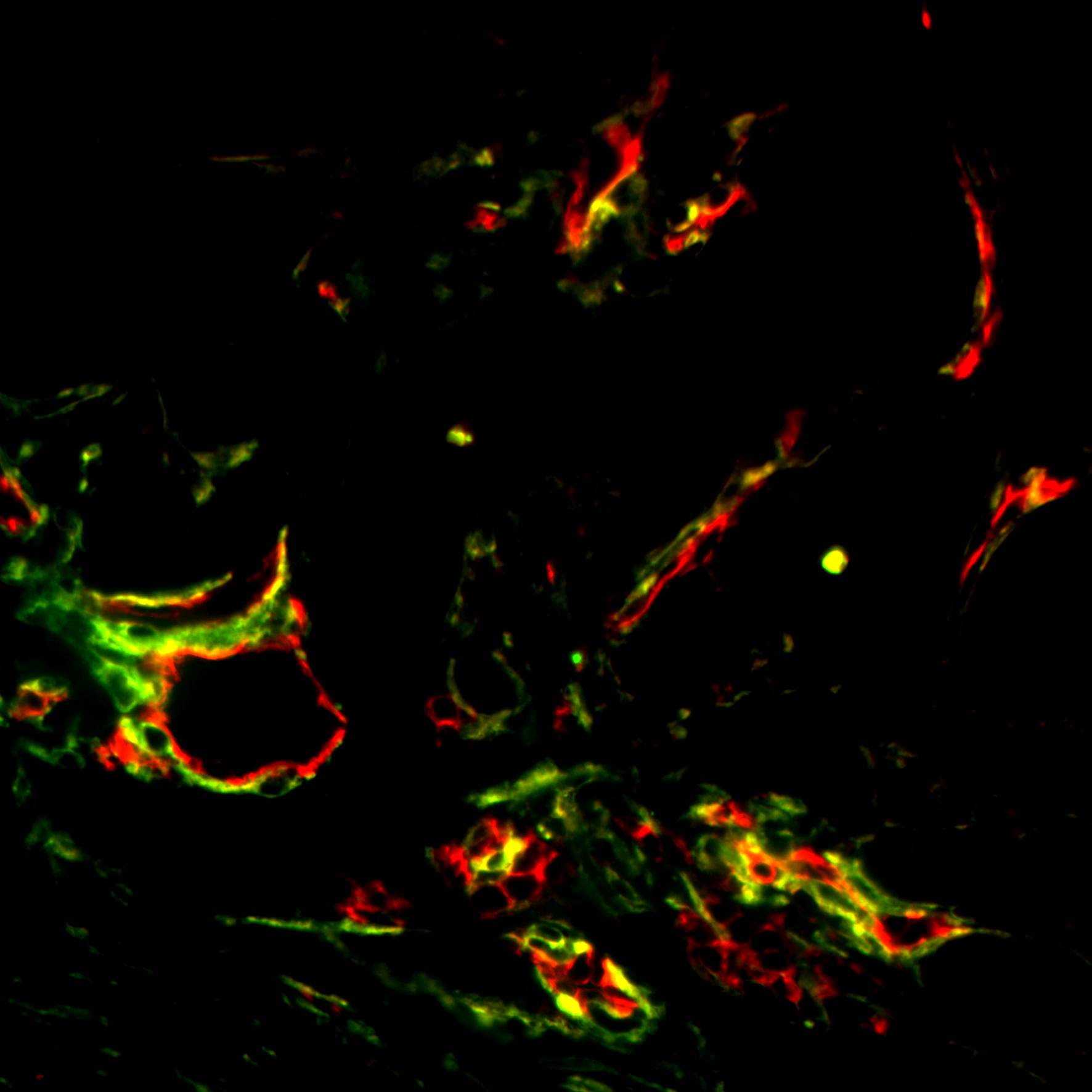

Supplement: Supplementary file 10 — Source data Fig. 8 [file 44321_2025_222_MOESM10_ESM.zip › For EMM submission/Figure 8A/CD31 - PDGFRb/SOLTI 1 cycle eribulin.tif]

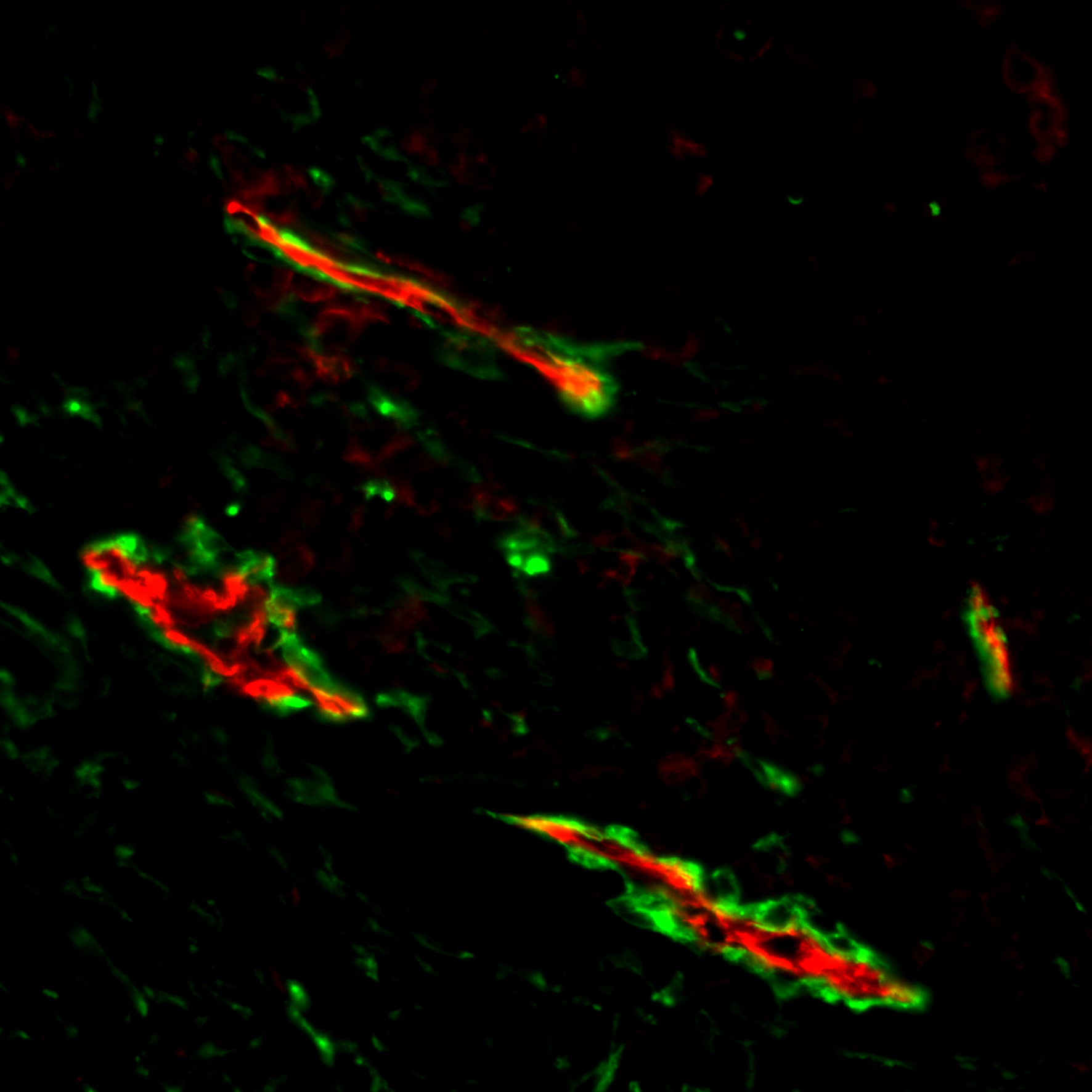

Supplement: Supplementary file 10 — Source data Fig. 8 [file 44321_2025_222_MOESM10_ESM.zip › For EMM submission/Figure 8A/CD31 - PDGFRb/SOLTI 4 cycles eribulin.tif]

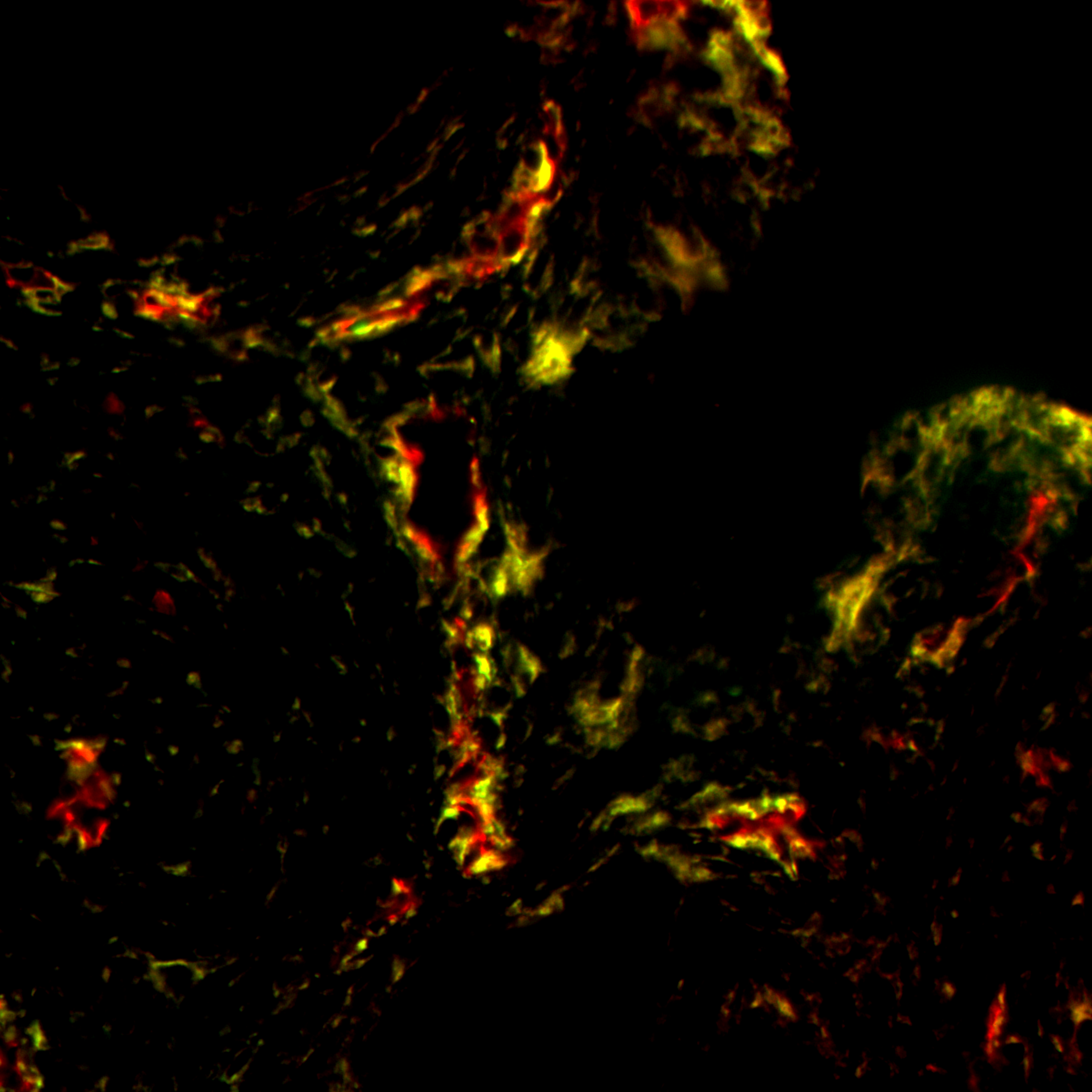

Supplement: Supplementary file 10 — Source data Fig. 8 [file 44321_2025_222_MOESM10_ESM.zip › For EMM submission/Figure 8A/CD31 - PDGFRb/SOLTI pre-treatment.tif]

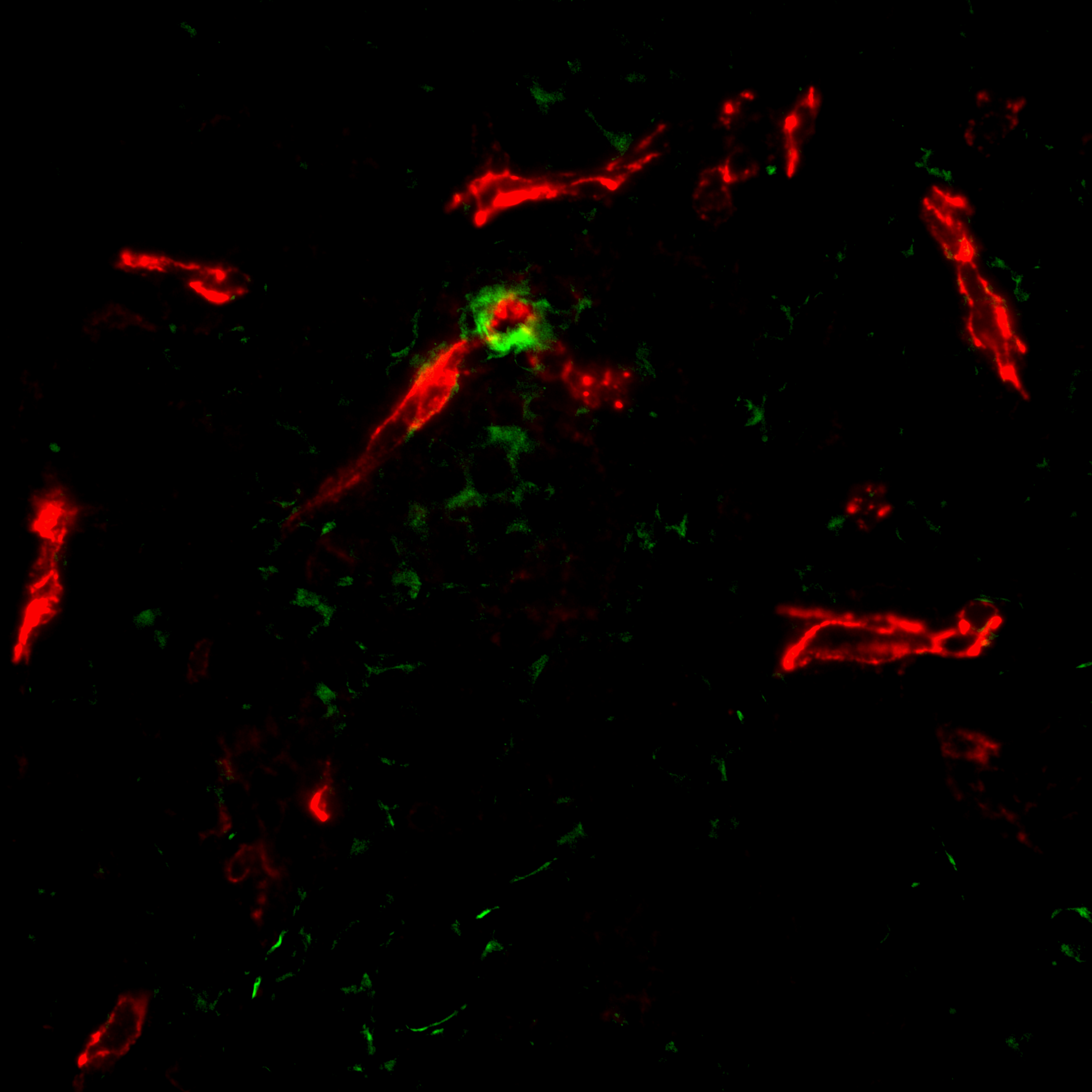

Supplement: Supplementary file 10 — Source data Fig. 8 [file 44321_2025_222_MOESM10_ESM.zip › For EMM submission/Figure 8A/CD31 - pMLC/SOLTI 1 cycle eribulin.tif]

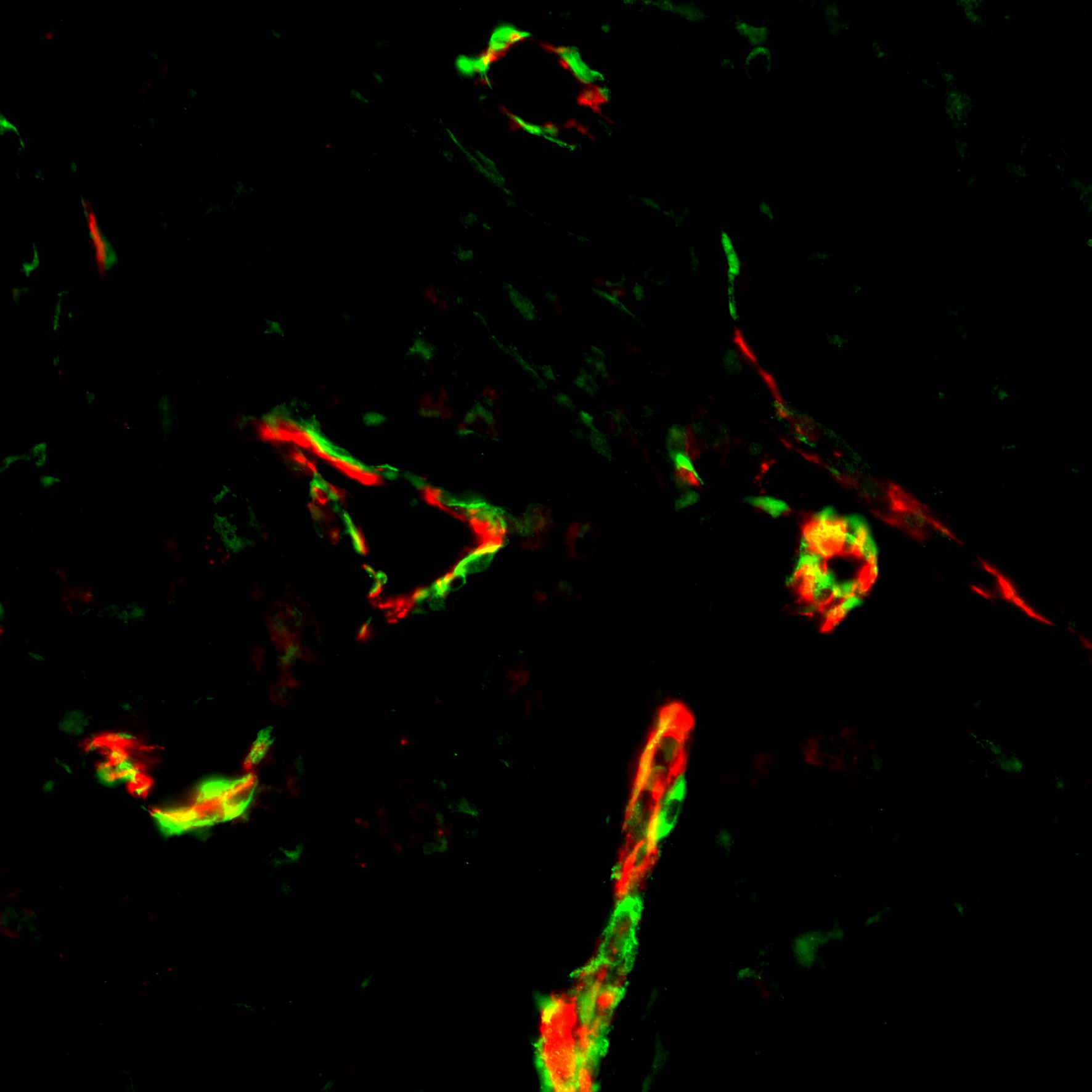

Supplement: Supplementary file 10 — Source data Fig. 8 [file 44321_2025_222_MOESM10_ESM.zip › For EMM submission/Figure 8A/CD31 - pMLC/SOLTI 4 cycles eribulin.tif]

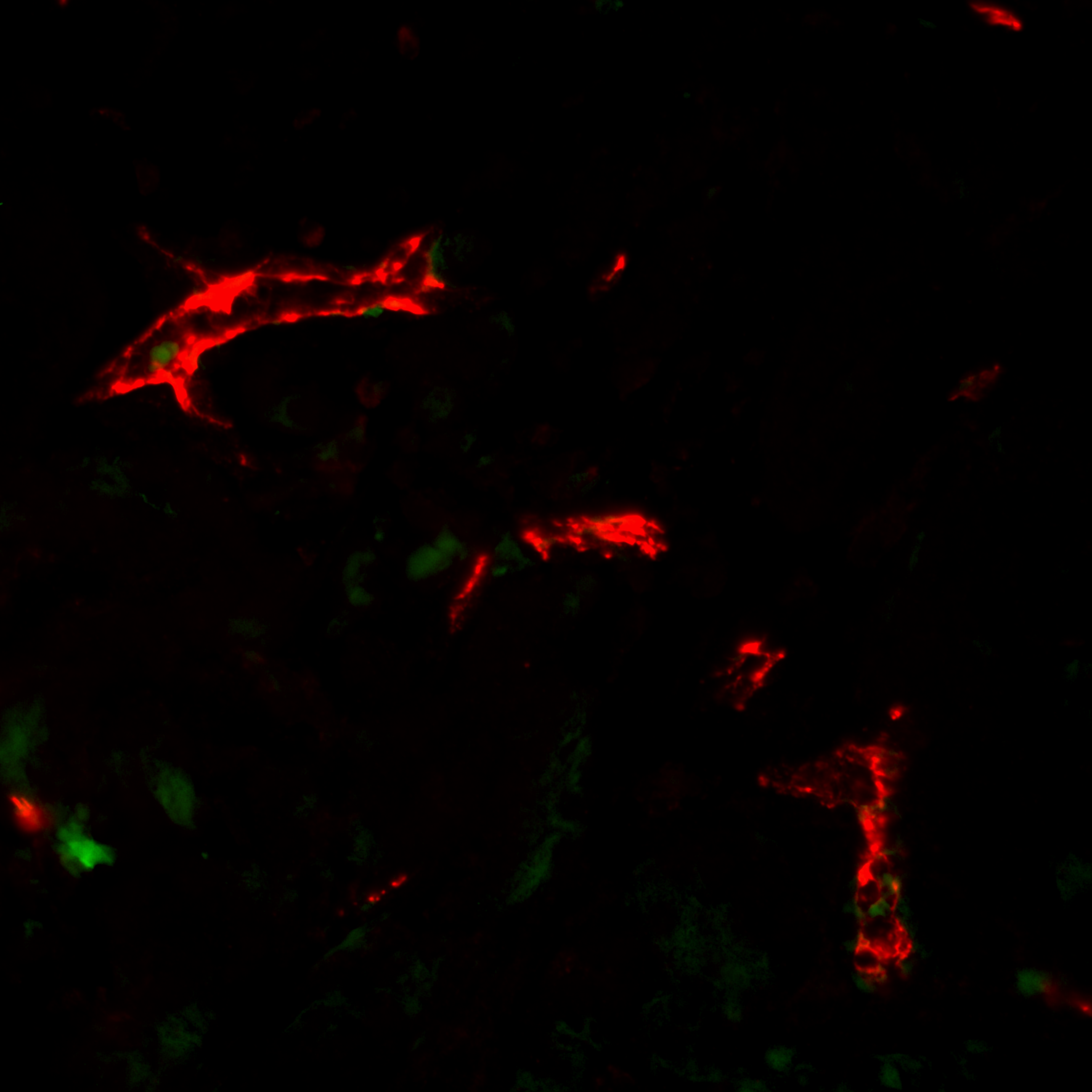

Supplement: Supplementary file 10 — Source data Fig. 8 [file 44321_2025_222_MOESM10_ESM.zip › For EMM submission/Figure 8A/CD31 - pMLC/SOLTI pre-treatment.tif]
