# Supplementary material for: Selective tubulin-binding drugs induce pericyte phenotype switching and anti-cancer immunity
Source: EMBO Mol Med. 2025 Mar 26;17(5):1071–100. doi: 10.1038/s44321-025-00222-6 (PMC12081767; doi:10.1038/s44321-025-00222-6)
Supplement: Supplementary file 11 — Expanded View Figures [file 44321_2025_222_MOESM11_ESM.pdf]

Expanded View Figures

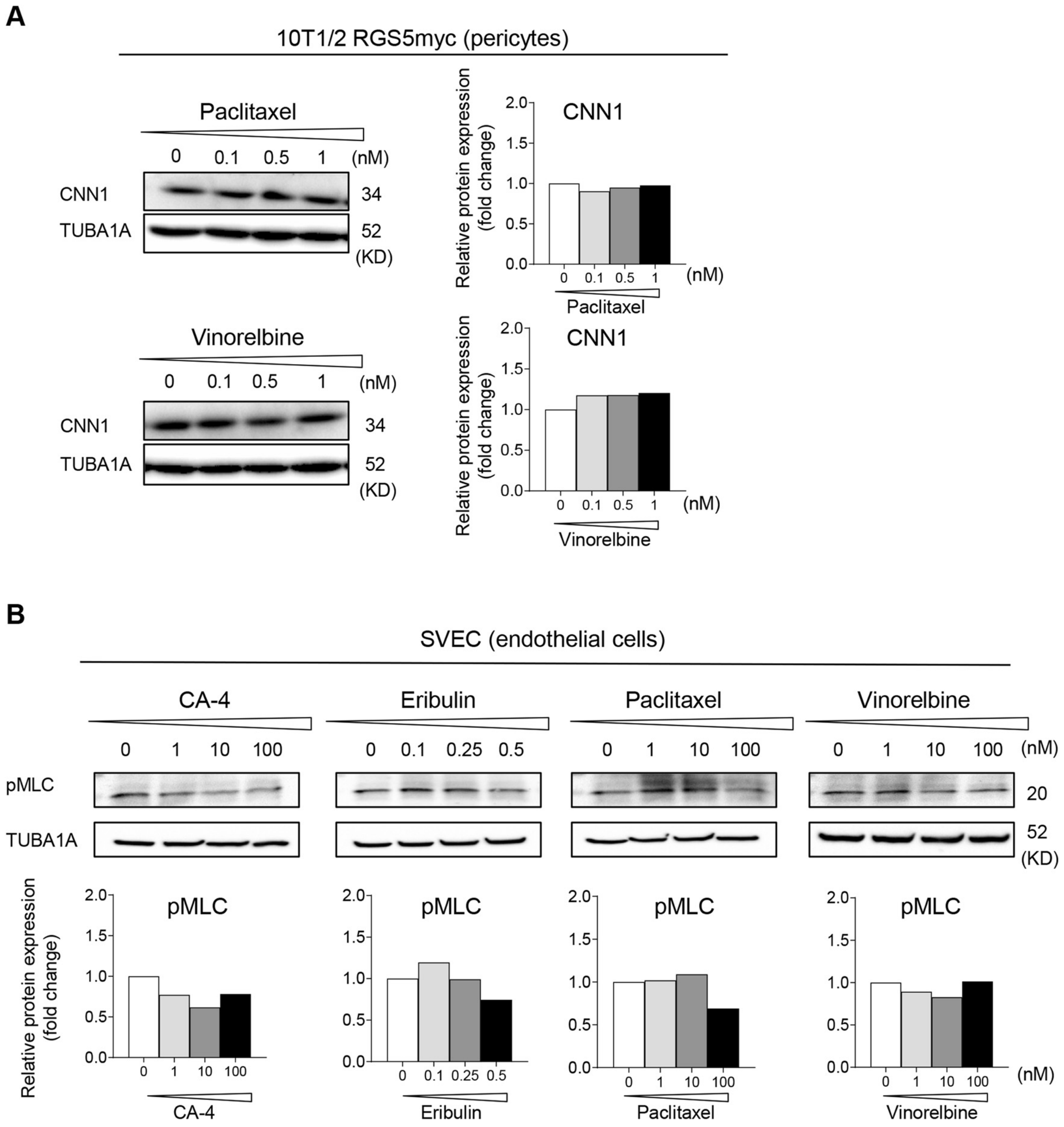

Figure EV1. Low dose paclitaxel or vinorelbine do not induce pericyte maturity and all microtubule-binding drugs fail to activate RhoA kinase in endothelial cells.

(**A**) Representative western blots from 10T1/2 RGS5myc cells incubated with indicated doses of paclitaxel or vinorelbine for 24 h. The contractile marker CNN1 intensities were quantified by densitometric analysis and normalized on tubulin intensity ($n = 1$). (**B**) Representative western blots from SVEC endothelial cells incubated with indicated doses of CA-4, eribulin, paclitaxel or vinorelbine for 24 h. pMLC intensities were quantified by densitometric analysis and normalized on tubulin intensity ($n = 1$).

© The Author(s)

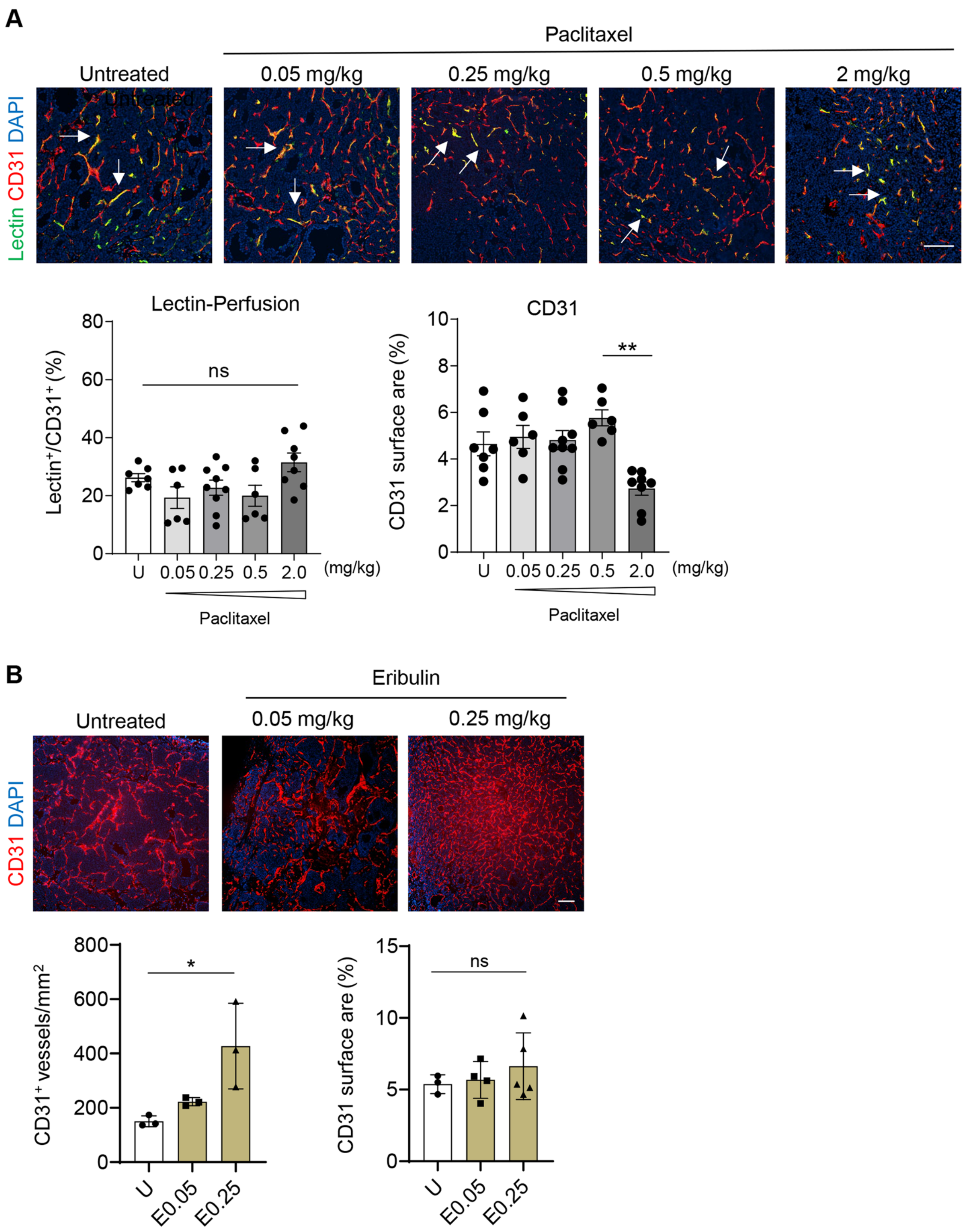

Figure EV2. Metronomic paclitaxel dosing and eribulin have vessel remodeling effects.

(**A**) Tumor-bearing RIP1-Tag5 mice were left untreated (U) or treated for 2 weeks with indicated doses of paclitaxel followed by i.v. infusion of FITC-lectin. Representative fluorescence micrographs showing FITC-lectin (green) overlay (yellow, arrows) with CD31 vascular (red) staining. Lectin perfusion and CD31 surface area (vascularity) were quantified, $n = 7$ tumors for untreated, $n = 6$ tumors for 50 mg/kg and 500 mg/kg, $n = 9$ tumors for 500 mg/kg and $n = 8$ tumors for 2 mg/kg paclitaxel. $^{**}P = 0.0081$, ns, not statistically significant. Data were analyzed by one-way ANOVA and expressed as mean ± SEM. Scale bar, 100 µm. (**B**) Tumor-bearing RIP1-Tag5 mice were treated with 0.05 mg/kg (E0.05) or 0.25 mg/kg (E0.25) eribulin for 2 weeks and tumors stained with the vascular marker CD31 (red). Representative fluorescence micrographs show examples of tumor vascularity under eribulin treatment. Microvascular density ($CD31^+$ vessels/mm^2, left, $n = 3$) and overall vascularity ($CD31^+$ surface area, right, $n = 3$ for U, $n = 4$ for E0.05, $n = 5$ for E0.25) were quantified, $^*P = 0.018$, E0.25 compared to untreated; ns, not statistically. Scale bar, 100 µm. Data were analyzed by one-way ANOVA and expressed as mean ± SEM.

 © The Author(s)

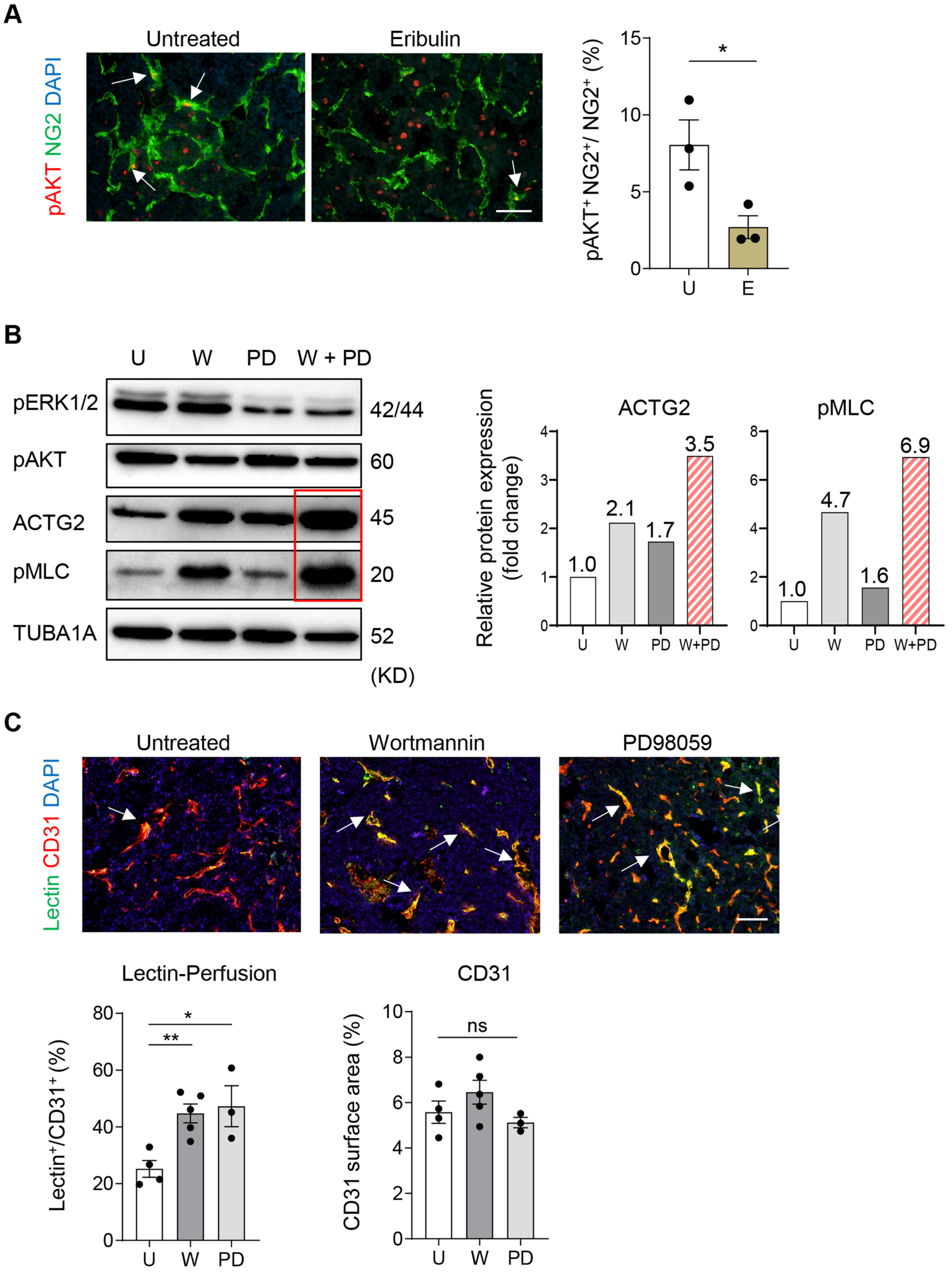
A
Untreated
Eribulin
pAKT NG2 DAPI
pAKT+ NG2+/ NG2+ (%)
15
10
5
0
*
U
E
B
U W PD W + PD
pERK1/2
pAKT
ACTG2
pMLC
TUBA1A
42/44
60
45
20
52
(KD)
Relative protein expression (fold change)
ACTG2
1.0
2.1
1.7
3.5
U W PD W+PD
pMLC
1.0
4.7
1.6
6.9
U W PD W+PD
C
Untreated
Wortmannin
PD98059
Lectin CD31 DAPI
Lectin-Perfusion
Lectin+/CD31+ (%)
80
60
40
20
0
*
**
U W PD
CD31
CD31 surface area (%)
10
8
6
4
2
0
ns
U W PD

Figure EV3. Specific inhibition of ERK and/or PI3K signaling induces pericyte maturity and improves tumor perfusion.

(**A**) Representative fluorescent micrographs show pAKT (red) expression in RIP1-Tag5 NG2$^+$ tumor pericytes (green). Arrows indicate overlay (yellow). Quantification of NG2$^+$ pericyte specific pAKT signals in untreated or eribulin treated tumors, $n = 3$ mice, *$P = 0.0402$. Data were analyzed using two-tailed, unpaired Student's *t* test and presented as mean ± SEM. Scale bar, 50 µm. (**B**) Representative western blots from 10T1/2 RGS5myc cells incubated with signaling pathway inhibitors for 24 h (U, untreated; W, Wortmannin, 10 µM; PD, PD98058, 20 µM; W + PD, combination of Wortmannin and PD98058). Phosphorylated ERK1/2 and AKT proteins are shown as controls for PD98058 and Wortmannin activities, respectively. Phosphorylated MLC intensities were quantified by densitometric analysis and normalized to tubulin intensity to assess changes in activation; ACTG2 intensities were quantified to determine contractile maker induction ($n = 1$). Red boxing highlights additive marker induction following W + PD treatment. One of 2 independent experiments is shown. (**C**) Tumor-bearing RIP1-Tag5 mice were left untreated (U, $n = 4$) or treated 3 x/week for 2 weeks with Wortmannin (W, 0.25 mg/kg, $n = 5$) or PD98059 (PD, 1 mg/kg, $n = 3$) followed by i.v. infusion of FITC-lectin. Representative fluorescence micrographs showing FITC-lectin (green) overlay with CD31 vascular (red) staining as surrogate marker for tumor perfusion (yellow, arrows). Lectin perfusion and CD31 surface area (vascularity) were quantified, *$P = 0.0135$, **$P = 0.0130$, ns, not statistically significant. Data were analyzed by one-way ANOVA and expressed as mean ± SEM. Scale bar, 100 µm.

© The Author(s)

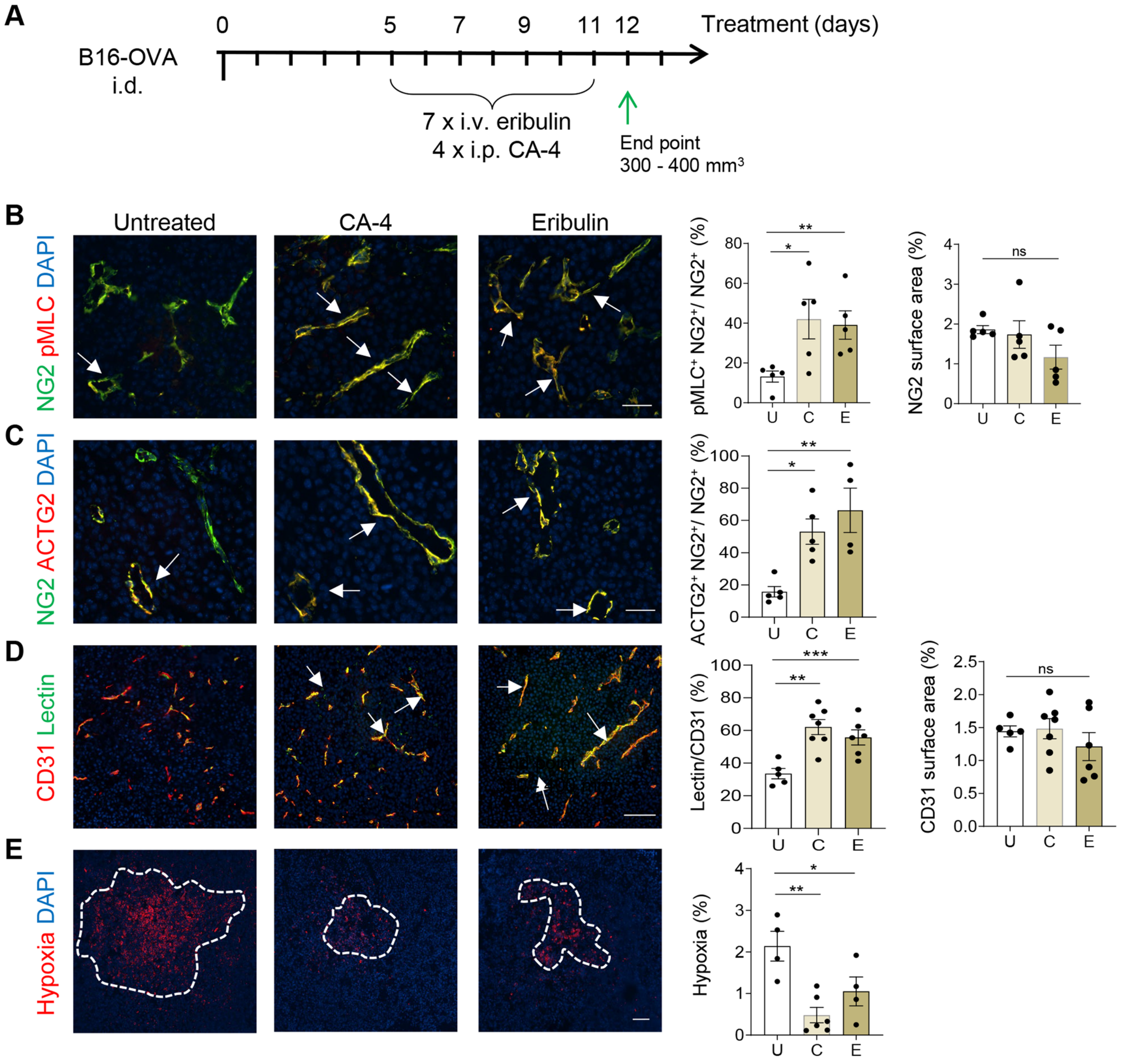

Figure EV4. CA-4 and eribulin induce pericyte phenotype switching in mouse melanoma.

(**A**) Treatment schedule of an orthotopic melanoma model (B16-OVA) and endpoint for immunohistochemistry. B16-OVA tumors were grown in untreated C57BL/6 mice (U), or mice treated with C-A4 (C, 2.5 mg/kg) or eribulin (E, 0.25 mg/kg). (**B**) Representative fluorescence micrographs show examples of drug-induced changes in pericyte pMLC expression. pMLC (red) coverage of $NG2^+$ pericytes (green) was quantified, arrows indicate overlay (yellow), $n = 5$, $^*P = 0.048$, $^{**}P = 0.028$, ns, not statistically significant. Scale bar, 50 μm. (**C**) Representative fluorescence micrographs and quantification of the contractile marker ACTG2 (red) expression in pericytes (NG2, green); arrows indicate overlay (yellow), $n = 5$ mice for U, C, $n = 4$ mice for E, $^*P = 0.016$, $^{**}P = 0.0034$. Scale bar, 50 μm. (**D**) Representative fluorescence micrographs and quantification of FITC-lectin (green) overlay (yellow, arrows) with $CD31^+$ (red) blood vessels as surrogate marker for tumor perfusion, $n = 5$ mice for U, $n = 7$ mice for C, $n = 6$ mice for E, $^{**}P = 0.007$, $^{***}P = 0.0007$, ns, not statistically significant. Scale bar, 100 μm. (**E**) Representative fluorescence micrographs and quantification of hypoxyprobe (red, white dashed line) as marker for tumor oxygen status, $n = 4$ mice for U, E, $n = 6$ mice for C, $^*P = 0.0477$, $^{**}P = 0.0025$. Scale bar, 100 μm. All data were analyzed using one-way ANOVA and expressed as mean ± SEM.

© The Author(s)

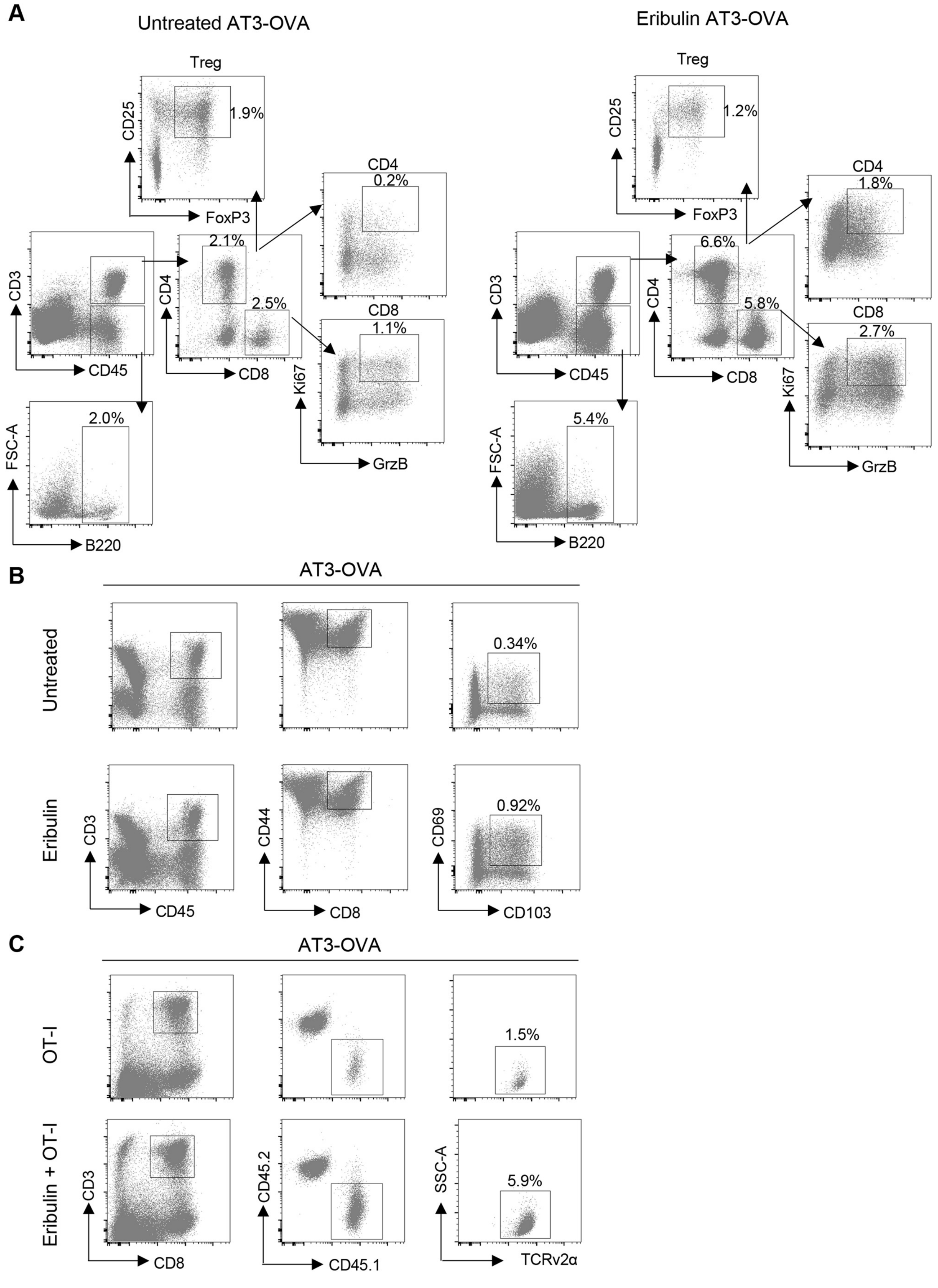
A
Untreated AT3-OVA
Eribulin AT3-OVA
Treg
CD25
FoxP3
1.9%
1.2%
CD4
0.2%
1.8%
CD8
1.1%
2.7%
2.1%
2.5%
6.6%
5.8%
CD3
CD45
CD4
CD8
Ki67
GrzB
FSC-A
B220
2.0%
5.4%
B
AT3-OVA
Untreated
Eribulin
0.34%
0.92%
CD3
CD45
CD44
CD8
CD69
CD103
C
AT3-OVA
OT-I
Eribulin + OT-I
1.5%
5.9%
CD3
CD8
CD45.2
CD45.1
SSC-A
TCRv2α

 © The Author(s)

Figure EV5. Eribulin treatment increases spontaneous and adoptively transferred effector T cells in the breast cancer microenvironment.

(**A**) Representative FACS blots showing gating strategy for FACS quantification of tumor-infiltrating $CD4^+$ T cells, effector $CD4^+$ T cells, T regs, $CD8^+$ T cells, effector $CD8^+$ T cells, and B cells in untreated (left) and eribulin treated AT3-OVA breast cancers (right). (**B**) Gating strategy for the detection of T_{RM} $CD8^+$ T cells in untreated and eribulin treated AT3-OVA tumors. (**C**) Representative FACS blots showing gating strategy for FACS quantification of adoptively transferred OT-I T cells in untreated or eribulin treated AT3-OVA breast cancers.

© The Author(s)

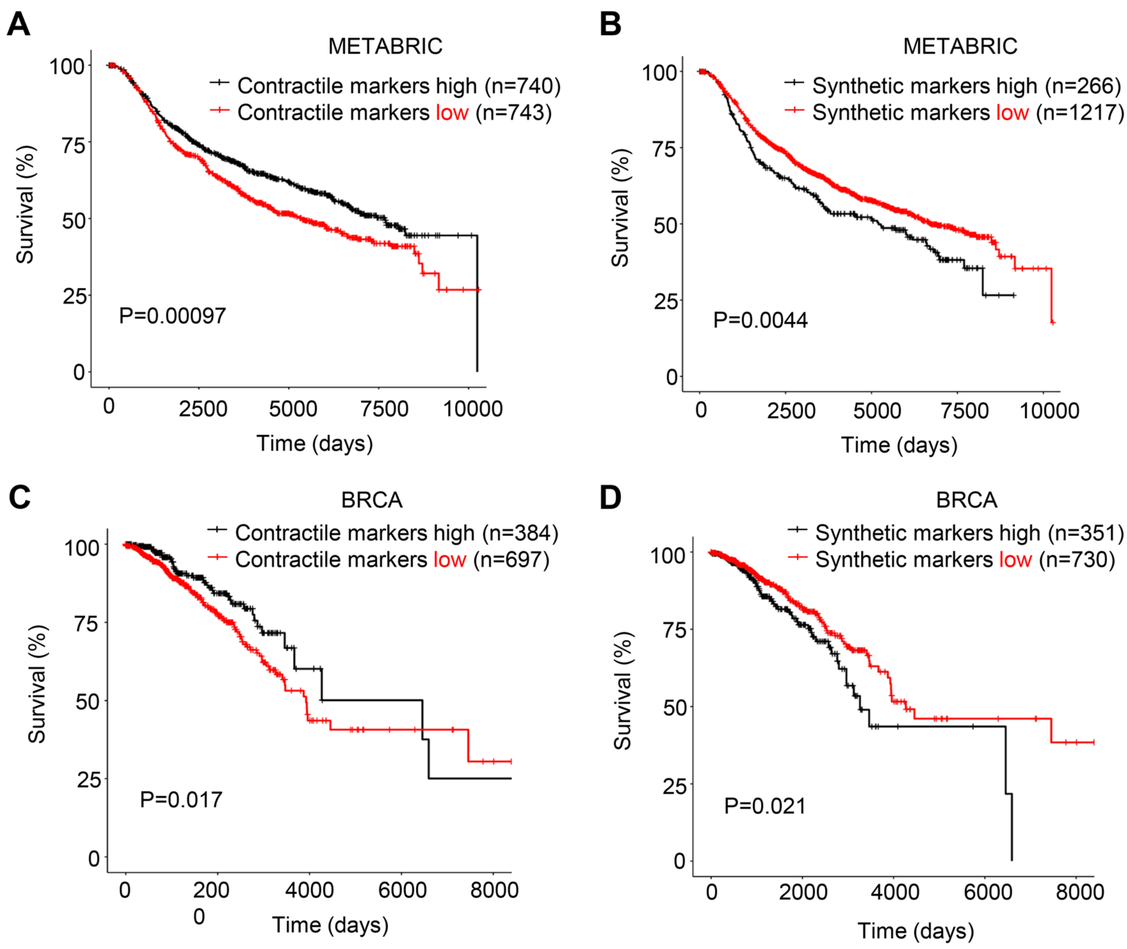

Figure EV6. Improved survival outcome in breast cancer patients correlates with a contractile pericyte gene signature.

(**A**, **B**) Kaplan–Meier curves showing prognostic value of (**A**) contractile and (**B**) synthetic pericyte gene signatures for disease progression in the METABRIC breast cancer patient cohort ($n = 1483$, 837 alive, 646 dead) using an optimal cutoff stratification to divide patients into high and low expression groups. Contractile gene signature: ACTG2, ACTA2, CNN1, CALD1, MYLK, MYH11, MYOCD, CDH5. Synthetic gene signature: NOTCH3, RGS5, KLF4, LMNB1, COL1A1, COL1A2. Log-rank test, (**A**) $P = 0.00097$; (**B**) $P = 0.0044$. (**C**, **D**) Kaplan–Meier curves showing prognostic value of (**C**) contractile and (**D**) synthetic pericyte gene signatures for disease progression in the BRCA breast cancer patient cohort ($n = 1083$, 932 alive, 151 dead) using an optimal cutoff stratification to divide patients into high and low expression groups. Contractile genes as above. Synthetic gene signature: NOTCH3, RGS5, KLF4, LMNB1, COL1A1. Log-rank test, (**C**) $P = 0.017$; (**D**), $P = 0.021$.

© The Author(s)
